# Ghrelin deletion and conditional ghrelin cell ablation increase pancreatic islet size in mice

**DOI:** 10.1172/JCI169349

**Published:** 2023-12-15

**Authors:** Deepali Gupta, Avi W. Burstein, Dana C. Schwalbe, Kripa Shankar, Salil Varshney, Omprakash Singh, Subhojit Paul, Sean B. Ogden, Sherri Osborne-Lawrence, Nathan P. Metzger, Corine P. Richard, John N. Campbell, Jeffrey M. Zigman

**Affiliations:** 1 Center for Hypothalamic Research, Department of Internal Medicine, UT Southwestern Medical Center, Dallas, Texas, USA.; 2 Department of Biology, University of Virginia, Charlottesville, Virginia, USA.; 3 Division of Endocrinology and Metabolism, Department of Internal Medicine and; 4 Department of Psychiatry, UT Southwestern Medical Center, Dallas, Texas, USA.

**Keywords:** Endocrinology, Metabolism, Beta cells, Islet cells, Obesity

## Abstract

Ghrelin exerts key effects on islet hormone secretion to regulate blood glucose levels. Here, we sought to determine whether ghrelin’s effects on islets extend to the alteration of islet size and β cell mass. We demonstrate that reducing ghrelin — by ghrelin gene knockout (GKO), conditional ghrelin cell ablation, or high-fat diet (HFD) feeding — was associated with increased mean islet size (up to 62%), percentage of large islets (up to 854%), and β cell cross-sectional area (up to 51%). In GKO mice, these effects were more apparent in 10- to 12-week-old mice than in 4-week-old mice. Higher β cell numbers from decreased β cell apoptosis drove the increase in β cell cross-sectional area. Conditional ghrelin cell ablation in adult mice increased the β cell number per islet by 40% within 4 weeks. A negative correlation between islet size and plasma ghrelin in HFD-fed plus chow-fed WT mice, together with even larger islet sizes in HFD-fed GKO mice than in HFD-fed WT mice, suggests that reduced ghrelin was not solely responsible for diet-induced obesity–associated islet enlargement. Single-cell transcriptomics revealed changes in gene expression in several GKO islet cell types, including upregulation of *Manf*, *Dnajc3*, and *Gnas* expression in β cells, which supports decreased β cell apoptosis and/or increased β cell proliferation. These effects of ghrelin reduction on islet morphology might prove useful when designing new therapies for diabetes.

## Graphical abstract

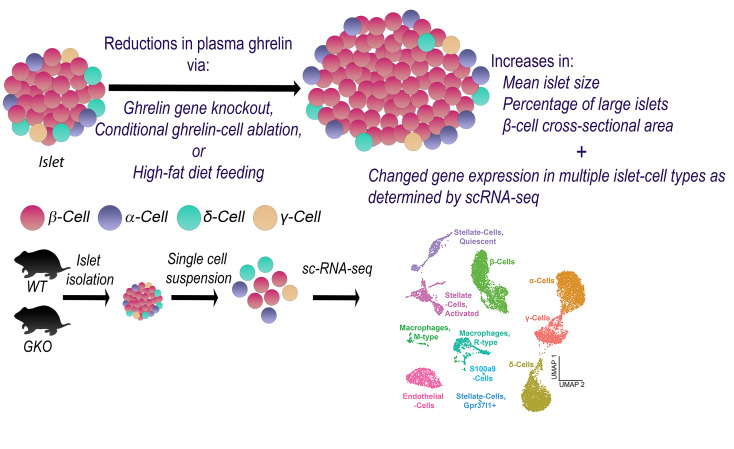

## Introduction

Ghrelin is secreted primarily by gastric ghrelin cells (1). It is mainly via binding to CNS and pituitary growth hormone secretagogue receptors (GHSRs) that ghrelin’s actions to increase GH secretion, food intake, and BW are exerted (1–4). GHSR expression within all 4 traditional pancreatic islet endocrine cell types — including most prominently somatostatin-secreting δ cells, and also glucagon-secreting α cells, insulin-secreting β cells, and pancreatic polypeptide-secreting γ cells — suggests that ghrelin also affects blood glucose levels (5–8). Indeed, ghrelin raises blood glucose levels when administered, permits the normal counterregulatory response to insulin-induced hypoglycemia, and prevents life-threatening drops in blood glucose during severe caloric restriction (9–12). In the latter setting, ghrelin’s glucoregulatory actions are mediated largely by GH (11, 12). In other settings, ghrelin’s glucoregulatory actions probably involve more direct effects on islet hormone secretion, including increased somatostatin secretion, increased glucagon secretion, and/or decreased insulin secretion (6, 13). Ghrelin also decreases islet blood flow, whereas GHSR antagonism does the opposite (14).

It is also possible that ghrelin influences blood glucose levels via additional effects on islets. For instance, ghrelin-secreting ε cells are found in fetal and adult human islets and in mice from embryonic stages until approximately 2 weeks of age (15). Specifically, ε cells are plentiful in fetal human islets (up to ~10%–30% of fetal human islet cells) and fetal mouse islets, although their numbers drop to only the occasional adult human islet cell and are undetectable in mice older than 2 weeks (5, 15–21). The existence of ε cells in embryonic islets and islet GHSR expression, coupled with the finding of increased ghrelin-positive islet cells upon knockout of transcription factors mediating islet endocrine cell-type differentiation (22, 23), suggests roles for ghrelin in islet development and/or islet growth. Hill et al. (23) directly investigated these possibilities by methodically comparing cross-sectional areas of embryonic islets from WT versus ghrelin-knockout (GKO) mice. However, islet area, insulin immunoreactivity (IR), glucagon IR, somatostatin IR, and pancreatic polypeptide IR were unaffected.

At least 9 other studies describe the effects of manipulating ghrelin or GHSR on islet size. Dezaki et al. (24) examined islets from 8-week-old WT and GKO mice and observed no effect of genotype on islet density, numbers, area, or diameter. Kurashina et al. (25) demonstrated equivalent islet areas in WT and GHSR-null mice. Pradhan et al. (26) reported no obvious differences in islet size in adult WT versus GHSR-KO mice, although insulin staining was subjectively denser in GHSR-KO mice. Ma et al. (27) reported that islet area in *ob/ob* (leptin-deficient) mice, which was more than double that in WT mice, was even higher in *ob/ob* mice on a GKO background, but was not greater in *ob/ob* mice on a GHSR-KO background. Also, islets from *ob/ob* mice and *ob/ob* mice on a GHSR-KO background were subjectively more vascularized than were islets from WT mice.

Bando et al. (28) found no genotype-dependent effect on the insulin-positive area/islet in 7-week-old mice with rat insulin II promoter–driven ghrelin and ghrelin-*O*-acyltransferase overexpression. Yet, following streptozotocin (STZ) treatment, which reduces β cell numbers and causes hyperglycemia, transgenic mice exhibited an increase in insulin-positive area/islet and insulin-positive cells/islet compared with WT mice. Granata et al. (29) examined islets from 70-day-old rats injected with buffer versus STZ just after birth, with or without subsequent ghrelin administration. Ghrelin did not affect islet density or area in buffer-treated rats but significantly raised both in STZ-treated rats. In Shankar et al. (10), we observed no subjective differences in shape, size, or patterns of insulin IR or glucagon IR of randomly selected islets from STZ-treated GKO and WT littermates.

Mosa et al. (30) analyzed islets from 7- to 10-week-old nonobese diabetic MKR (muscle IGF-1 receptor-lysine-arginine) mice and WT control mice that received 12 days of GHSR antagonist ([D-Lys3]-GHRP-6) or saline. [D-Lys3]-GHRP-6, which worsened glucose and insulin tolerance in the MKR mice, reduced the insulin IR area/islet, and raised the somatostatin IR area/islet in both MKR and control mice. Baena-Nieto et al. (31) administered ghrelin to BB rats, which represent an autoimmune model of type 1 diabetes mellitus (T1DM). Ghrelin attenuated the decreases in total islet area/total pancreatic area and total number of islets/pancreatic area, reduced mononuclear cell infiltration of islets, and blocked the usual decrease in β cell proliferation observed in vehicle-treated BioBreeding (BB) rats.

Thus, transgenic or pharmacologic maneuvers that reduce ghrelin and/or GHSR signaling either had no effect on islet size, were associated with increased islet size or islet insulin staining, or reduced the insulin IR per islet. Transgenic or pharmacologic methods that raise ghrelin and/or increase GHSR signaling either exerted no effect on islet size and density or increased the insulin IR per islet or islet size and density. These seemingly conflicting findings may have resulted from the varying degrees of scientific rigor utilized (Supplemental Table 1; supplemental material available online with this article; https://doi.org/10.1172/JCI169349DS1) and/or variability in the contexts in which those manipulations were made. In the current study, we used a rigorous approach to characterize the effects of lowering ghrelin levels in mice — as achieved by either germline ghrelin deletion or conditional ghrelin cell ablation in adults — on islet morphology. Furthermore, we aimed to determine whether the reduced plasma ghrelin levels induced by chronic high-fat diet (HFD) feeding contributes to the islet enlargement associated with diet-induced obesity.

## Results

### Ghrelin deletion increases islet size in adult mice.

**Figure 1:**
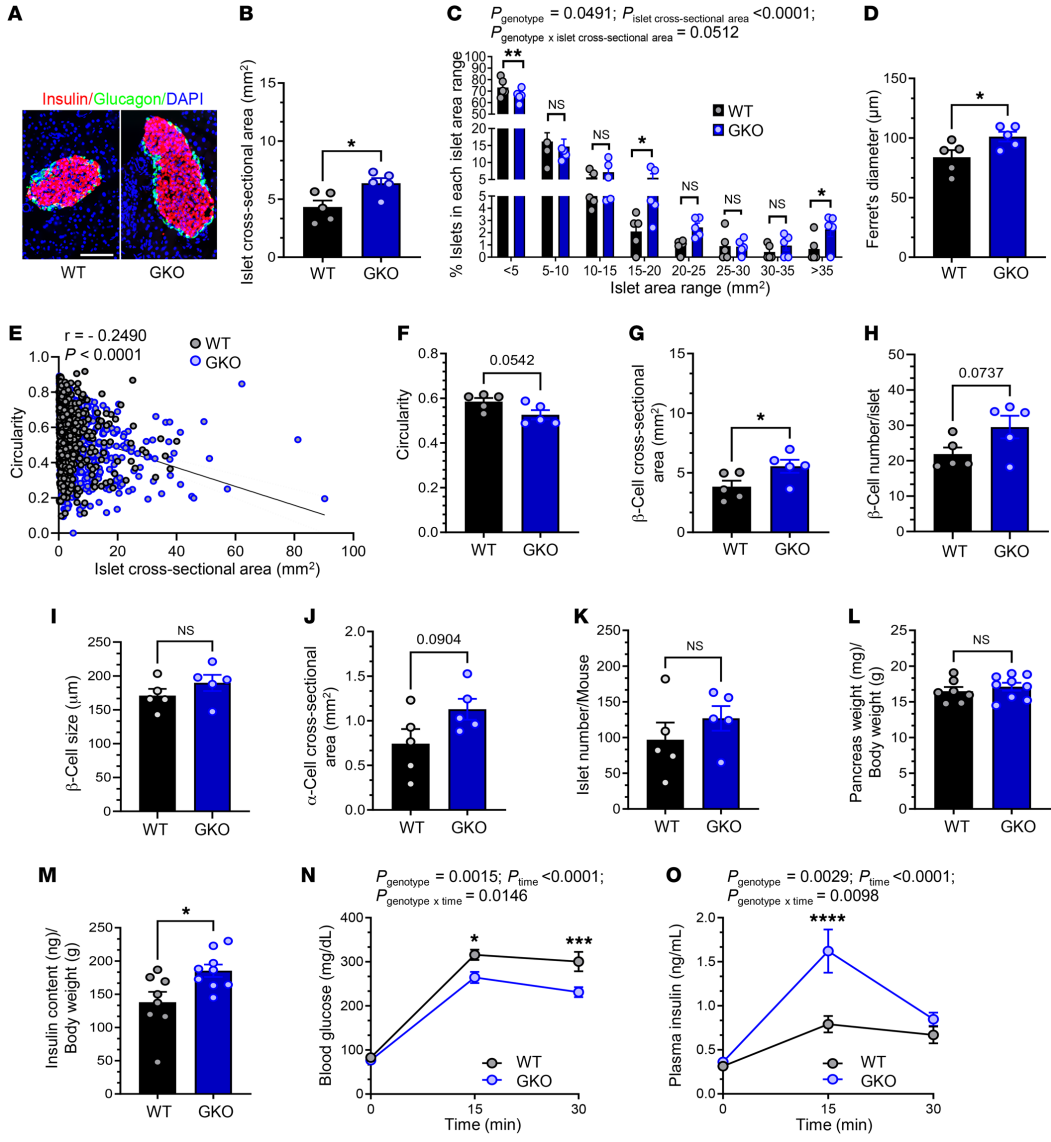
Ghrelin deletion increases islet size in adult mice. (**A**) Representative islet images from 10- to 12-week-old (adult) WT mice and GKO littermates. Scale bar: 100 μm. (**B**) Islet cross-sectional area. (**C**) Percentage of islets in each islet cross-sectional area range, in which each range except the last represents a 5 mm^2^ interval. (**D**) Ferret’s diameter of islets. (**E**) Correlation between islet cross-sectional area and circularity. (**F**) Circularity of islets. (**G**) β Cell cross-sectional area per islet. (**H**) β Cell number per islet. (**I**) β Cell size. (**J**) α Cell cross-sectional area per islet. (**K**) Number of islets, as counted in 4 head-to-tail pancreatic sections per mouse. (**L**) Pancreas weight per BW. (**M**) Pancreatic insulin content. (**N**) Blood glucose and (**O**) plasma insulin at 0, 15, and 30 minutes after administration of glucose (2 g/kg BW by gavage). *n* = 5 mice (**A**–**K**); *n* = 7–9 mice (**L** and **M**); *n* = 8–17 mice (**N** and **O**). Data were analyzed by unpaired, 2-tailed Student’s *t* test (**B**, **D**, **F**–**M**), 2-way repeated measures ANOVA followed by Bonferroni’s multiple-comparison test (**C**, **N**, **O**), and Pearson’s correlation coefficient (*r*) (**E**). *P < 0.05, **P < 0.01, ***P < 0.001, and ****P < 0.0001; actual *P* values of 0.05 or greater and of less than 0.1 are indicated.

We first studied 10- to 12-week-old GKO mice and WT littermates (adults). Nearly all islets within four 8 μm thick head-to-tail pancreas sections (each section separated from the next by ≥50 μm) from each of 5 mice per genotype were examined by an investigator blinded to the genotype. This amounted to 484 WT and 634 GKO islets (see Table 1 for details). The overall organization of the islet (centrally distributed insulin IR β cells and peripheral glucagon IR α cells; Figure 1A) and consistent pattern of islet distribution throughout the pancreas were similar in WT and GKO mice. However, the mean islet cross-sectional area was approximately 47% higher in GKO mice than in WT mice (Figure 1B). This was driven in GKO mice by increases in the percentage of mid-sized islets (15–20 mm^2^; ~164% increase) and very large islets (>35 mm^2^; ~240% increase) and a decrease in the percentage of the smallest islets (<5 mm^2^; ~10% decrease) (Figure 1C). Mean Ferret’s diameter, which is the longest diameter within an islet and thus serves as another indicator of islet size, was approximately 21% higher in GKO mice (Figure 1D). A strong negative correlation was observed between islet circularity (shape) and islet cross-sectional area, whether islets from both genotypes were grouped together (Figure 1E) or separately (WT: *r* = –0.1984, *P* < 0.0001; GKO: *r* = –0.2623, *P* < 0.0001). Consistent with their greater mean islet size, GKO mice exhibited approximately 10% lower mean circularity (*P* = 0.0542) (Figure 1F). GKO islets exhibited an approximately 45% higher mean β cell cross-sectional area (Figure 1G), which was the result of an approximately 35% increase in the number of β cells per islet (*P* = 0.0737) without effects on mean β cell size (Figure 1, H and I). GKO mice also had an approximately 52% higher α cell cross-sectional area (Figure 1J, *P* = 0.0904). The number of islets (counted per 4 head-to-tail pancreatic sections per mouse) was comparable between genotypes (Figure 1K), as were the pancreatic cross-sectional area and the pancreas weight per BW (Figure 1L and Supplemental Figure 1A).

Consistent with the increased β cell area, pancreatic insulin content was approximately 35% higher in GKO mice (Figure 1M). As previously reported (6, 32), GKO mice exhibited improved glucose tolerance, as indicated by an attenuated rise in blood glucose at 15 minutes and 30 minutes following administration of glucose (2 g/kg BW by gavage), and a corresponding accentuated rise in plasma insulin 15 minutes after gavage (by ~347% in GKO mice vs. ~152% in WT mice) (Figure 1, N and O). BWs, blood glucose levels, and plasma insulin levels of ad libitum–fed WT and GKO mice were similar; plasma ghrelin was undetectable in GKO mice (Supplemental Figure 1, B–E).

### Ghrelin deletion increases islet size in juvenile mice.

**Figure 2:**
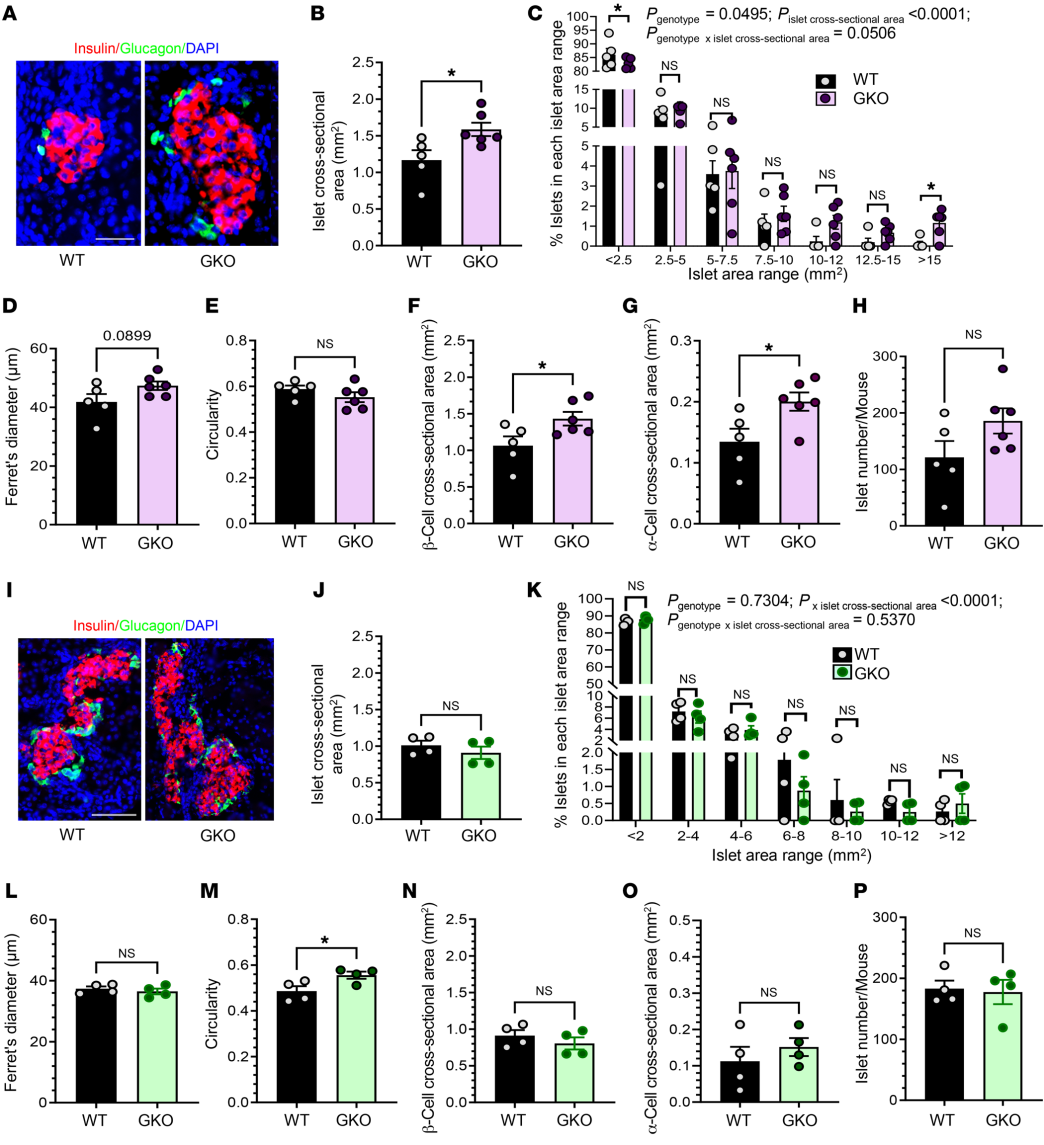
Ghrelin deletion increases islet size in juvenile but not neonate mice. (**A**) Representative images of islets from 4-week-old (juvenile) WT mice and GKO littermates. (**B**) Cross-sectional area of islets from juvenile mice. (**C**) Percentage of islets from juveniles in each islet cross-sectional area range, in which each range except the last represents a 2.5 mm^2^ interval. (**D**) Ferret’s diameter of islets from juvenile mice. (**E**) Circularity of juvenile mouse islets. (**F**) β Cell cross-sectional area per islet in juvenile mice. (**G**) α Cell cross-sectional area per islet in juvenile mice. (**H**) Number of islets from juvenile mice, as counted in 4 head-to-tail pancreatic sections per mouse. (**I**) Representative images of islets from P0–P2 WT mice and GKO littermates (neonates). (**J**) Cross-sectional area of islets from neonates. (**K**) Percentage of islets from neonates in each islet cross-sectional area range, in which each range except the last represents a 2 mm^2^ interval. (**L**) Ferret’s diameter of islets. (**M**) Circularity of islets. (**N**) β Cell cross-sectional area per islet. (**O**) α Cell cross-sectional area per islet. (**P**) Number of islet cells, as counted in 4 head-to-tail pancreatic sections per mouse. Scale bar: 50 μm (**A** and **I**). *n* = 5–6 mice (**A**–**H**); *n* = 4 mice (**I**–**P**). Data were analyzed by unpaired, 2-tailed Student’s *t* test (**B**, **D**–**J**, **L**–**P**) and 2-way, repeated-measures ANOVA followed by Bonferroni’s multiple-comparison test (**C** and **K**). *P < 0.05; actual *P* values of 0.05 or greater and of less than 0.1 are indicated.

We used the above-described methods to analyze islets from 4-week-old WT and GKO littermate mice (juveniles, *n* = 5–6 mice/genotype). We examined a total of 607 WT and 1,115 GKO islets (Table 1). We found that the juvenile mice had a genotype-independent overall structural organization of the islets (Figure 2A) and that the islets were equivalently distributed throughout the pancreas. Mean islet cross-sectional area was approximately 36% higher in GKO mice than in WT mice (Figure 2B). This was driven in GKO mice by an increase in the percentage of very large islets (>15 mm^2^, ~854% increase) and a decrease in the percentage of the smallest islets (<2.5 mm^2^, ~6% decrease) (Figure 2C). Mean Ferret’s diameter was approximately 13% higher in GKO mice (*P* = 0.0899; Figure 2D), but circularity was genotype independent (Figure 2E). GKO islets exhibited an approximately 35% higher mean β cell cross-sectional area (Figure 2F) and an approximately 49% higher α cell cross-sectional area (Figure 2G). The number of islets (within the 4 pancreatic sections) per mouse was comparable between genotypes (Figure 2H). BWs of ad libitum–fed 4-week-old WT and GKO mice were similar (Supplemental Figure 2A).

### Ghrelin deletion does not change islet size in neonates.

We also examined islets from P0–P2 WT and GKO littermate mice (neonates, *n* = 4 mice/genotype). We studied a total of 732 WT and 710 GKO islets (Table 1). Although neonatal islets were less rounded in shape than those from adult and juvenile mice, the same pattern of central insulin IR and peripheral glucagon IR was present (Figure 2I). No significant genotype-dependent differences in islet cross-sectional area or Ferret’s diameter (Figure 2, J–L) were noted. Circularity was approximately 14% higher in GKO mice (Figure 2M). β Cell and α cell cross-sectional areas and number of islets (within the 4 pancreatic sections) per mouse were genotype independent (Figure 2, N–P). BWs of P0–P2 WT and GKO mice were similar (Supplemental Figure 2B).

### Age enhances the effects of ghrelin deletion on islet size.

**Figure 3:**
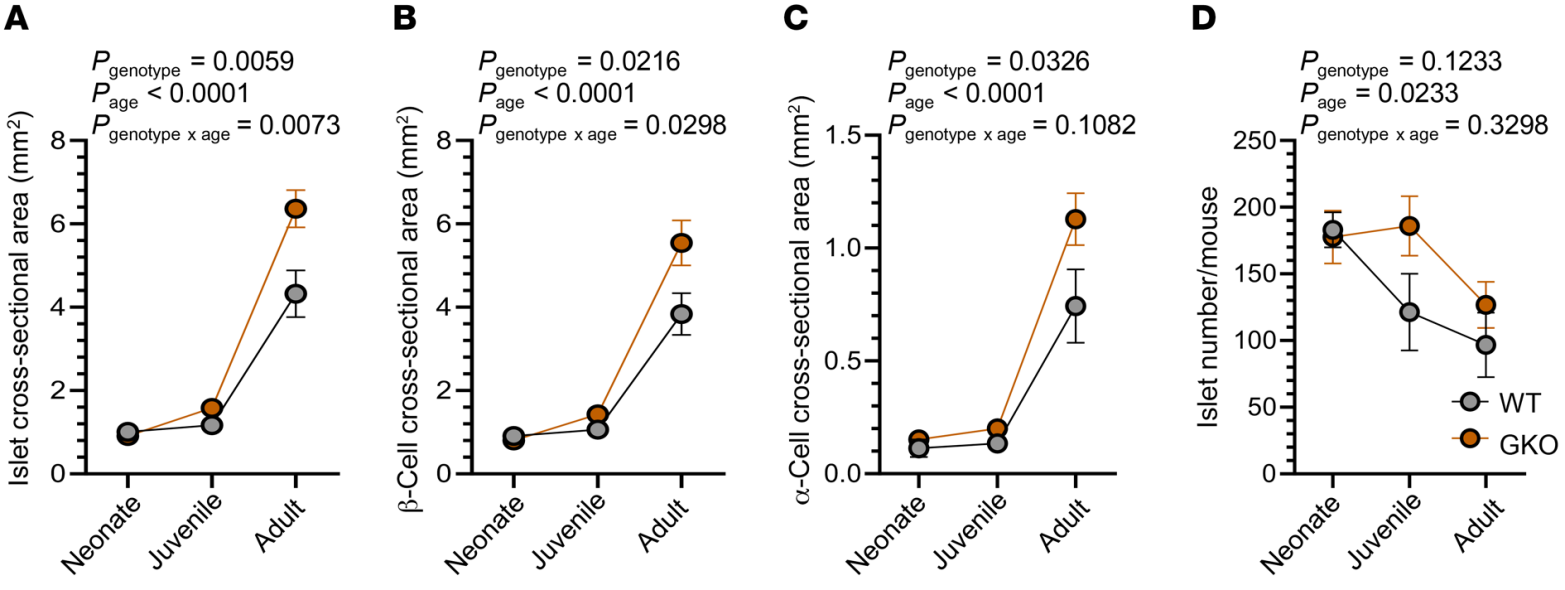
Age enhances the effects of ghrelin deletion on islet size. (**A**) Islet cross-sectional area, (**B**) β cell cross-sectional area per islet, (**C**) α cell cross-sectional area per islet, and (**D**) number of islets (as counted in 4 head-to-tail pancreatic sections per mouse) in WT and GKO neonates (P0–P2), juveniles (4 weeks of age), and adults (10–12 weeks of age). *n* = 4–6 mice. Data were analyzed by 2-way ANOVA followed by Bonferroni’s multiple-comparison test (*P* values are indicated in each panel).

We reanalyzed the islet, β cell, and α cell cross-sectional areas (Figure 3, A–C) and number of islets per mouse (Figure 3D) for neonates, juveniles, and adults using both genotype and age as factors. We found that both the GKO genotype and higher age independently increased islet, β cell, and α cell cross-sectional areas. For islet cross-sectional area and β cell cross-sectional area, there also was a significant genotype × age interaction, illustrative of a greater effect of ghrelin deletion on increasing islet size and β cell mass in older mice. Further, higher age was associated with a reduction in the number of islets within the 4 pancreatic sections from each mouse, without effects of genotype or genotype × age.

### Conditional ghrelin cell ablation in adult mice increases islet size.

**Figure 4:**
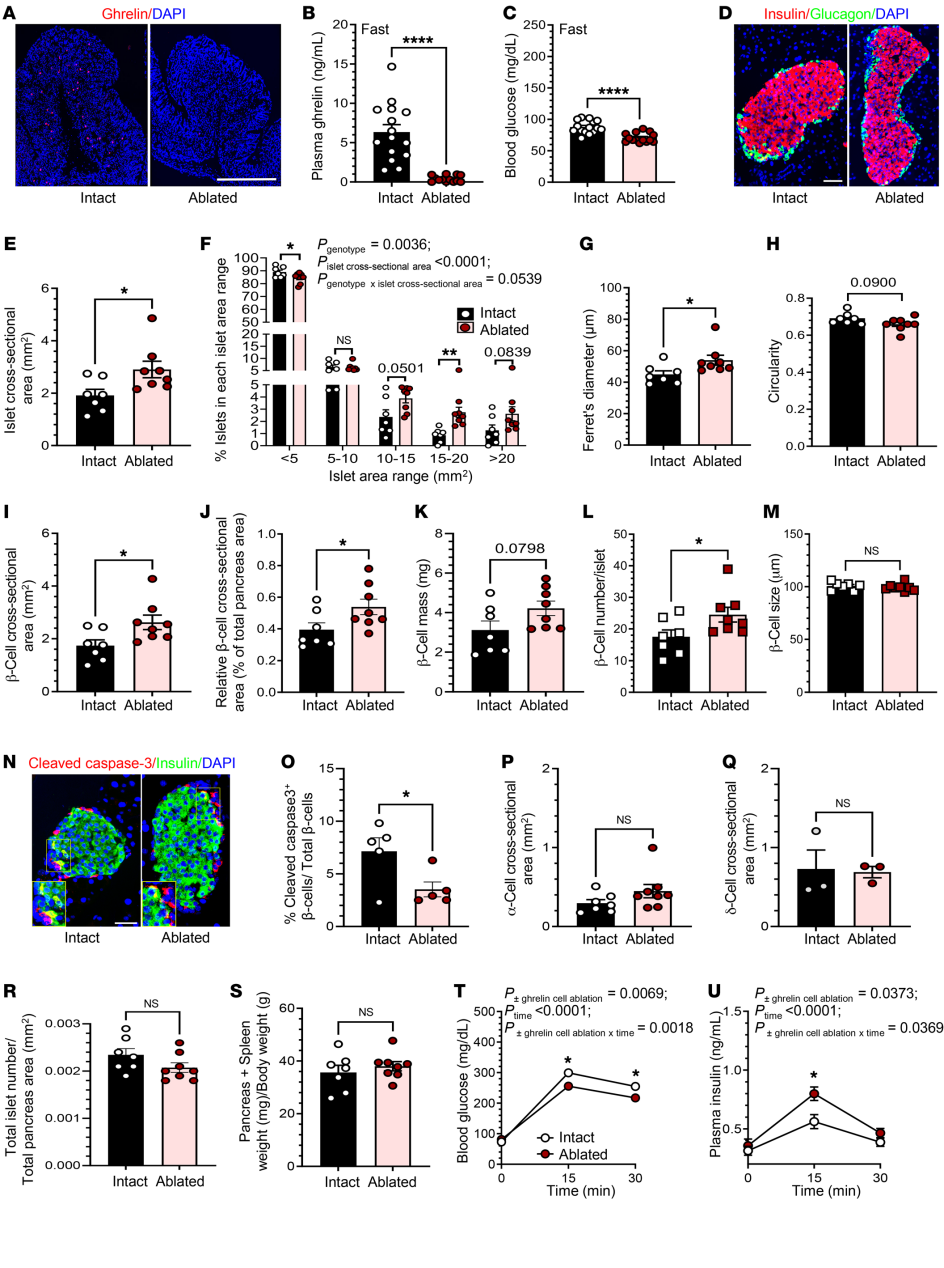
Inducible ghrelin cell ablation increases islet size in adult mice. (**A**) Representative images of stomach sections stained for ghrelin IR (in red) and with DAPI (nuclear staining, in blue) from mice with intact ghrelin cells or ablated ghrelin cells. (**B**) Plasma ghrelin and (**C**) blood glucose levels after a 24-hour fast. (**D**) Representative islet images from intact and ablated groups. (**E**) Islet cross-sectional area. (**F**) Percentage of islets in each islet cross-sectional area range, in which each range except the last represents a 5 mm^2^ interval. (**G**) Ferret’s diameter of islets. (**H**) Circularity of islets. (**I**) β Cell cross-sectional area per islet. (**J**) Relative β cell cross-sectional area. (**K**) β Cell mass. (**L**) Number of β cells per islet. (**M**) β Cell size. (**N**) Representative images of islets stained for cleaved caspase-3 IR (in red), insulin IR (in green), and DAPI (nuclear staining, in blue) from mice with intact ghrelin cells or ablated ghrelin cells. (**O**) Percentage of cleaved caspase-3^+^ β cells per total β cells. (**P**) α Cell cross-sectional area per islet. (**Q**) δ Cell cross-sectional area per islet. (**R**) Total number of islets per total pancreas area (within 4 pancreas sections per mouse). (**S**) Pancreas plus spleen weight per BW. (**T**) Blood glucose and (**U**) plasma insulin levels at various time points after glucose administration. Scale bars: 100 μm (**A**) and 50 μm (**D** and **N**). *n* = 3 mice (**A** and **Q**), *n* = 14–16 mice (**B** and **C**), *n* = 7–8 mice (**E**–**M**, **P**, **R**, and **S**), *n* = 5 mice (**N** and **O**), and *n* = 7–10 mice (**T** and **U**). Data were analyzed by unpaired, 2-tailed Student’s *t* test (**B**, **C**, **E**, **G**–**M**, and **O**–**S**) and 2-way repeated-measures ANOVA followed by Bonferroni’s multiple-comparison test (**F**, **T**, and **U**). *P < 0.05, **P < 0.01, ***P < 0.001, and ****P < 0.0001; actual *P* values of 0.05 or greater and of less than 0.1 are indicated.

To confirm that the changed islet morphology in GKO mice was not simply a manifestation of a developmental change induced by the germline deletion of the preproghrelin gene, we conditionally ablated ghrelin cells from 6-week-old mice and then examined islets 4 weeks after ablation. This was achieved by administering diphtheria toxin (DTX) to mice harboring a ghrelin-Cre transgene plus a Cre-inducible DTX receptor (iDTR) transgene (33). This strategy was previously shown to reduce plasma ghrelin levels by 50% within 8 hours, 86% within 24 hours, and by up to 95% subsequently; a reduction of greater than 80% was maintained for at least 4 weeks (34). Ghrelin cell ablation was confirmed by histological analysis (Figure 4A), which demonstrated a marked reduction of gastric ghrelin IR in double-transgenic mice that received DTX 4 weeks earlier versus “intact” mice (iDTR carriers lacking ghrelin-Cre that received DTX), and by serological analysis (Figure 4B and Supplemental Figure 3, A and C), which revealed reductions in plasma ghrelin in “ablated” mice (by 93.6% in the fed state 2 weeks after DTX, by 93.1% in the 24-hour-fasted state 3 weeks after DTX, and by 73.4% in the fed state 4 weeks after DTX). Furthermore, as expected (6) in mice with reduced ghrelin, 24-hour-fasted blood glucose levels were reduced, whereas ad libitum–fed blood glucose levels were unchanged in ablated mice (Figure 4C and Supplemental Figure 3, B and D). BWs were unaffected (Supplemental Figure 3E).

We analyzed 2,358 islets from 7 intact mice and 2,716 islets from 8 ablated mice (Table 1). The overall organization of their islets was similar (Figure 4D). Mean islet cross-sectional area and relative islet cross-sectional area/total pancreas area were approximately 52% and approximately 36% higher, respectively, in ablated mice than in intact mice (Figure 4E and Supplemental Figure 4A). This was driven in ablated mice by increases in the percentage of mid-sized islets (10–15 mm^2^, ~64% increase, *P* = 0.0501, and 15–20 mm^2^, ~221% increase) and very large islets (>20 mm^2^, ~107% increase, *P* = 0.0839) and by a decrease in the percentage of the smallest islets (<5 mm^2^, ~5% decrease) (Figure 4F). Mean Ferret’s diameter was approximately 20% higher in ablated mice, while mean circularity was approximately 5% lower (*P* = 0.0900) (Figure 4, G and H). Islets from ablated mice had an approximately 51% higher mean β cell cross-sectional area, an approximately 37% higher relative β cell cross-sectional area per total pancreas area, and an approximately 37% higher β cell mass (*P* = 0.0798) (Figure 4, I–K), which was the result of an approximately 40% increase in the number of β cells per islet without effects on β cell size (Figure 4, L and M).

Changes in β cell apoptosis, changes in β cell proliferation, and islet cell reprogramming have all been identified as potential contributors to increased β cell mass (35–37). Here, we investigated the first 2 of these mechanisms: β cell apoptosis, by determining the expression of cleaved caspase-3 IR within β cells in a subset of the above intact versus ablated mice, and β cell proliferation, by determining the expression of Ki67 IR within β cells in a subset of the above intact versus ablated mice, and by determining BrdU incorporation by β cells within a new cohort of intact versus ablated mice (this time, only 11 days after DTX administration). For the cleaved caspase-3 studies, we analyzed 69–89 islets per mouse (all islets within 1 head-to-tail pancreatic section/mouse, comprising a total of 398 islets; 1,377–2,812 β cells/mouse) from 5 intact mice and 64–113 islets per mouse (all islets within 1 head-to-tail pancreatic section/mouse, comprising a total of 464 islets; 1,781–2,585 β cells/mouse) from 5 ablated mice. The percentage of total β cells that were cleaved caspase-3^+^ was approximately 50.7% lower in the ablated mice than in the intact mice, suggesting reduced β cell apoptosis upon ghrelin reduction (Figure 4, N and O). For the Ki67 studies, we analyzed 152–198 islets per mouse (all islets within 2 head-to-tail pancreatic section/mouse, comprising a total of 863 islets; 3,443–5,632 β cells/mouse) from 5 intact mice and 157–203 islets per mouse (all islets within 2 head-to-tail pancreatic section/mouse, comprising a total of 713 islets; 3,529–5,808 β cells/mouse) from 4 ablated mice. We observed no significant difference in the percentage of total β cells that were Ki67^+^ between the ablated and intact mice, suggesting no detectable effects of reducing ghrelin on β cell proliferation (Supplemental Figure 5, A and B). For the BrdU studies, we analyzed a total of 234–313 islets per mouse (all islets within 2 head-to-tail pancreatic section/mouse, comprising a total of 1,038 islets; 4,759–7,588 β cells/mouse) from 4 intact mice and 199–410 islets per mouse (all islets within 2 head-to-tail pancreatic section/mouse, comprising a total of 817 islets; 5,098–9,473 β cells/mouse) from 3 ablated mice. Similar to the Ki67 findings in islets examined 4 weeks after DTX administration, the percentage of BrdU^+^ β cells was comparable between the ablated and intact mice (Supplemental Figure 5, C and D). Unlike at the 4-week time point, at this time point (11 days after DTX), we observed no differences in β cell number per islet (Supplemental Figure 5E).

Ablation of ghrelin cells did not affect α cell or δ cell cross-sectional areas, islet numbers per mouse (from 4 head-tail pancreas sections), total islet number per total pancreas area from 4 head-tail pancreas sections, or the combined pancreas plus attached spleen weights per BW (Figure 4, P–S, and Supplemental Figure 4B). Mice with ablated ghrelin cells exhibited improved glucose tolerance, as indicated by an attenuated rise in blood glucose at 15 minutes and 30 minutes following administration of glucose (2 g/kg BW by gavage) and a corresponding greater rise in plasma insulin levels 15 minutes after gavage (by ~124% in ablated mice vs. ~79% in intact mice; Figure 4, T and U).

### Ghrelin deletion further increases islet size in mice with diet-induced obesity.

**Figure 5:**
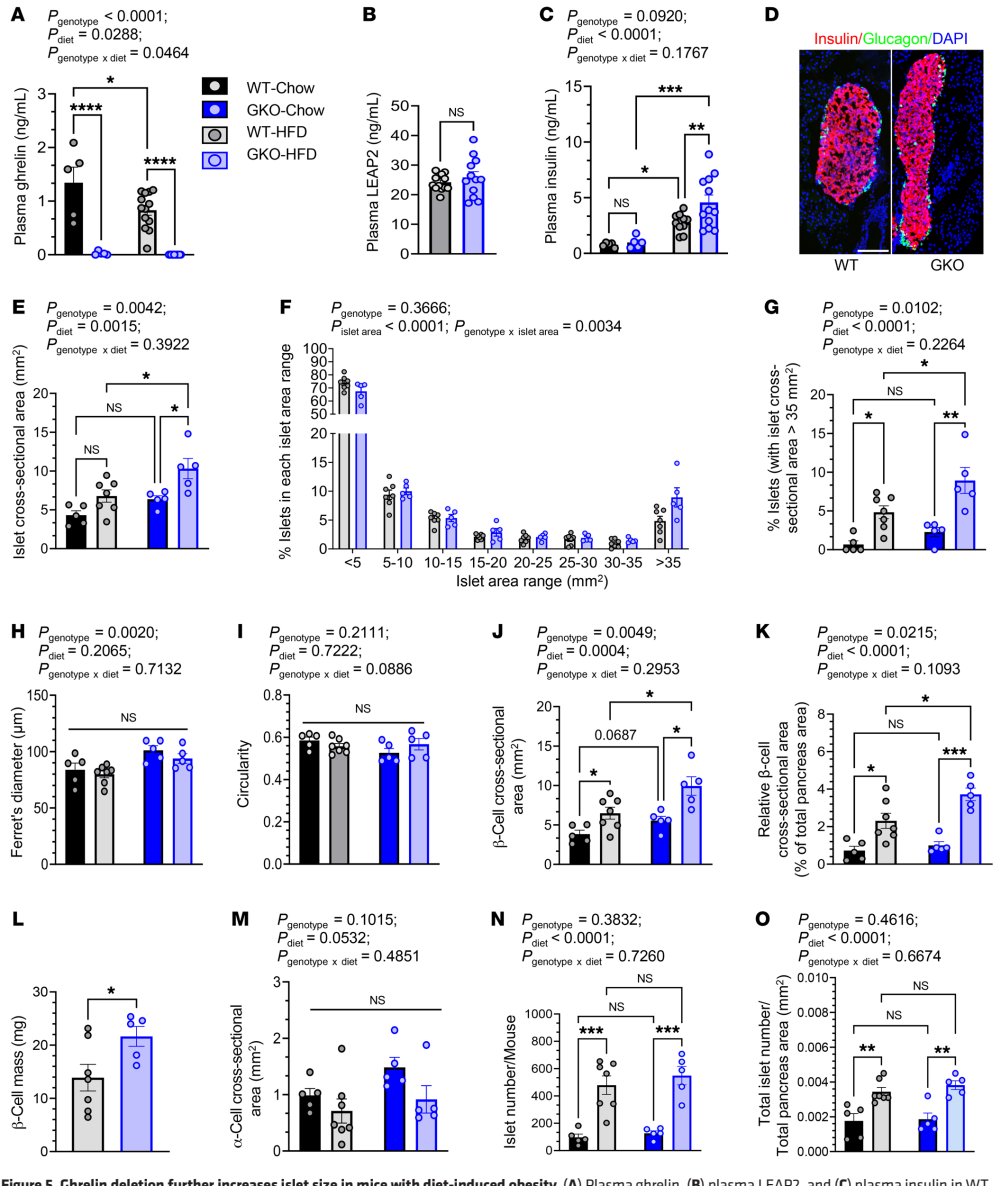
Ghrelin deletion further increases islet size in mice with diet-induced obesity. (**A**) Plasma ghrelin, (**B**) plasma LEAP2, and (**C**) plasma insulin in WT and GKO mice fed standard chow or a HFD. (**D**) Representative islet images from HFD-fed WT and GKO mice. (**E**) Islet cross-sectional area. Scale bar: 100 μm. (**F**) Percentage of islets in each islet cross-sectional area range, in which each range except the last represents a 5 mm^2^ interval. (**G**) Percentage of islets in an islet cross-sectional area range of greater than 35 mm^2^, from WT and GKO mice fed standard chow or a HFD. (**H**) Ferret’s diameter of islets. (**I**) Circularity of islets. (**J**) β Cell cross-sectional area per islet. (**K**) Relative β cell cross-sectional area. (**L**) β Cell mass in WT and GKO mice fed a HFD. (**M**) α Cell cross-sectional area per islet. (**N**) Number of islets (as counted in 4 head-to-tail pancreatic sections per mouse) from WT and GKO mice fed either chow or a HFD. (**O**) Total number of islets per total pancreas area, as counted in 4 head-to-tail pancreatic sections per mouse. *n* = 5–13 mice (**A** and **C**), *n* = 12–13 mice (**B**), *n* = 5–7 mice (**D**–**O**). Data were analyzed by unpaired, 2-tailed Student’s *t* test (**B** and **L**), 2-way repeated-measures ANOVA followed by Bonferroni’s multiple-comparison test (**F**), and 2-way ANOVA followed by Tukey’s multiple-comparison test (**A**, **C**, **E**, **G**, **K**, and **M**–**O**). *P < 0.05, **P < 0.01, ***P < 0.001, and ****P < 0.0001.

In the setting of diet-induced obesity, plasma ghrelin is low and islet size and β cell mass are elevated (38–44). Several processes contribute to diet-induced, obesity-associated ghrelin reduction, including increased engagement of insulin receptors expressed by ghrelin cells, reduced sensitivity of ghrelin cells to the ghrelin secretagogue norepinephrine, increased activation of ghrelin cell–expressed fatty acid receptors, and/or changed numbers of ghrelin cells (39, 45–48). To investigate the contribution of low ghrelin in mediating the islet morphology response to diet-induced obesity, we placed 4-week-old GKO and WT littermate mice on a 60% HFD for 10 weeks. Although GKO and WT mice gained similar amounts of BW (Supplemental Figure 6A), weekly food intake curves diverged, with GKO mice eating approximately 19% less by week 10 compared with WT mice (Supplemental Figure 6B). GKO and WT mice had similar increases in the percentage of fat mass (Supplemental Figure 6, C and D) and similar decreases in the percentage of lean mass (Supplemental Figure 6, E and F). We confirmed the expected obesity-associated drop in plasma ghrelin in 14-week-old ad libitum–fed mice with diet-induced obesity, which had approximately 38% lower levels than 10- to 12-week-old standard chow–fed WT mice (those mice from Figure 1); ghrelin was undetectable in GKO mice regardless of diet (Figure 5A). Although we did not assess plasma LEAP2 in standard chow-fed mice, the levels of this endogenous GHSR antagonist/GHSR inverse agonist (42, 49), which were similar in HFD-fed GKO and WT mice (Figure 5B), were just as high as previously demonstrated in mice after prolonged HFD exposure (50). We also confirmed the expected obesity-associated rise in plasma insulin levels, which were approximately 257% higher in 14-week-old HFD-fed WT mice than in 10- to 12-week-old standard chow–fed WT mice (Figure 5C). Moreover, this rise in insulin was further enhanced by ghrelin deletion, as indicated by approximately 66% higher levels in HFD-fed GKO mice versus HFD-fed WT littermates (Figure 5C).

We analyzed 3,355 islets from 7 HFD-fed WT mice and 2,745 islets from 5 HFD-fed GKO mice (Table 1). Furthermore, the morphology of these islets was compared with that of the standard chow–fed mice reported in Figure 1. The overall structural organization of the islets and their pancreatic distribution was similar in HFD-fed WT and GKO mice (Figure 5D). A HFD and ghrelin deletion both independently increased the mean islet cross-sectional area (Figure 5E), resulting in HFD-fed GKO mouse islets that were approximately 62% larger than those from standard chow–fed GKO mice and approximately 52% higher than those from HFD-fed WT mice. A HFD and ghrelin deletion both independently increased the relative islet cross-sectional area, resulting in values for HFD-fed GKO mouse islets that were approximately 234% higher than those for standard chow–fed GKO mice and approximately 60% higher than those for HFD-fed WT mice (Supplemental Figure 6G). This effect of ghrelin deletion in HFD-fed mice was driven by increases in the percentage of very large islets (>35 mm^2^, ~85% increase, *P_genotype x islet area_* = 0.0034) (Figure 5F). Ghrelin deletion and diet-induced obesity both independently increased the percentage of those islets that were larger than 35 mm^2^, with the greatest percentage being in the GKO mice with diet-induced obesity (Figure 5G). No significant differences in mean Ferret’s diameter or circularity were noted (Figure 5, H and I). A HFD and ghrelin deletion both independently increased the mean β cell cross-sectional area, resulting in an approximately 53% higher value for HFD-fed GKO mice than for HFD-fed WT mice (Figure 5J). Similarly, the relative β cell cross-sectional area and β cell mass were approximately 61% and approximately 56% higher for HFD-fed GKO mice than for HFD-fed WT mice, respectively (Figure 5, K and L). Although diet-induced obesity reduced the mean α cell cross-sectional area (*P* = 0.0532), we found no effect of ghrelin deletion (Figure 5M). Islet number and total islet number per total pancreas area were markedly higher (by 335%–395% and 96%–105%, respectively) in mice with diet-induced obesity, regardless of genotype (Figure 5, N and O).

### Plasma ghrelin negatively correlates with islet size.

**Figure 6:**
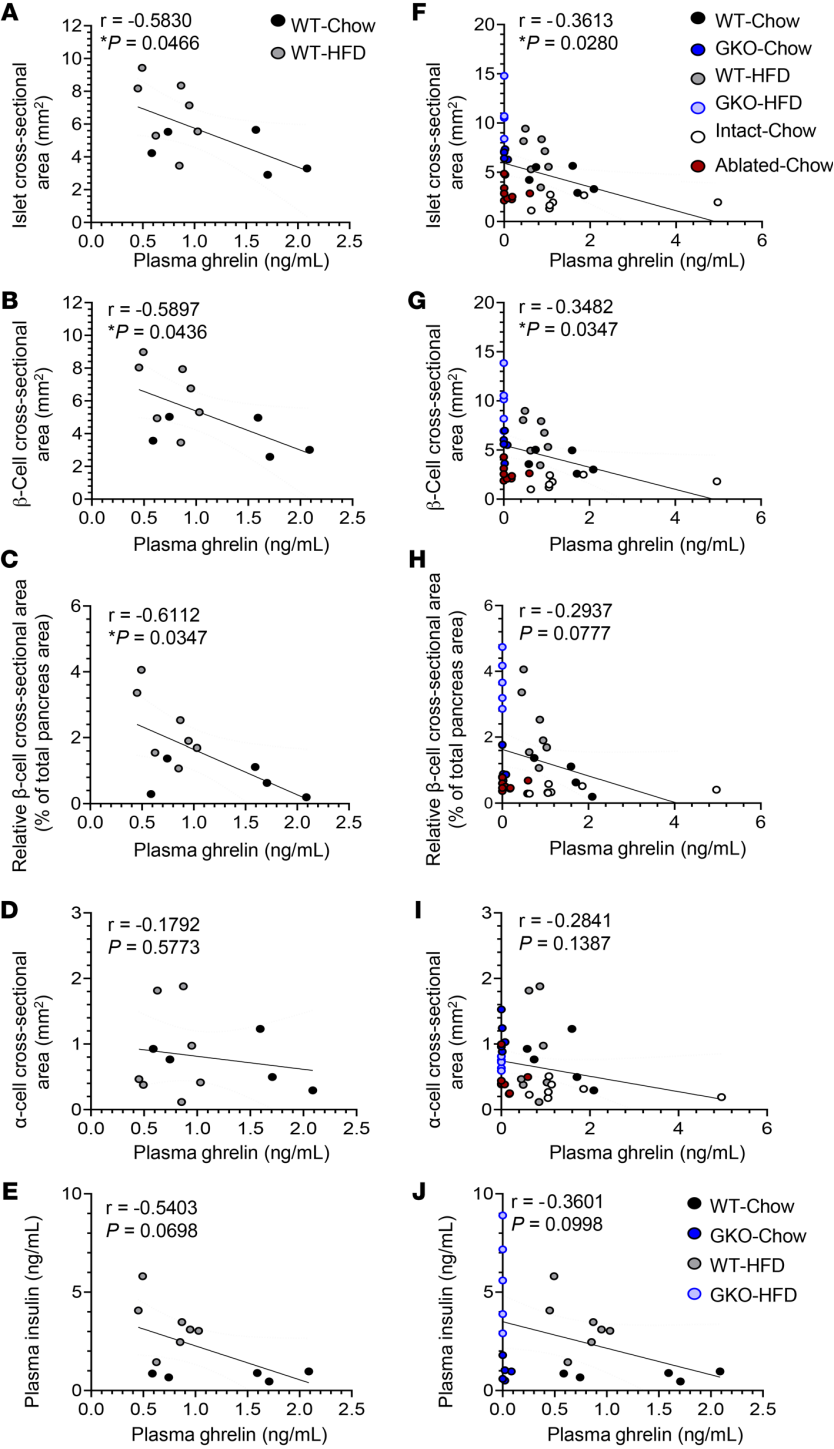
Plasma ghrelin negatively correlates with islet size. Correlations of (**A**) ad libitum–fed plasma ghrelin levels in ad libitum–fed mice with islet cross-sectional area, (**B**) ad libitum–fed plasma ghrelin with β cell cross-sectional area, (**C**) ad libitum–fed plasma ghrelin with relative β cell cross-sectional area, (**D**) ad libitum–fed plasma ghrelin with α cell cross-sectional area, and (**E**) ad libitum–fed plasma ghrelin with ad libitum–fed plasma insulin in WT mice fed standard chow or a HFD. Correlations of (**F**) ad libitum–fed plasma ghrelin with islet cross-sectional area, (**G**) ad libitum–fed plasma ghrelin with β cell cross-sectional area, (**H**) ad libitum–fed plasma ghrelin with relative β cell cross-sectional area, (**I**) ad libitum–fed plasma ghrelin with α cell cross-sectional area, and (**J**) ad libitum–fed plasma ghrelin with ad libitum–fed plasma insulin in WT and GKO mice fed standard chow or a HFD and in mice with intact or ablated ghrelin cells. *n* = 12 mice (**A**–**E**), *n* = 37 mice (**F**–**J**). Data were analyzed by Pearson’s correlation and simple linear regression analysis. Pearson’s correlation coefficient (*r*) and *P* values are indicated in the figure panels.

We reanalyzed the mean islet cross-sectional area, β cell cross-sectional area, relative β cell cross-sectional area, and α cell cross-sectional area (Figure 6, A–D) for the HFD-fed and standard chow–fed adult WT mice (from Figure 1, B, G, and J, and Figure 5, E, J, K, and M) by correlating those values with plasma ghrelin levels in ad libitum–fed mice (referred to hereafter as ad libitum–fed plasma ghrelin levels) (Supplemental Figure 1E and Figure 5A). Islet cross-sectional area, β cell cross-sectional area, and relative β cell cross-sectional area, but not α cell cross-sectional area, were each negatively correlated with plasma ghrelin levels. Furthermore, plasma insulin and ghrelin levels were negatively correlated (*P* = 0.0698; Figure 6E). The negative correlations between islet cross-sectional area, β cell cross-sectional area, relative β cell cross-sectional area, and ad libitum–fed plasma ghrelin levels persisted when the data from HFD-fed and standard chow–fed adult GKO mice and the data from mice with intact and ablated ghrelin cells (from Supplemental Figure 3A and Figure 4, E, I, and P) were included alongside those from WT mice (Figure 6, F–H). The inclusion of those additional cohorts did not lead to a correlation between plasma ghrelin and mean α cell cross-sectional area (Figure 6I). The negative correlation between plasma insulin and ghrelin levels persisted when the data from GKO mice fed standard chow or a HFD (from Supplemental Figure 1, D and E and Figure 5, A and C) were included (*P* = 0.0998; Figure 6J).

### Single-cell transcriptomics profiling of GKO islet cells.

**Figure 7:**
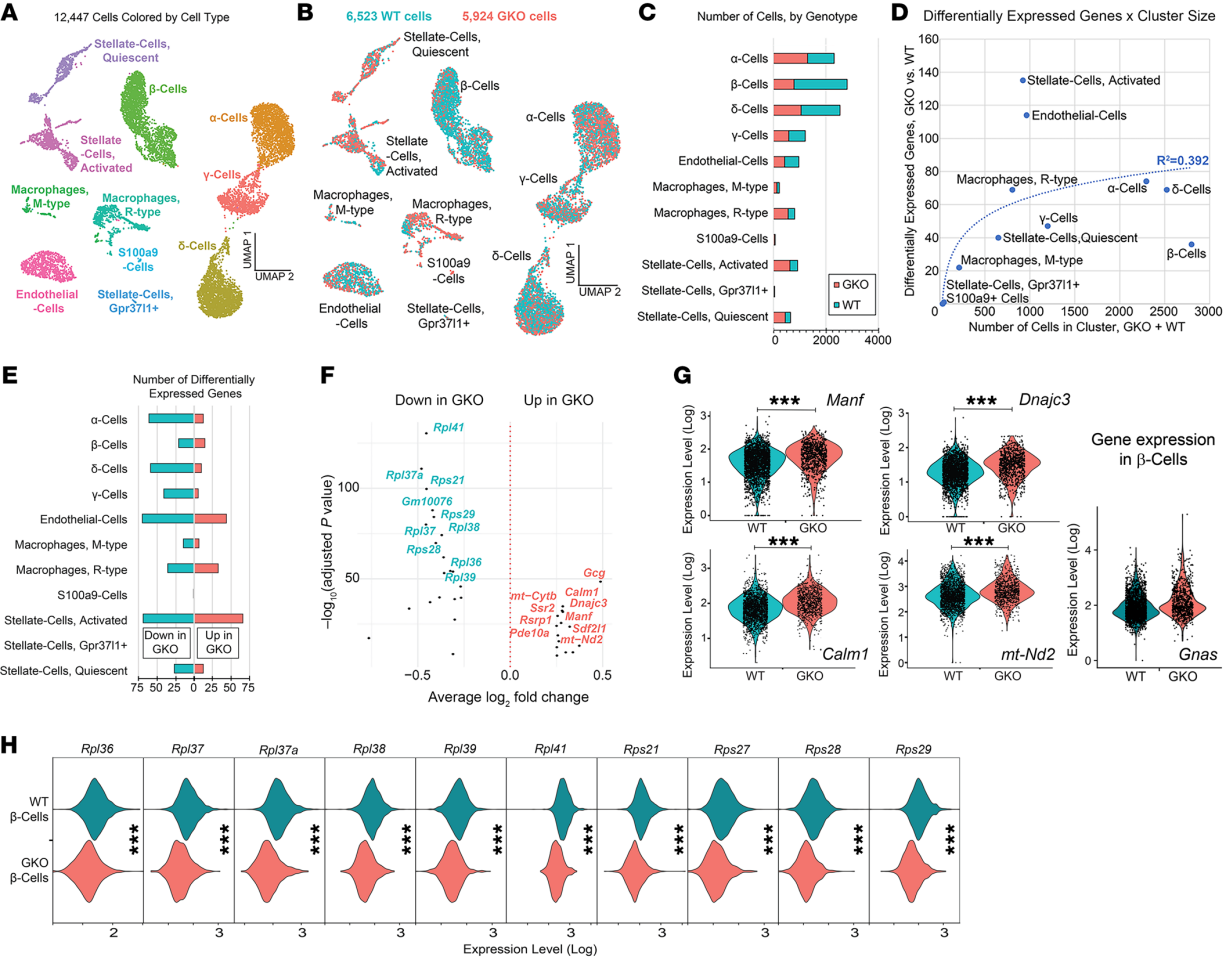
Single-cell transcriptomics profiling of GKO islet cells. (**A**) Uniform manifold approximation and projection (UMAP) plot of singly distributed islet cells (*n* = 12,447 total WT plus GKO cells) highlighting their distribution into 11 distinct cell clusters (each dot represents a single cell). (**B**) UMAP plot and (**C**) bar graph highlighting genotype-dependent differences in the numbers of cells within each cell cluster. (**D**) Number of differentially expressed genes as a function of cluster size for each islet cell cluster. (**E**) Number of upregulated and downregulated genes per cluster resulting from ghrelin deletion. (**F**) Most highly upregulated and downregulated β cell genes resulting from ghrelin deletion. (**G**) Violin plots comparing β cell expression of *Manf*, *Dnajc3*, *Calm1*, *mt-Nd2*, and *Gnas* (each dot represents a single cell expressing the gene of interest, with its corresponding relative expression level indicated on the *y* axis). (**H**) Violin plots illustrating some of the most highly downregulated β cell genes due to ghrelin deletion.

To better understand potential molecular mediators of the increased islet size associated with ghrelin reduction, we undertook a single-cell RNA-Seq analysis of 6,523 and 5,924 islet cells from 8-week-old standard chow–fed WT and GKO littermate mice (*n* = 4 mice/tissue pool; 1 tissue pool/genotype), respectively. Cells with similar transcriptomes were grouped into 13 distinct cell clusters, although ductal cells (marker = *Krt19*) and acinar cells (marker = *Cpb1*) were removed from further analysis after being deemed likely contaminants from surrounding nonislet pancreatic tissue. The remaining 11 distinct islet cell clusters included the 4 traditional islet endocrine cell types: α cells (marker = *Gcg,* which encodes glucagon), β cells (markers = *Ins1* and *Ins2,* which encode insulin), γ cells (marker = *Ppy*, which encodes pancreatic polypeptide), and δ cells (marker = *Sst,* which encodes somatostatin), as well as the following 7 nonendocrine cell types: endothelial cells (markers = *Pecam1* and *Plvap*), activated stellate cells (markers = *Pdgfra*, *Sparc,* and *C3*), quiescent stellate cells (markers = *Pdgfrb, Sparc,* and *Rgs5*), Gpr37I1^+^ stellate cells (markers = *Gpr37I1* and *Sparc*), resident (R) macrophage cells (markers = *Cd86* and *Cx3cr1*), monocyte-derived (M) macrophage cells (markers = *Cd86* and *Ly6c2*), and S100a9^+^ cells (marker = *S100a9*) (Figure 7A). Across genotypes, we detected an average ± SD of 3,230 ± 983 genes per cell, based on an average ± SD of 18,252 ± 14,844 unique transcripts captured per cell, although gene and transcript detection varied by cell type (Supplemental Figure 7). Genotype-dependent differences in the numbers of cells within each cell cluster were noted for all cell clusters, including β cells (Figure 7, B and C). Regarding the latter, we detected 2,006 β cells from WT islets and 791 β cells from GKO islets, which was the opposite of what we expected, given on the histologic analysis, which had revealed an increase in the mean β cell cross-sectional area and β cell numbers per islet in GKO islets versus WT islets (Figure 1, G and H).

**Figure 8:**
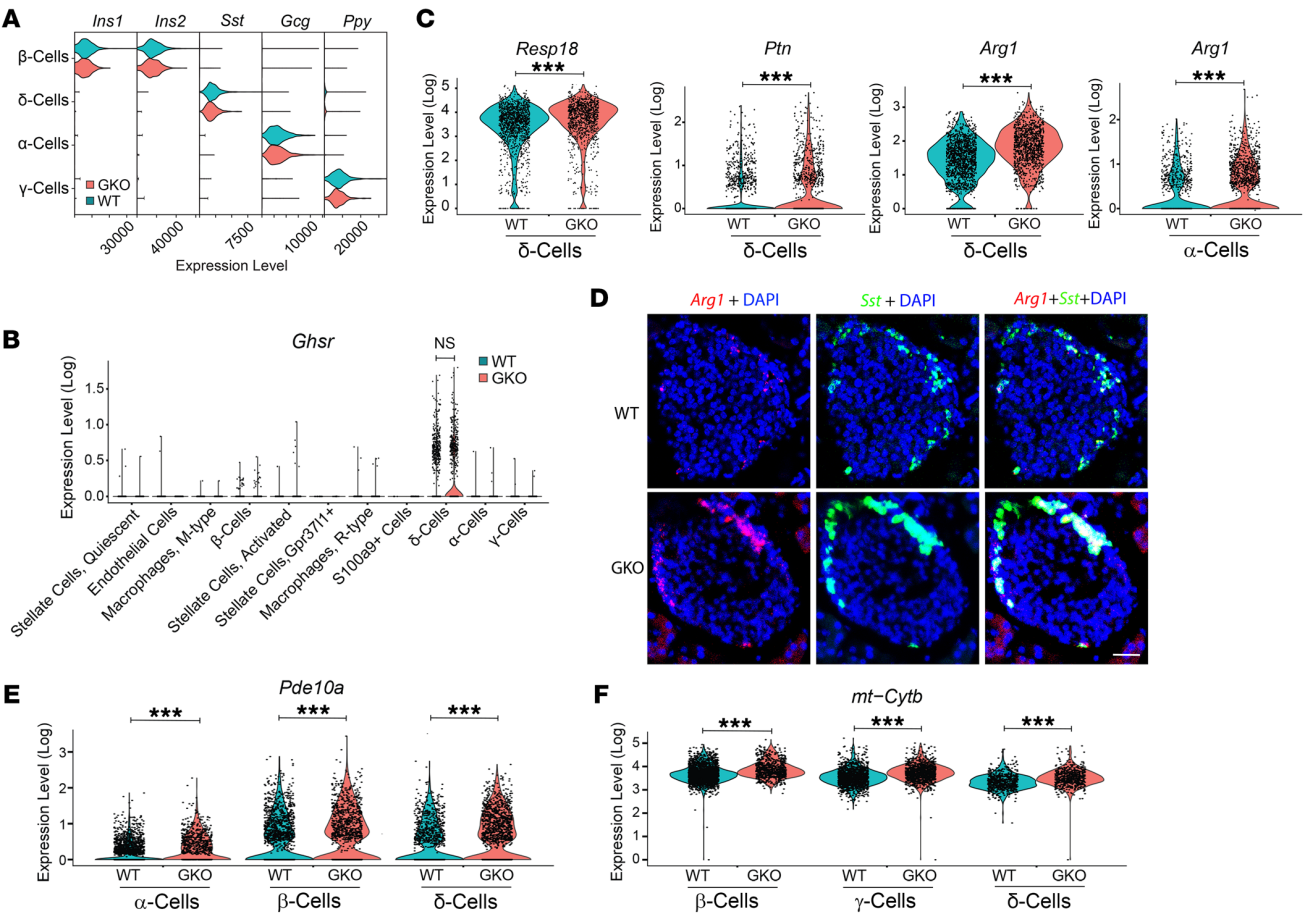
Transcriptional changes in GKO islet cells. (**A**) Violin plots demonstrating expression patterns of islet hormone genes within the cell clusters that they define. (**B**) Violin plots of *Ghsr* expression patterns within WT and GKO islets. (**C**) Violin plots of *Resp18*, *Ptn*, and *Arg1* expression within islet δ cells and *Arg1* expression within islet α cells. (**D**) Representative photomicrographs demonstrating *Arg1* (in red, far left panels) and *Sst* (in green, middle panels) mRNA expression with DAPI staining (in blue) and merged images (right panels) showing colocalized *Arg1* and *Sst* expression (in yellow) within islets from WT (upper panels) and GKO (lower panels) mice. Scale bar: 100 μm. *n* = 4 mice/group. (**E**) Relative expression of *Pde10a* in α cells, β cells, and δ cells from WT and GKO mice. (**F**) Relative expression of *mt-Cytb* in β cells, γ cells, and δ cells from WT and GKO mice.

The 4 cell clusters of endocrine origin represented those with the highest cell populations (Figure 7, A–C) yet were among those with the lowest numbers of differentially expressed genes due to ghrelin deletion per cluster size (Figure 7D and Supplemental Tables 2–5). In contrast, activated stellate cells and endothelial cells contained the highest number of differentially expressed genes per cluster size (Figure 7D and Supplemental Tables 6–12). In most clusters, ghrelin deletion was associated with more downregulated than upregulated gene expression (Figure 7E). An analysis of gene ontology (GO) terms among the differentially expressed genes showed that “cytoplasmic translation” genes were overrepresented in all cell types, suggesting that ghrelin deletion generally alters the translational capacity of islet cells; several other biological processes were also highlighted by this strategy (Supplemental Figure 8). Within β cells, *Manf*, which encodes mesencephalic astrocyte–derived neurotrophic factor, *Dnajc3*, which encodes DnaJ heat shock protein family 40 member C3, *Calm1*, which encodes calmodulin 1, *mt-Nd2*, which encodes the mitochondrially encoded NADH dehydrogenase 2, and *Gnas*, which encodes G-protein α-stimulatory subunit, were among the most highly upregulated genes (Figure 7, F and G). The most downregulated β cell genes included several encoding proteins that govern ribosomal function, many of which were also downregulated in other cell clusters (Figure 7, F and H, and Supplemental Tables 2–8, 10, and 11). Ghrelin deletion did not change the expression of the genes encoding the 4 main islet hormones within the cell clusters that they define (Figure 8A), nor did it alter islet *Ghsr* expression (Figure 8B).

We also focused on differential gene expression within δ cells, in which islet *Ghsr* expression is highest (5, 7, 8) (Figure 8B). *Resp18*, which encodes regulated endocrine-specific protein 18, *Ptn,* which encodes pleiotrophin, and *Arg1*, which encodes arginase 1, were among the most highly upregulated genes in this cluster (Figure 8C). *Arg1* was also highly upregulated in α cells (Figure 8C). Dual-label ISH histochemistry (*n* = 4 mice/genotype) confirmed the high coexpression of *Arg1* and *Sst* and upregulated *Arg1* expression in GKO islets (Figure 8D). Additionally, *Pde10a*, which encodes phosphodiesterase 10A, and *mt-Cytb*, which encodes mitochondrially encoded cytochrome B, were highly upregulated in δ cells as well as in α cells and β cells (*Pde10a*) and in β cells and γ cells (*mt-Cytb*) (Figure 8, E and F).

## Discussion

We demonstrate that in mice, reduced ghrelin — whether by germline genetic deletion of the gene encoding ghrelin, conditional ablation of ghrelin cells in adult mice, or physiological ablation as a result of diet-induced obesity — is associated with increases in mean islet size, percentage of very large islets, and β cell cross-sectional area. In GKO mice, these effects were observed at 4 weeks of age, but not at P0-P2, and became more pronounced at 10–12 weeks of age. Although only assessed in adult mice, the increased β cell cross-sectional area in GKO mice and in mice with ablated ghrelin cells resulted from increased numbers, but not increased sizes, of β cells. We identified reduced β cell apoptosis, but not increased β cell proliferation, as a likely mechanism for the increased β cell numbers in the conditional ghrelin cell ablation model. GKO juveniles and adults also had an increase in α cell cross-sectional area.

To our knowledge, ghrelin deletion alone has not been shown to increase islet size or β cell mass. Only when ghrelin deletion was combined with leptin deficiency was the mean islet area increased over that induced by leptin deficiency alone (27). Another highly rigorous study demonstrated no change in islet size at embryonic stages in GKO mice (23), which likely explains our observations of normal-sized islets in P0–P2 GKO mice. However, the exact reasons for the apparent discrepancies between our results and those of other studies, which reported either no effects of ghrelin or GHSR deletion on islet size in adult WT and/or leptin-deficient mice, reduced insulin IR area per islet as a result of pharmacologic GHSR antagonism, or an attenuated decrease in total islet area per total pancreatic area and in β cell proliferation as a result of ghrelin administration to BB rats are unclear (10, 24–31). We can only surmise that differences in the rigor or methodology used to assess islet morphology and differences in the metabolic settings to which the mice were exposed might be contributing factors.

It is notable that the observed effects of reducing ghrelin on islet size and β cell mass were observed not only in juvenile and adult mice upon germline deletion of the ghrelin gene but also upon conditional ablation of ghrelin cells in adult mice, with its resulting marked and rapid decline in plasma ghrelin levels. Thus, it appears that the absence of ghrelin at some critical early developmental stage of the mice was not required for the increased islet size observed in adulthood. Rather, inducing a reduction in ghrelin in older mice (here, at 6 weeks of age) also resulted in enlarged islets and β cell mass.

Such a relatively marked and rapid effect of conditional ghrelin reduction could potentially be harnessed to increase β cell mass as a treatment for T1DM. One could envision a therapeutic strategy whereby neutralizing ghrelin, such as has already been achieved using an anti-ghrelin RNA spiegelmer (51), or decreasing GHSR signaling in other ways (52) could be used to increase β cell numbers within donor islets, optimize the proliferation of cultured β cell lines, and/or favor the expansion of β cells within islet organoids (pseudoislets) (53, 54) prior to or following β cell transplantation. Decreasing GHSR signaling in patients who have undergone islet cell transplantation would, presumably, also favorably affect glycemic control in other ways, for instance, by enhancing insulin sensitivity, directly and indirectly promoting insulin secretion, and increasing islet vascularity, all of which have previously been documented (6, 9, 14). It remains to be seen whether islets or β cells from low-ghrelin environments would also exhibit improved survival following transplantation, as has been shown, for instance, with enlarged islets from transgenic mice that overexpress hepatocyte growth factor in β cells (55).

Additionally, neutralizing ghrelin might show efficacy as a T1DM prevention strategy and in the management of T2DM. For instance, in the nonobese diabetic (NOD) mouse model, enhancing β cell proliferation prior to islet infiltration by immune cells alters the immunogenic identity of β cells, protecting the mice from developing T1DM (56). One wonders if enhancing β cell numbers by neutralizing ghrelin would have the same effect as genetically deleting hepatic insulin receptors, as was done in the latter study (56). Also, during the pathogenesis of T2DM, longstanding insulin resistance causes β cells to become dysfunctional and/or dormant, eventually leading to the deterioration of glycemic control (57). This results, at least in part, from inactivation of key β cell transcriptional complexes (57). Neutralizing ghrelin could conceivably serve as a novel means to replenish β cells in patients with T2DM.

Another relevant discussion topic is the potential contribution of lowered ghrelin to the increase in islet size in diet-induced obesity. As expected (38, 42–44), we observed reduced plasma ghrelin levels in WT mice that developed diet-induced obesity. Furthermore, we showed a negative correlation between islet cross-sectional area or β cell cross-sectional area and plasma ghrelin levels when the data from HFD-fed and standard chow–fed WT mice were pooled. That said, HFD-fed GKO mice also developed increased islet sizes, such that their mean islet cross-sectional area and their percentage of very large islets were even greater than those observed in HFD-fed WT mice. Thus, although the correlation data coupled with the GKO and ghrelin cell ablation data suggest that the reduced ghrelin in diet-induced obesity contributed to islet enlargement, the presence of larger islets in HFD-fed GKO mice suggests that diet-induced obesity–associated islet enlargement was not solely mediated by reduced ghrelin. Rather, other factors must have been at play to allow GKO mice to have an increase in islet size when fed a HFD. A previous study suggested that, in the setting of diet-induced obesity, as well as during pregnancy and in *db/db* mice (which lack functional leptin receptors), downregulated expression of the islet microRNA miR-338-3p contributes to increased islet size (41). These factors might involve changes to GHSR signaling present in the setting of diet-induced obesity besides reductions in circulating ghrelin. For instance, in diet-induced obesity, not only is ghrelin reduced, but LEAP2 is elevated (42, 50, 52). Here, we demonstrated equivalent plasma LEAP2 levels in HFD-fed WT and GKO mice; these levels were higher than those observed in standard chow–fed WT mice (50). This elevated LEAP2 could reduce GHSR signaling even further than that associated with ghrelin deletion alone, thereby potentially contributing to the large-sized islets seen in HFD-fed GKO mice.

Finally, the transcriptomics data reveal clues regarding potential mechanisms by which ghrelin reduction increases islet size. The first clue can be found in those genes with the most upregulated expression in β cells as a result of ghrelin deletion. Involvement of those genes makes sense, given the increased β cell cross-sectional area and β cell numbers observed histologically in GKO mice and mice with ablation of ghrelin cells. Indeed, reducing ghrelin may directly affect β cells, given the fact that they express GHSR (5) (Figure 7J). *Manf* is of interest because its gene product is known to protect β cells from hyperglycemia-induced ER stress and to activate the unfolded protein response, which in turn helps minimize β cell apoptosis and promotes β cell proliferation (58). *Manf* is required for postnatal expansion of β cell mass and for β cell maintenance in adult mice, whereas its deletion from adult β cells in mice reduces β cell mass as a result of increased β cell apoptosis and decreased β cell proliferation, leading to diabetes (58). *Dnajc3* is of interest because its gene product plays a protective role in the ER stress response, its deletion in mice increases β cell apoptosis and reduces β cell numbers leading to hypoinsulinemia and hyperglycemia, and its loss of function in humans leads to a syndrome which includes juvenile-onset diabetes (59, 60). *Calm1*, *Pde10a*, and *Gnas* are of interest because their gene products serve as important intracellular signaling molecules. Genetic deletion of *Gnas* in β cells reduces both the expression of key β cell identity and maturation genes in postnatal β cells and β cell mass, the latter resulting from decreased β cell proliferation without changes in β cell apoptosis (61). Although we did not identify increased β cell proliferation following ghrelin cell ablation, we are mindful that the experimental time points used here to detect proliferation changes may have been suboptimal. Specifically, while the reduction of plasma ghrelin in our ghrelin cell ablation model was near-maximal 24 hours after DTX administration (34), we examined BrdU incorporation at a time prior to detectable increases in β cell numbers (11 days following DTX), and we examined Ki67 IR 4 weeks after DTX. Thus, we may have missed the window during which we could have best captured increased β cell proliferation, if present. Nonetheless, as some of the transcriptomics data predict, we observed decreased β cell apoptosis in islets following ghrelin cell ablation. Finally, *mt-Nd2* and *mt-Cytb* are of interest because they encode key components of mitochondrial respiration. A SNP in *mt-Nd2* has been associated with autoimmune β cell death in NOD mice and humans (62).

A second clue can be found embedded in those genes with the most upregulated expression in δ cells as a result of ghrelin deletion, especially given the high *Ghsr* expression within δ cells in both mice and humans (Figure 7J), the ghrelin’s known actions of direct stimulation of somatostatin release from δ cells, and somatostatin’s known actions of restricting β cell proliferation (5, 7, 8, 63). The *Resp18* gene product is of interest because of its high expression within several peptide-producing neuroendocrine cell types and its potential role in hormone release, as suggested by its localization to the ER lumen and within secretory granules of various islet and other neuroendocrine cell types (64, 65). Resp18 is upregulated in islets by exposure to high glucose and in islets of diabetic NOD mice, and it shares a partial sequence homology with IA-2, which is a dense-core secretory vesicle protein that helps facilitate insulin secretion (64). The *Ptn* gene product pleiotrophin is a secreted heparin-binding cytokine that is of interest because of its capacity to promote angiogenesis, act as a protooncogene, stimulate cell differentiation, and induce proliferation of several cell types, including β cell–derived cell lines (66, 67). Intense pleiotrophin expression is observed during β cell regeneration following STZ-induced β cell depletion in mice (67). *Arg1* is of interest because of the actions of its gene product to convert arginine to ornithine, which subsequently is transformed to the cell proliferation–promoting and cell differentiation–promoting polyamines putrescine, spermidine, and spermine (68, 69). In turn, an aminobutyl group on spermidine is used by the enzyme deoxyhypusine synthase to modify and thereby activate eukaryotic translation initiation factor 5A (eIF5A), which is essential for translation of mRNAs encoding proteins with proline repeats, many of which regulate fundamental aspects of growth and development (68, 69). In mice, β cell–selective deletion of deoxyhypusine synthase, which is normally present in higher amounts in HFD-fed WT mice, attenuates HFD-induced β cell proliferation, resulting in smaller islets and the development of diabetes (70). Inhibiting conversion of ornithine to polyamines has a similar effect to stunt β cell proliferation in HFD-fed WT mice (70). It is as yet uncertain whether an increased supply of polyamines, as was predicted here in GKO δ cells and α cells due to *Arg1* upregulation, might find their way to neighboring β cells, where they could stimulate eIF5A-dependent cell-proliferative responses.

We encourage future studies that take advantage of these transcriptomics data to define the molecular mechanisms by which reducing ghrelin decreases β cell apoptosis and increases islet size and that identify the specific organ(s) and cell types directly engaged by ghrelin to affect these phenomena. Also, we encourage future studies that confirm the translatability of these findings to human islets, which have many similarities to mouse islets, but differences as well (71), including the disappearance of ghrelin-producing ε cells from adult mouse islets versus their persistence (albeit at a much lower occupancy than in the fetal period) in human islets (18–21). Indeed, we wonder if reducing ghrelin in adult islets would have an even more profound effect on islet size in humans than in mice, given the usual persistence of a local source of ghrelin production in adult human islets.

Although the histologic evidence for increased mean β cell cross-sectional area and β cell numbers in GKO mice is clear, β cells comprised a smaller proportion of the total population of WT and GKO mouse islet cells analyzed by single-cell RNA-Seq. This is despite the fact that a larger number of GKO mouse islet cells were submitted for FACS than were WT mouse islet cells in the step prior to transcriptomics analysis, suggesting a possible greater fragility of GKO β cells during cell handling. We must take into account that these differential gene expression data from β cells reflect those β cells that survived the FACS. Also of note, our single-cell RNA-Seq analysis included 12,447 islet cells from pooled samples obtained from 4 WT and 4 GKO mice, which is similar to the number of pancreatic islet cells in some atlases (72, 73) but less than in others (74, 75). Our study was sufficiently powered to detect the major islet cell types and their top differentially expressed genes but not for comprehensive characterization of all islet cell types and genes. Further work is needed, for instance, to define how ghrelin gene deletion affected the rare cell types in our data set (S100a9^+^ cells and Gpr37l1^+^ stellate cells) and to detect genes more subtly changed by reducing ghrelin levels in the major cell types. Another limitation of our study is that the lack of biological replicates precluded pseudobulk analysis, which was recently recommended for detecting differentially expressed genes in single-cell RNA-Seq data (76). Furthermore, conventional methods for analyzing differential expression in single-cell RNA-Seq data, such as the Wilcoxon rank-sum test used here, have a high FDR. Thus, to increase rigor, we used 2 different statistical tests for differential expression: the Wilcoxon test and the model-based analysis of single-cell transcriptomics (MAST) test (77), and report only genes that differed to a statistically significant extent in both tests.

In summary, we demonstrate that reducing ghrelin in mice — as achieved by germline ghrelin deletion or conditional ghrelin cell ablation in adults — alone increased islet size and β cell numbers, at least in part due to decreased β cell apoptosis. Our data also suggest that physiological reduction of ghrelin via chronic HFD feeding contributed to diet-induced obesity–associated islet enlargement. Additionally, transcriptomics analysis identified several potential mediators of these islet morphologic effects of ghrelin deletion. Further study of these effects of ghrelin reduction on islet morphology, which the ghrelin cell ablation studies suggest can be rather rapidly and markedly induced in adults, might prove useful in the design of new therapeutic approaches to T1DM and T2DM.

## Methods

### Mouse studies.

Except as noted, mice had ad libitum access to standard chow diet (2916 Teklad Global 16% Protein Rodent Diet, Envigo) and water and were group housed (for histological studies) or individually housed (beginning 1 week prior to physiological studies) at room temperature (21.5°C–22.5°C) in a 12-hour light/12-hour dark cycle. GKO and WT littermate mice were generated by crossing GKO (line GKO1) heterozygotes on a C57BL/6N background, as previously reported (9, 10, 78). Additional details are provided in Supplemental Methods.

### Effects of diet-induced obesity.

Individually house mice were provided ad libitum access to a 60% HFD (D12492, Research Diets) for 10 weeks beginning at 4 weeks of age. BW was measured biweekly, and body composition was measured 1 day before and 10 weeks after starting the HFD using an EchoMRI-100 apparatus (EchoMRI). One day after the final body composition measurement, blood was collected from nicked tails for hormone analysis, and mice were perfused (see Supplemental Methods for details on perfusion and tissue processing).

### Immunohistochemistry.

Immunohistochemical analysis was performed as described previously (5, 79) (see Supplemental Methods for details).

### Islet morphology.

All islets within a series of four 8 μm thick head-to-tail pancreas sections, separated from each other by at least 50 μm, were studied by first imaging each section in its entirety using the ×20 objective of a Zeiss Axio Scan.Z1 Slide Scanner coupled with Zen Lite 2.3 (Zeiss Research Microscopy) and then by extracting individual 8-bit RGB images of each islet. These images were processed and islet morphology was characterized by an investigator blinded to genotype and treatment. Islets were defined by the presence of at least 1 insulin IR, DAPI-stained cell, as previously suggested (80, 81), and as having a cross-sectional area greater than 50 μm^2^. Regions containing insulin IR without DAPI staining were excluded from analysis, as were regions containing only glucagon IR or somatostatin IR with or without DAPI staining and potential islets localized to the nonfocal plane (which appeared blurred). Regions with a few closely approximated but not contiguous clusters of 1 or a few insulin IR DAPI-stained cells were difficult to assign as either a single islet or a group of tiny islets, and were thus excluded from analysis. Images with a suboptimal signal/noise ratio were run through a newly-designed Python program (Supplemental Methods and Supplemental Table 13) to re-scale the brightness of the pixels in the image. Afterwards, the following morphological parameters were determined by analyzing each islet image with a set of newly-designed programs that interface with Fiji Is Just ImageJ software (NIH; http:/rsbweb.nih.gov/ij/; https://imagej.net/Fiji/downloads) (Supplemental Methods and Supplemental Table 14): islet cross-sectional area, Ferret’s diameter, circularity (4 πa/p^2^; where a = area and p = length of the perimeter), β cell cross-sectional area per islet, α cell cross-sectional area per islet, β cell count per islet, and β cell size (calculated by dividing the β cell cross-sectional area within an islet by the number of β cells in that islet; ref. 82). For some experiments, slides from an adjacent series were stained for both insulin IR and somatostatin IR and then processed as described above to determine the δ cell cross-sectional area per islet. The accuracy of these programs was tested by statistically comparing the islet, β cell, and α cell cross-sectional areas, circularity, and Ferret’s diameter determined by the programs with those determined manually for 50 random islets of varying sizes from the studies performed on GKO and ablated mice. Manual determination was done using the computer mousepad together with ImageJ software to trace the perimeter of each islet, after which the above measurements were made with the assistance of the ImageJ “Analyze” tool. The semiautomated and manual strategies both showed similar results, thus validating the programs (Supplemental Table 15).

The relative islet cross-sectional area (or rather, % of the total pancreas area comprised of islet tissue) was calculated by multiplying the total islet cross-sectional area within 4 pancreatic sections by 100 and dividing that number by the total area of those 4 pancreatic sections (83). The relative β cell cross-sectional area (or rather, the percentage of the total pancreas area composed of β cells) was calculated by multiplying the total β cell cross-sectional area within 4 pancreatic sections by 100 and dividing that by the total area of those 4 pancreatic sections (83). β Cell mass was calculated by multiplying the total β cell cross-sectional area within 4 pancreatic sections/total pancreas area of those 4 sections by the pancreas plus spleen weight. (Although β cell mass should be calculated by multiplying the relative β cell cross-sectional area by pancreas weight, we weighed the pancreas together with the spleen and do not have the pancreas weight alone.) The total islet number within 4 pancreatic sections per total pancreas area (in mm^2^) of those 4 sections also was calculated.

### β Cell apoptosis and proliferation assays.

Pancreatic sections from a subset of the 10-week-old intact versus ablated mice (4 weeks after DTX administration to 6-week-old mice) were stained for cleaved caspase-3 plus insulin or for Ki67 plus insulin (Supplemental Methods). β Cell proliferation also was examined using a BrdU incorporation method (84). Briefly, a separate cohort of mice received BrdU (MilliporeSigma, catalog B9285; 50 mg/kg, i.p., prepared in saline) for 4 consecutive days, 1 week after DTX injection (which mice received at 6 weeks of age). Four hours after the fourth BrdU injection, mice were perfused, and pancreases were collected and processed (see Supplemental Methods).

### Glucose-stimulated insulin secretion studies.

Following an overnight fast, blood glucose was measured from nicked tails of 9- to 10-week-old mice between 8:00 am and 9:00 am (*t* = 0 min). d-Glucose (2 g/kg BW prepared in water; MilliporeSigma) was administered by oral gavage at *t* = 0 minutes. Blood glucose was measured from nicked tails 15 and 30 minutes after gavage. Also, blood samples (~15 μL) were taken from nicked tails at *t* = 0, 15, and 30 minutes for subsequent plasma insulin determination, as described previously (10).

### Pancreatic insulin content.

Pancreases from overnight-fasted 9- to 11-week-old mice that had been administered d-glucose (2 g/kg BW) by oral gavage and then decapitated 30 minutes later were processed for pancreatic insulin content following a published protocol (85) (see also Supplemental Methods).

### Blood collection and hormone analysis.

Blood samples collected into ice-cold, EDTA-coated microtubes containing either *p*-hydroxymercuribenzoic acid (final concentration, 1 mM; MilliporeSigma; for ghrelin measurement), aprotinin (final concentration, 250 KIU/mL; MilliporeSigma; for LEAP2 measurement), or nothing else (for insulin measurement) were processed as described in Supplemental Methods.

### Islet isolation and single-cell dispersion.

Islets were isolated from 8-week-old ad libitum–fed GKO and WT mice by collagenase digestion, as previously described (86) (see also Supplemental Methods). Islets from 4 mice were pooled by genotype, with 1 pool of islets representing each genotype.

### FACS.

Islet cells were subjected to FACS analysis using a MOFLO high-speed cell sorter (Beckman Coulter) at the UT Southwestern Flow Cytometry Multi-User Core Facility to isolate enriched populations of singly suspended live islet cells away from dead cells and nonsinglet cells (Supplemental Methods).

### Single-cell RNA-Seq.

The FACS-separated, singly suspended live islet cells from WT (1.1 × 10^5^ cells) and GKO (1.0 × 10^5^ cells) mice were centrifuged (200 rpm, 4°C for 2 min), resuspended in 60 μL fresh RPMI-1640 containing 10% FBS, and submitted to the UT Southwestern Next Generation and Sanger Sequencing Core for further processing (see Supplemental Methods).

### GO overrepresentation analysis.

GO analysis was performed using the WEB-based Gene SeT AnaLysis (WebGestalt) Toolkit (87) (see Supplemental Methods).

### ISH histochemistry.

Ten week-old GKO and WT littermate mice were perfused, and pancreata with attached spleens were removed, processed as described above, and then cut with a cryostat into 14 μm thick sections that were mounted onto slides. Further processing was done as described in Supplemental Methods.

### Statistics.

Data are presented as the mean ± SEM. Two-tailed statistical analysis and graph preparations were performed using GraphPad Prism 9.0.2 (GraphPad Software). A Student’s *t* test, 2-way ANOVA, or repeated-measures 2-way ANOVA, followed by Bonferroni’s or Tukey’s multiple-comparison test was used. Data with unequal variance were log transformed prior to analyses. The strength of the linear relationship between 2 sets of variables was compared by Pearson’s correlation coefficient. Outliers, if any, were removed using Grubb’s test. *P* values of less than 0.05 were considered statistically significant, and *P* values of 0.05 or higher or of less than 0.1 were considered evidence of a statistical trend.

### Study approval.

All experiments were performed using male mice and were approved by the IACUC of UT Southwestern Medical Center (Dallas, Texas, USA).

### Data availability.

The RNA-Seq data sets are available in the NCBI’s Gene Expression Omnibus (GEO) database (GEO GSE244390). The analytic program codes are provided in Supplemental Tables 13 and 14. Values for all data points in the figures can be found in the Supplemental Supporting Data Values file.

## Author contributions

DG and AWB contributed equally and are co–first authors. The order of the co–first authors’ names reflects person-hours and contributions to the writing of the manuscript, preparation of figures, and study design. DG co-designed the study, performed the experiments, helped process the pancreas images, analyzed data (except the transcriptomics data), constructed figures (except the transcriptomics figures), and co-wrote the manuscript. AWB designed and wrote the codes to assess islet morphology, helped process the pancreas images, performed the blinded analyses of the processed pancreas images, and co-wrote the manuscript. DCS analyzed transcriptomics data and constructed the transcriptomics figures with JNC. KS, SV, OS, SP, SBO, SOL, NPM, and CPR assisted DG with performing the experiments. JNC supervised and secured funding for transcriptomics data analyses and co-wrote the manuscript. JMZ co-designed the study, co-wrote the manuscript, and secured funding for and supervised the project. All authors approved the final version of the manuscript.

## Supplementary Material

Supplemental data

Supporting data values

## Figures and Tables

**Table 1:**
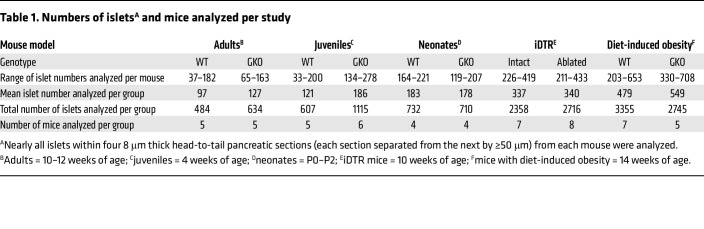
Numbers of islets^A^ and mice analyzed per study

## Version 1 12/15/2023

Electronic publication
