# Ghrelin deletion and conditional ghrelin cell ablation increase pancreatic islet size in mice

**DOI:** 10.1172/JCI182168

**Published:** 2024-05-15

**Authors:** Deepali Gupta, Avi W. Burstein, Dana C. Schwalbe, Kripa Shankar, Salil Varshney, Omprakash Singh, Subhojit Paul, Sean B. Ogden, Sherri Osborne-Lawrence, Nathan P. Metzger, Corine P. Richard, John N. Campbell, Jeffrey M. Zigman

Original citation: *J Clin Invest*. 2023;133(24):e169349. https://doi.org/10.1172/JCI169349

Citation for this corrigendum: *J Clin Invest*. 2024;134(11):e182168. https://doi.org/10.1172/JCI182168

The authors recently became aware that the units of measurement used for islet, β cell, α cell, and δ cell cross-sectional area and total pancreas area in Figures 1B, 1C, 1E, 1G, 1J, 2B, 2C, 2F, 2G, 2J, 2K, 2N, 2O, 3A, 3B, 3C, 4E, 4F, 4I, 4P, 4Q, 4R, 5E, 5F, 5G, 5J, 5M, 5O, 6A, 6B, 6D, 6F, 6G, and 6I were incorrect. The correct unit of measurement is µm^2^ × 10^3^. In addition, the units of measurement reported for β cell size in Figures 1I and 4M were incorrect. The correct unit is μm^2^. The authors have stated that the quantification of data and statistical analyses performed are unaffected, and the conclusions drawn in the paper remain unaltered. The HTML and PDF version of the article and the Supporting Data Values file have been updated with the correct units of measurement.

The authors regret these errors.

## Version 1 05/15/2024

Electronic publication

