# Supplementary material for: Ghrelin deletion and conditional ghrelin cell ablation increase pancreatic islet size in mice
Source: J Clin Invest. 2023 Dec 15;133(24):e169349. doi: 10.1172/JCI169349 (PMC10721155; doi:10.1172/JCI169349)
Supplement: Supplemental data [file jci-133-169349-s077.pdf]

Supplemental Methods

Mouse studies. Except for the mice studied at P0-P1, mice were weaned at 3 weeks-of-age. Mice with conditional ablation of ghrelin cells and control littermates were generated by crossing mice with inducible (Cre recombinase-mediated) expression of diphtheria toxin (DTX) receptor (iDTR) [C57BL/6-Gt(ROSA)26Sortm1(HBEGF)Awai/J; strain #007900, Jackson Laboratory, Bar Harbor, ME] (1) to ghrelin-Cre mice (2). Resulting progeny carrying an iDTR gene ± one copy of the ghrelin-Cre transgene were administered DTX (10 ng/g BW i.p.; Sigma-Aldrich, St. Louis, MO), resulting in mice with ablated ghrelin cells ("Ablated"; those carrying ghrelin-Cre) and control mice with intact ghrelin cells ("Intact"; those without ghrelin-Cre).

Perfusion and tissue processing. Juvenile and adult mice were anesthetized with chloral hydrate (700 mg/kg BW, i.p.) and then were perfused transcardially with PBS, pH 7.0 followed by 10% neutral buffered formalin. Pancreata with attached spleens and stomachs were removed, stored in formalin overnight at 4°C, immersed in graded (5%, 10%, 18%, and 30%) sucrose solutions in PBS for 24 h each at 4°C, and embedded in Tissue-Tek® OCT compound (Sakura Finetek, Torrance, CA). Eight series of four 8 µm-thick pancreatic sections were cut on a cryostat at 50 µm intervals. Eight µm-thick sections from the stomach (two each from the antrum and corpus, each separated by at least 150 µm) were cut on a cryostat. Sections were mounted on SuperFrost Plus glass slides, air dried, and stored at −80°C until further processing. P0-P2 pups were decapitated, after which their pancreata with attached spleens were removed, placed in 10% neutral buffered formalin for 24 h at 4°C, and processed as above.

Immunohistochemistry. Slides were washed three times with PBS, incubated with 3% normal donkey serum for 1 h at room temperature, and incubated overnight with either goat polyclonal anti-ghrelin (Santa Cruz Biotechnology, Dallas, TX; sc-10368; diluted 1:1000)], guinea pig anti-Insulin (DakoCytomation, Carpinteria, CA; A0564; diluted 1:300) + rabbit anti-Glucagon (Cell Signaling; 2760; diluted 1:200), or guinea pig anti-insulin + rabbit anti-somatostatin (Immunostar, Hudson, WI; 20067; diluted 1:1000)]. Then, the slides were washed three times with PBS and incubated for 1 hr with secondary antibodies diluted 1:500: Alexa Fluor 594® donkey anti-goat (Invitrogen, Life Technology Corporations, Eugene, OR; A11058) or Alexa Fluor 594® goat anti-guinea pig (A11076) + Alexa Fluor 488® donkey anti-rabbit (A32790). Next, slides were washed with PBS and coverslipped with Vectashield-DAPI mounting medium (Vector Laboratories, Burlingame, CA).

Details of program codes written to assess islet morphology. Images of individual islets were extracted as 8-bit RGB images at 50% magnification from the images of pancreatic sections. Afterwards, for those images with sub-optimal signal/noise ratio (which was only observed in the green channel of occasional islets stained for glucagon), a *de novo* Python program (Supplemental Table 13), which incorporates several previously-described programming libraries[1-4], was used to remove noise. This program uses statistical information about pixel brightness throughout the image to generate a logistic curve to adaptively re-scale the brightness of every pixel in the image. Specifically, the program inputs pixel brightness values to the function $f(x) = \frac{x}{1+e^{-(\frac{4.5}{\sigma}*(x-2\bar{x}))}}$ (where 'x' = a pixel's brightness, '$\bar{x}$' = the median brightness, and 'σ' = the standard deviation of pixel brightness in the islet image). The median value primarily translates the function horizontally over brightness values, while the standard deviation affects the horizontal stretch, or compression, of the curve. The behavior of $f(x)$ can be represented by:

$$g(x) = \begin{cases} x < 2\bar{x} - \frac{3\sigma}{4},\ 0 \\ 2\bar{x} - \frac{3\sigma}{4} \leq x \leq 2\bar{x} + \frac{3\sigma}{4},\ f(x). \\ 2\bar{x} + \frac{3\sigma}{4} < x,\ 1 \end{cases}$$

As this representation indicates, brightness values within ~0.75 standard deviations of the median are scaled by the logistic function above; values farther than -0.75 standard deviations from the median are scaled to 0, while values farther than +0.75 standard deviations are left unchanged.

To automate islet morphologic analysis, while also removing potential subjective bias, we wrote five Jython programs which interface with (Fiji is Just) ImageJ software. These programs control a series of ImageJ 'macros' for image processing and analysis. The first step of image processing was to set the scale value for each pixel to 1.18 µm. The first program measured islet cross-sectional area by splitting the composite image into red, green, and blue channels, merging the red and green channels (and removing the blue channel), and creating a monochromatic (8-bit gray scale) image. Subsequently, a binary mask was applied at a threshold of 20, which is a value that was obtained through trial-and-error. This provided a constant and consistent thresholding of all images and ensured that only the target signal from the islet, and not from the surrounding exocrine pancreas, was analyzed. This step ensured that even islets with low brightness were analyzed. Finally, the program drew a perimeter around the masked object and measured the entire area within the perimeter. A similar process was used in the next three programs to measure β-cell cross-sectional area, α-cell cross-sectional area, and δ-cell cross-sectional area, with the primary differences being the isolation of the corresponding fluorescent channel and the use of thresholding values to 50 for insulin-IR (red), glucagon-IR (green) and somatostatin-IR (green). The fifth program determined β-cell count and mean β-cell size. This script measured β-cell cross-sectional area, as described above, applied a Gaussian blur with a sigma of 1 to the DAPI staining, and counted the nuclei (as identified by DAPI staining) within that area. Afterwards, the program divided the β-cell cross-sectional area by the β-cell count to determine mean β-cell size[5].

FACS. The cells of interest were collected directly into pre-chilled 400 µL fresh RPMI-1640 media containing 10% FBS placed on ice. While more GKO islet cells than WT islet cells were subjected to FACS (GKO: 6.8×10^6 vs. WT: 5.7×10^5 WT), less singly suspended live GKO islet cells than WT islet cells were obtained after FACS (GKO: 1.0×10^5 vs. WT: 1.1×10^5). Although the reason for this is unclear, β-cells in large islets are more vulnerable than those in small islets to hypoxia (3) and treatments such as STZ (4) vs. smaller islets.

*β-cell apoptosis assay***.** Slides were washed three times with PBS, incubated with 3% normal donkey serum for 1 h at room temperature, and then incubated overnight in antibody cocktail of guinea pig anti-Insulin (DakoCytomation, Carpinteria, CA; A0564; diluted 1:300) + rabbit anti-cleaved caspase-3 (Cell Signaling Technology, Danvers, MA; Asp175 (5A1E) 9664, diluted 1:500) at 4°C. Next day, the slides were washed three times with PBS and incubated for 1 hr with secondary antibodies diluted 1:500: Alexa Fluor 594® goat anti-guinea pig (A11076) + Alexa Fluor 488® donkey anti-rabbit (A32790) at room temperature. Next, slides were washed with PBS and coverslipped with Vectashield-DAPI mounting medium (Vector Laboratories, Burlingame, CA). Fluorescence images of islets from one pancreas section per mouse were acquired at 20X magnification. Cleaved caspase-3^+Insulin$^+$ cells were identified by the presence of blue DAPI-stained nuclei surrounded by both cleaved caspase-3-IR and insulin-IR. The % of cleaved caspase-3^+ β-cells was calculated by dividing the total number of cleaved caspase-3^+Insulin$^+$ cells by the total number of Insulin$^+$ cells and multiplying by 100.

*β-cell proliferation assays***.** Slides were stained for Ki67-immunoreactivity and Insulin-immunoreactivity. Briefly, slides were washed three times with PBS, incubated with 3% normal donkey serum for 1 h at room temperature, and then incubated overnight in antibody cocktail of guinea pig anti-Insulin (DakoCytomation, Carpinteria, CA; A0564; diluted 1:300) + rabbit anti-Ki67 (Abcam, Cambridge, UK; ab15580; diluted 1:300) at 4°C. Next day, the slides were washed three times with PBS and incubated for 1 hr with secondary antibodies diluted 1:500: Alexa Fluor 594® goat anti-guinea pig (A11076) + Alexa Fluor 488® donkey anti-rabbit (A32790) at room temperature. Next, slides were washed with PBS and coverslipped with Vectashield-DAPI mounting medium (Vector Laboratories, Burlingame, CA). Fluorescence images of islets from two pancreas sections per mouse were acquired at 20X magnification. $Ki67^+Insulin^+$ cells were identified by the presence of blue DAPI-stained nuclei surrounded by both Ki67-IR and insulin-IR. The % of $Ki67^+$ β-cells was calculated by dividing the total number of $Ki67^+Insulin^+$ cells by the total number of $Insulin^+$ cells and multiplying by 100.

For the BrdU studies, pancreases were cryostat sectioned to a 14 µm-thickness at 50 µm intervals, and mounted on glass slides. Sections were first incubated with 2M HCl at 37°C for 20 min. Slides were then washed three times with PBS, incubated with 3% normal donkey serum for 1 h at room temperature, and incubated overnight with guinea pig anti-Insulin (DakoCytomation, Carpinteria, CA; A0564; diluted 1:300) + mouse anti-BrdU (Roche Diagnostic, Mannheim, Germany; SKU: 3042, diluted 1:50) at 4° C. Then, the slides were washed three times with PBS and incubated for 1 hr with secondary antibodies diluted 1:500: Alexa Fluor 594® goat anti-guinea pig (A11076) + Alexa Fluor 488® donkey anti-mouse (A21202) at room temperature. Next, slides were washed with PBS and coverslipped with Vectashield-DAPI mounting medium (Vector Laboratories, Burlingame, CA). Fluorescence images of islets from 2 pancreas sections per mouse were acquired at 20X magnification using a Leica microscope for quantitative analysis of $Brdu^+Insulin^+$ cells (with blue DAPI-stained nuclei) and $Insulin^+$ cells (with blue DAPI-stained nuclei). The % of $BrdU^+$ β-cells was calculated by dividing the total number of $BrdU^+Insulin^+$ cells by the total number of $Insulin^+$ cells and multiplying by 100.

Pancreatic insulin content. The entire pancreas was removed, washed with pre-chilled PBS, weighed, homogenized, and incubated in 6 mL ice-cold acid alcohol (0.18 M HCl in 70% ethanol) overnight at 4°C. The homogenate was centrifuged at 2400 rpm for 30 min at 4°C, the supernatant was stored at 4°C, and the pellet was resuspended in 6 mL fresh ice-cold acid alcohol. The acid alcohol incubation/centrifugation process was repeated over the next 3 days, after which the supernatants from the 4 days were pooled together. Pancreatic insulin content was measured in a 1:1000 diluted sample of the pooled supernatant for each mouse pancreas and normalized to body weight.

Blood collection and hormone analysis. Samples were immediately centrifuged at 4°C at 1,500 g for 15 min. HCl (final concentration 0.1 N) was added to tubes for ghrelin measurement. Samples were stored at −80°C. Acyl-ghrelin and insulin were measured using ELISA kits (Cat # EZRGRA-90K, Millipore-Merck, Burlington, MA; Cat # 90080, Crystal Chem, Downers Grove, IL) and LEAP2 was measured using an EIA kit (Cat# EK-075-40, Phoenix Pharmaceuticals, Inc. Burlingame, CA) with the aid of a BioTek PowerWave XS Microplate spectrophotometer (BioTek, Winooski, VT) and BioTek KC4 junior software, as described previously (5, 6).

Islet isolation and single cell dispersion. Following decapitation, pancreata were inflated *in situ* with freshly prepared collagenase (~ 5 mL of a 0.65 mg/mL solution) that was injected into the bile duct under a dissecting microscope (Supplemental Figure 9). Each inflated pancreas was dissected, placed in a 50 mL centrifuge tube, and then incubated in a 37°C water bath for 15

min. Four digested pancreases of each genotype were pooled together, after which islets were isolated by Histopaque density gradient (Sigma-Aldrich, Cat# 10771 and 11191) centrifugation (1200 rpm, 4°C for 30 min). All detectable islets were handpicked under a microscope (at 10x magnification) and placed into a 15 mL centrifuge tube containing 1 mL of 1x HBSS media (Gibco™, ThermoFisher Scientific, Waltham, MA; Cat# 14025-076). Single cell dispersion was then carried out as follows: islets were centrifuged (200 rpm, 4°C for 3 min), supernatant was removed, islets were incubated with 2 mL Accutase® solution (Innovative Cell Technologies, Inc. San Diego, CA; Cat# AT104) in a 37°C water bath for 15 min, after which cells were dispersed by pipetting up and down ten times using a 1 mL micropipette every 5 min x 3. The digestion was stopped by adding 10 mL of prewarmed RPMI-1640 (Gibco™, ThermoFisher Scientific, Waltham, MA; Cat# 11875-085) containing 10% FBS (Atlanta Biologicals, Flowery Branch, GA; Cat# S11150). The cell solution was filtered through a 40 µm cell strainer and centrifuged (200 rpm at 4°C for 3 min), after which the cells were resuspended with 2 mL of fresh media. A total of three washes were performed before the final resuspension of islet cells in 0.5 mL of serum containing media. The cells were placed on ice and then submitted for fluorescence-activated cell sorting (FACS) within 20 minutes.

Single cell RNA sequencing (scRNA-Seq). cDNA and single-cell libraries were prepared with the Chromium Next GEM Single Cell 3' Reagent Kit v3.1 (Dual Index) (10x Genomics, Pleasanton, CA), as per manufacturer's instructions: Cells in suspension were first prepared as gel beads in emulsion (GEMs) on a Single Cell 3' Chip G (10x Chromium) using the Chromium Controller (10x Genomics). For this, ~1,600-1,800/µL cells were loaded, aiming for 10,000 cells per channel. Barcoded RNA transcripts in each single cell were reverse transcribed within GEM droplets. cDNA in GEM reaction mixture was purified with DynaBeads MyOne™ Silane beads (ThermoFisher Scientific, Waltham, MA). Read 1 primer sequence was added and full-length barcoded cDNA was amplified for subsequent library construction. cDNA quality was verified by Agilent Tapestation 4200 (Agilent Technologies, Santa Clara, CA) using DNAHS 5000 tape, and concentration was determined with a Qubit 4 Fluorometer (ThermoFisher Scientific) using the DNA HS assay before sequencing. Sequencing libraries were prepared by fragmentation, end-repair, ligation with indexed adapters, and PCR amplification using the Chromium Single Cell 3' library kit v3.1 (10x Genomics). Nucleic acid was cleaned up using Ampure XP beads (Beckman Coulter Inc, Brea, CA). Post library preparation quality control was performed using DNA 1000 tape on the Agilent Tapestation 4200 and quantified by Qubit and real-time quantitative PCR on a LightCycler 96 System (Roche, Basel, Switzerland). Pooled libraries were sequenced at 1.6 pM on the Illumina NextSeq 2000 P2 100 Flowcell at a configuration of 28 x 90 x 10 x 10 base-pair, which codes for 28 base-pair Cell Barcodes, 90 base-pair Read, 10 base-pair unique molecular identifiers (UMI) and 10 base-pair Sample index. Cell Ranger Single-Cell Software Suite (v 5.0.1, 10X Genomics) was used to demultiplex cellular barcodes, map reads to the genome and transcriptome using the STAR aligner, and down-sample reads as required to generate normalized data across samples, producing a matrix of gene counts (20,690 genes in WT and 20,438 genes in GKO) vs. cells (7,792 cells in WT and 7,289 cells in GKO). The Barcode rank plots for both genotype samples are indicated in Supplemental Figure 10.

scRNA-Seq data processing and analysis. Cell feature-barcode matrices were processed with CellBender (default parameters unless otherwise specified) to remove ambient RNA reads and random barcode swapping from the dataset (CellBender v0.1.0; GKO samples; 7,300 expected cells, 20,000 total droplets included; WT samples; 7,800 expected cells, 20,000 total droplets included). We then analyzed the dataset with Seurat v4.1.1 software package(7) in R (version 4.0.2) using default Seurat parameters unless otherwise specified. Across all samples, we excluded cells in which we detected fewer than 500 genes or greater than 10% mitochondrial reads. We then merged and log-normalized the data, selected 2,000 most variable genes

("feature selection"), scaled expression of each gene, used Principal Component Analysis (PCA) for linear dimensionality reduction of the transcriptomes in highly variable gene space, clustered the cells using the Louvain algorithm (based on Euclidean distance in the PCA space comprising the first 20 PCs and with a resolution value of 1.2), and performed non-linear dimensionality reduction by Uniform Manifold Approximation and Projection (UMAP)(8) for visualization in two dimensions. We used DoubletFinder (v2.0.3) to identify and remove clusters representing cell doublets (parameters: 7.6% assumed doublet rate, 20 PCs, pN = 0.1, GKO; pK=0.005, WT; pK=0.27). DoubletFinder classifies each cell as either a singlet or a doublet. Clusters identified by DoubletFinder as composed of greater than 30% cell doublets were marked as potential doublet clusters. We then identified the cluster-enriched genes for each of these potential doublet clusters using Seurat's FindMarkers function. Potential doublet clusters for which we detected no cluster-specific marker genes were excluded from further analysis. After removing the suspected doublet clusters, the remaining cells were re-clustered, including the steps of feature selection, scaling expression of each gene, PCA, clustering with the top 15 PCs and resolution setting of 0.6. To match each cell cluster to a known cell type, we checked each cluster's expression of the following cell type marker genes and annotated them accordingly: α-Cells (*Gcg*); β-Cells (*Ins1*, *Ins2*); γ-Cells (*Ppy*), δ-Cells (*Sst*); endothelial-cells (*Pecam1* and *Plvap*); activated stellate-cells (*Pdgfra*, *Sparc,* and *C3*); quiescent stellate-cells (*Pdgfrb, Sparc,* and *Rgs5*); Gpr37l1+ Stellate-Cells (*Gpr37l1* and *Sparc*); resident (R)-macrophage-cells (*Cd86* and *Cx3cr1*); monocyte-derived (M)-macrophage-cells (*Cd86* and *Ly6c2*); S100a9+-cells (*S100a9*); ductal-cells (*Krt19*); and acinar-cells (*Cpb1*) (6, 9). The ductal-cell and acinar-cell clusters were deemed contaminants from exocrine tissue and so excluded from further analysis. The remaining cells were re-clustered, including the steps of feature selection, scaling expression of each gene, PCA, clustering with the top 20 PCs and resolution setting of 0.2. The clusters were re-labeled based on the marker genes and annotations listed previously. Genes differentially expressed between genotypes but within each cell cluster were identified by Wilcoxon Rank Sum test and MAST (10). Only genes which were differentially expressed to using both statistical tests, based on a Bonferroni adjusted P value < 0.05, are included in the Results.

Gene ontology over-representation analysis. Genes which differed significantly in expression between GKO and WT cells of each cell type were input to the WebGestalt web tool to identify over-represented Biological Process (no redundant) gene ontology terms, when compared to an Illumina Mouse 8 reference gene set. Figures were generated using WebGestalt.

In situ hybridization histochemistry. The slides were placed at −20 °C for 1 h and stored at −80 °C with desiccants. RNAscope (Advanced Cell Diagnostics, Newark, CA) was performed according to manufacturer's protocol (RNAscope® Multiplex Fluorescent Reagent Kit v2 Assay; Document number: 323100-USM, Rev Date: 02272019) *using commercial-available probes* against *Arg1* (RNAscope® Probe- Mm-Arg1-C2, Cat No. 403431-C2) and *Sst (*RNAscope® Probe- Mm-Sst-O1, Cat No. 482691*)*. Fluorescence images were acquired at 40X magnification using a Zeiss LSM880 Airyscan confocal microscope and qualitatively assessed for the colocalization of *Arg1* and *Sst* mRNA expression. Islets from n=4 mice of each genotype were assessed for consistency of findings.

Supplemental Figures

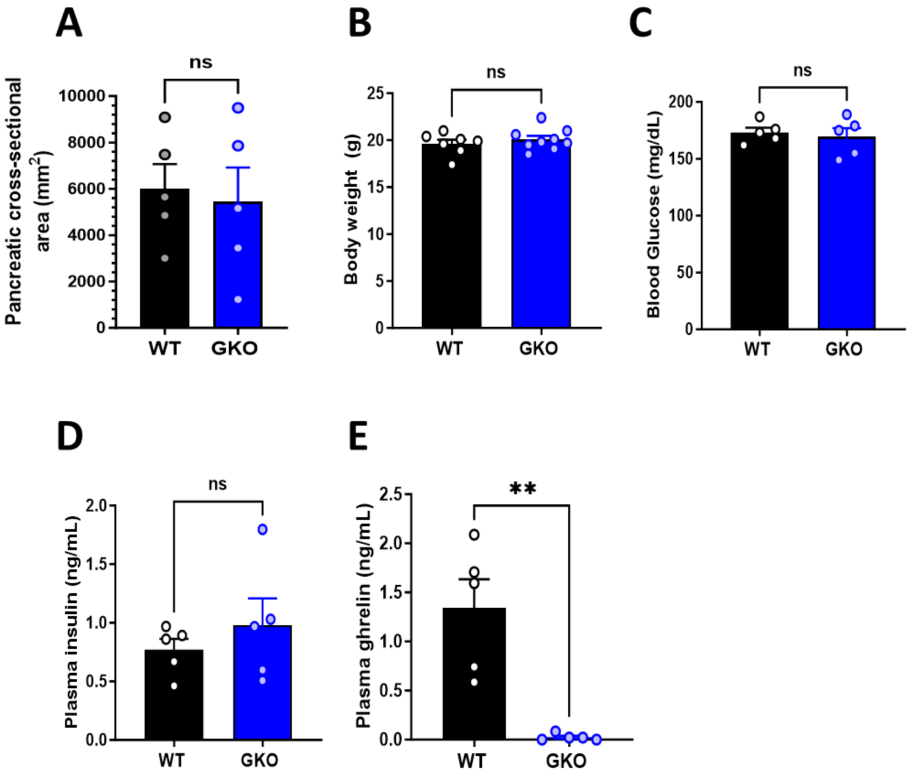

Supplemental Figure 1. Pancreatic cross-sectional area, body weight, blood glucose, plasma insulin, and plasma ghrelin in *ad lib*-fed WT and GKO mice. (**A**), Pancreatic cross-sectional area, (**B**), body weights, (**C**), blood glucose, (**D**), plasma insulin, and (**E**), plasma ghrelin of WT and GKO mice. n = 7-9 in (**B**) and n = 5 in (**A,C-E**); age = 9-12 wks-old. Data were analyzed by Student's unpaired *t* test. $^{**}P < 0.01$ or ns = not significant; related to Figure 1.

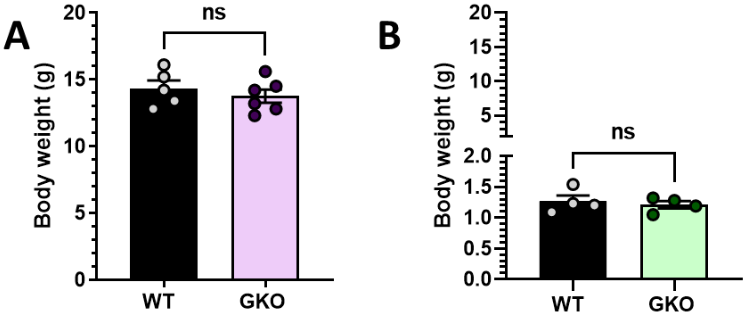

Supplemental Figure 2. Body weights of juvenile and neonate WT and GKO mice. (**A**), Body weights of juvenile WT and GKO mice and (**B**), body weights of neonate WT and GKO mice. n = 5-6 in (**A**) and n = 4 in (**B**). Data were analyzed by Student's unpaired *t* test. ns = not significant; related to Figure 2.

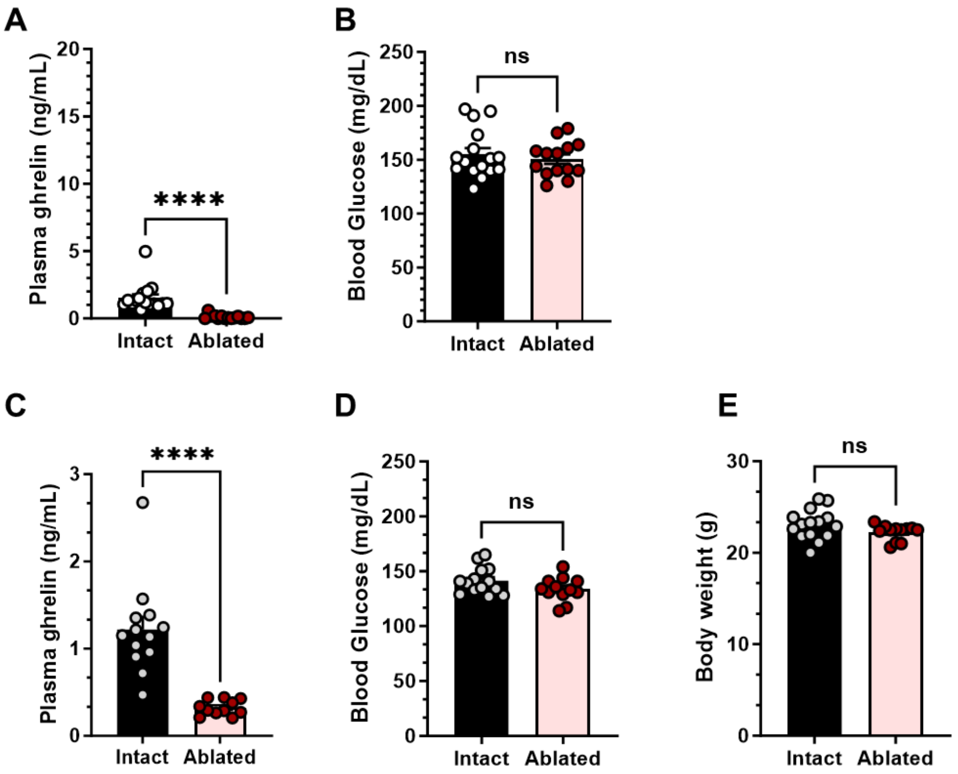

Supplemental Figure 3. Plasma ghrelin, blood glucose, and body weights of mice with or without ghrelin-cell ablation. (**A**), *Ad lib*-fed plasma ghrelin and (**B**), *ad lib*-fed blood glucose at 2 weeks post-DTX. (**C**), *Ad lib*-fed plasma ghrelin, (**D**), *ad lib*-fed blood glucose, and (**E**), body weights at the time of sacrifice. n = 11-16; age = 8-10 wks-old. Data were analyzed by Student's unpaired *t* test. ****$P < 0.0001$ or ns = not significant; related to Figure 4.

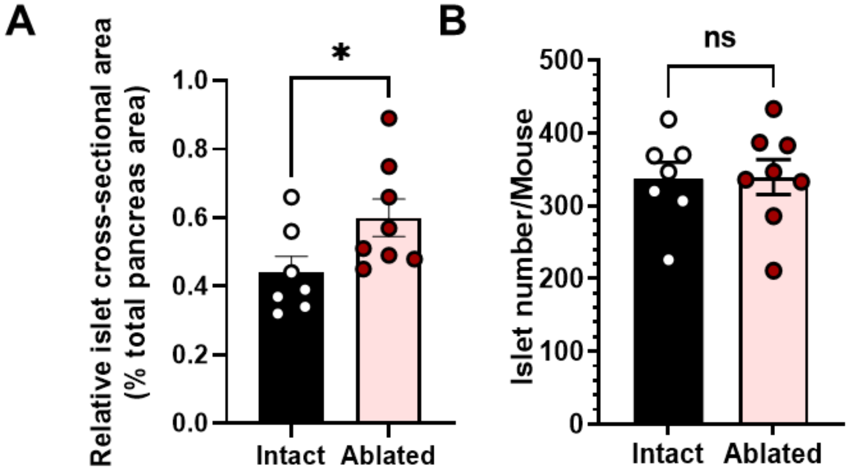

Supplemental Figure 4. Islet parameters from mice with Intact ghrelin-cells ("Intact") or ablated ghrelin-cells ("Ablated"). (**A**), Relative islet cross-sectional area. (**B**), Islet number from 4 pancreatic sections per mouse. n = 7-8; age = 10 wks-old. Data were analyzed by Student's unpaired *t* test. *$P < 0.05$; related to Figure 4.

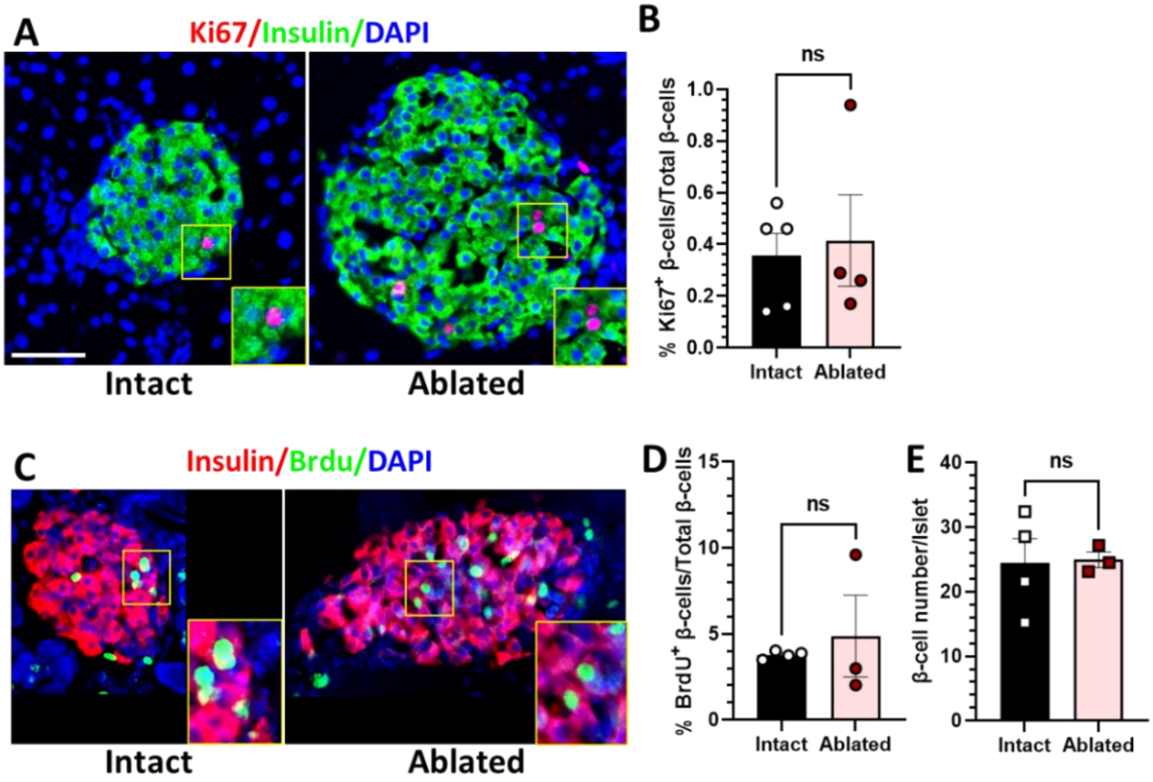

Supplemental Figure 5. β-cell proliferation from mice with Intact ghrelin-cells or ablated ghrelin-cells. (**A**), Representative islet images from mice with intact ghrelin cells ("Intact") and ablated ghrelin-cells ("Ablated") show Ki67-IR (in red) and insulin-IR (in green) with DAPI stained blue nuclei. (**B**), Percentage of Ki67^{+} β-cells/total β-cells. (**C**), Representative islet images from mice with intact ghrelin cells ("Intact") and ablated ghrelin-cells ("Ablated") show BrdU-IR (in green) and insulin-IR (in red) with DAPI stained nuclei as blue. (**D**), Percentage of BrdU^{+} β-cells/total β-cells. (**E**), β-cell number/islet. Scale bar in (A) = 100 μm (and applies to both large panels in **A** and **C**). n = 3-5; age = 7-10 wks-old. Data in (**B,D)** were analyzed by Student's unpaired *t* test. ns = not significant; related to Figure 4.

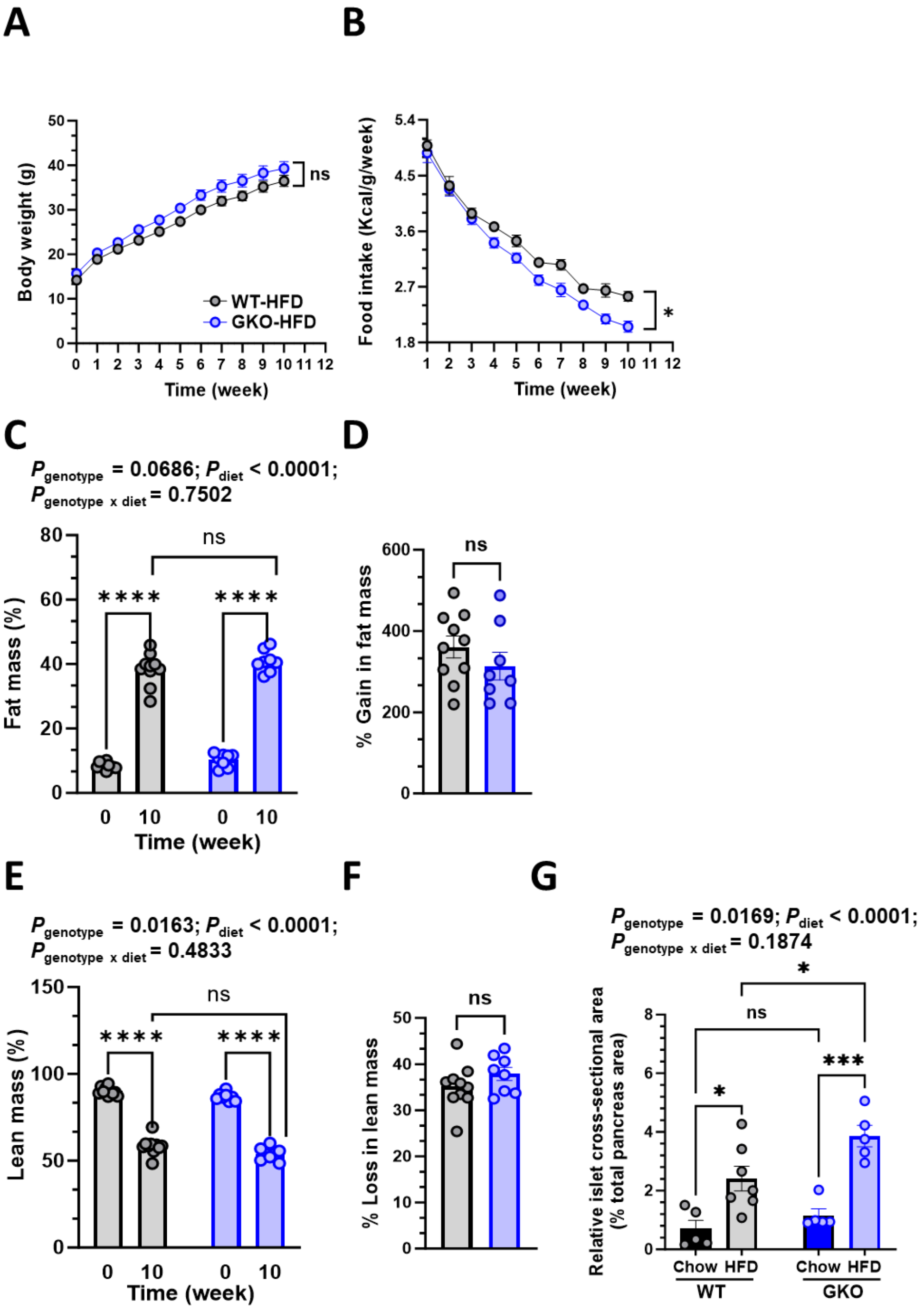

Supplemental Figure 6. Metabolic and islet-morphologic changes in WT and GKO mice fed standard chow or HFD. (**A**), Body weight, (**B**), Food intake, (**C**), Percent fat mass, (**D**), Percent gain in fat mass, (**E**), Percent lean mass, (**F**), Percent loss in lean mass, and (**G**), percentage relative islet cross-sectional area in WT and GKO mice fed HFD for 10 weeks. n = 12-13 mice in (**A-B**), n = 8-10 mice in (**C-F**), and n = 5-7 in (**E**); age = 10-14 wks-old. Data were analyzed by Student's unpaired t test in (**D,F**), 2-way repeated measures ANOVA followed by Bonferroni's multiple comparisons test in (**A-C,F**), and 2-way ANOVA followed by Tukey's multiple comparison test in (**E**). *P < 0.05; **P < 0.01, ***P < 0.001; ****P < 0.0001; ns = not significant; related to Figure 5.

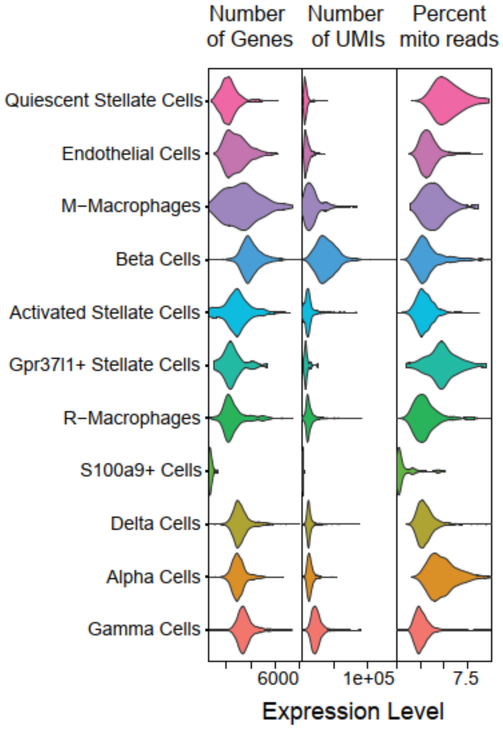

Supplemental Figure 7. Measures of transcriptome coverage and quality of cells included in scRNA-Seq (single cell RNA sequencing) analysis. Violin plots illustrating number of genes, number of Unique Molecular Identifiers (UMIs), and percentage of mitochondrial sequencing reads detected within each of the 11 identified clusters; related to Figure 7.

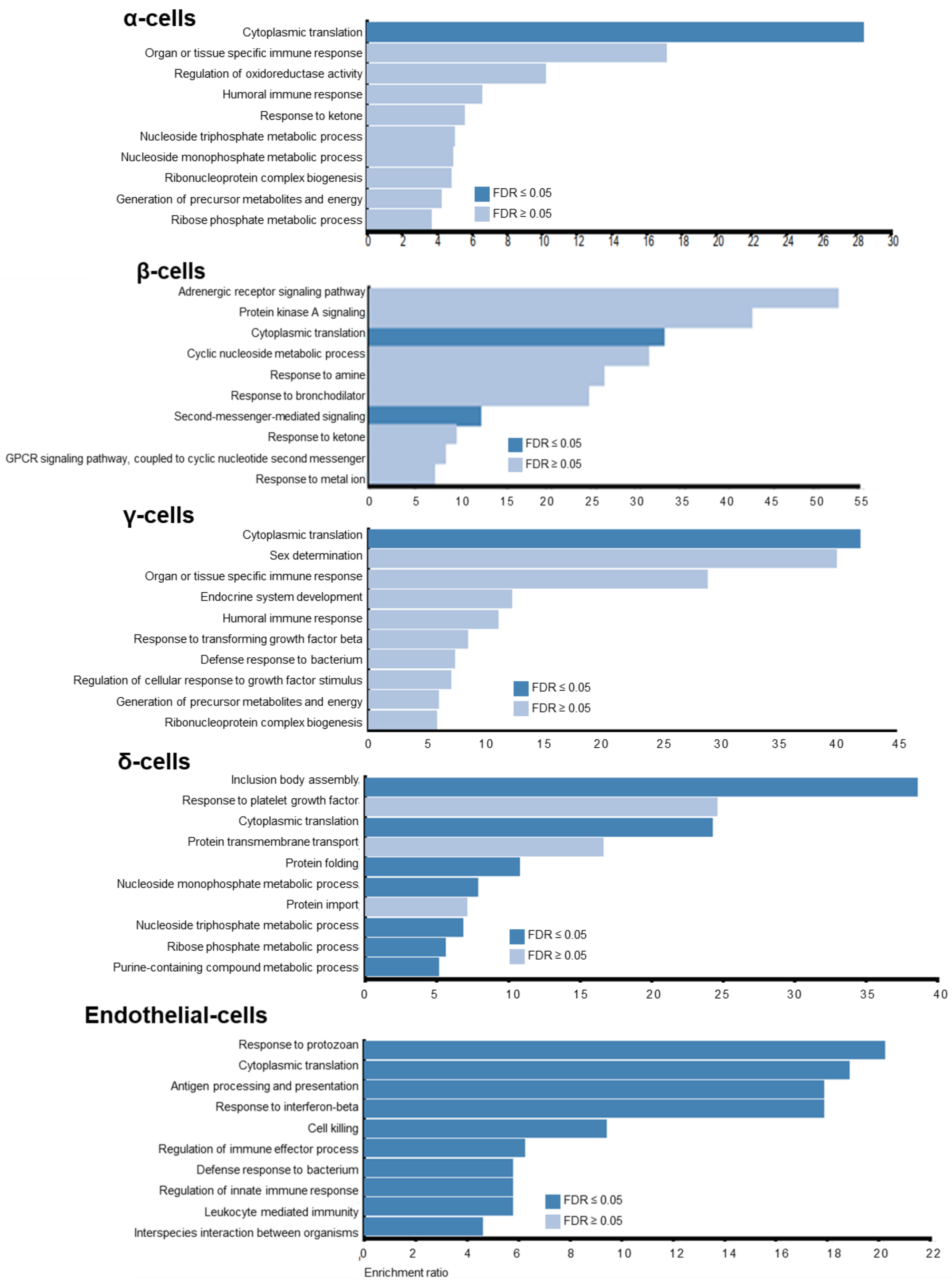

α-cells
Cytoplasmic translation
Organ or tissue specific immune response
Regulation of oxidoreductase activity
Humoral immune response
Response to ketone
Nucleoside triphosphate metabolic process
Nucleoside monophosphate metabolic process
Ribonucleoprotein complex biogenesis
Generation of precursor metabolites and energy
Ribose phosphate metabolic process
FDR ≤ 0.05
FDR ≥ 0.05
0 2 4 6 8 10 12 14 16 18 20 22 24 26 28 30
β-cells
Adrenergic receptor signaling pathway
Protein kinase A signaling
Cytoplasmic translation
Cyclic nucleoside metabolic process
Response to amine
Response to bronchodilator
Second-messenger-mediated signaling
Response to ketone
GPCR signaling pathway, coupled to cyclic nucleotide second messenger
Response to metal ion
FDR ≤ 0.05
FDR ≥ 0.05
0 5 10 15 20 25 30 35 40 45 50 55
γ-cells
Cytoplasmic translation
Sex determination
Organ or tissue specific immune response
Endocrine system development
Humoral immune response
Response to transforming growth factor beta
Defense response to bacterium
Regulation of cellular response to growth factor stimulus
Generation of precursor metabolites and energy
Ribonucleoprotein complex biogenesis
FDR ≤ 0.05
FDR ≥ 0.05
0 5 10 15 20 25 30 35 40 45
δ-cells
Inclusion body assembly
Response to platelet growth factor
Cytoplasmic translation
Protein transmembrane transport
Protein folding
Nucleoside monophosphate metabolic process
Protein import
Nucleoside triphosphate metabolic process
Ribose phosphate metabolic process
Purine-containing compound metabolic process
FDR ≤ 0.05
FDR ≥ 0.05
0 5 10 15 20 25 30 35 40
Endothelial-cells
Response to protozoan
Cytoplasmic translation
Antigen processing and presentation
Response to interferon-beta
Cell killing
Regulation of immune effector process
Defense response to bacterium
Regulation of innate immune response
Leukocyte mediated immunity
Interspecies interaction between organisms
FDR ≤ 0.05
FDR ≥ 0.05
0 2 4 6 8 10 12 14 16 18 20 22
Enrichment ratio

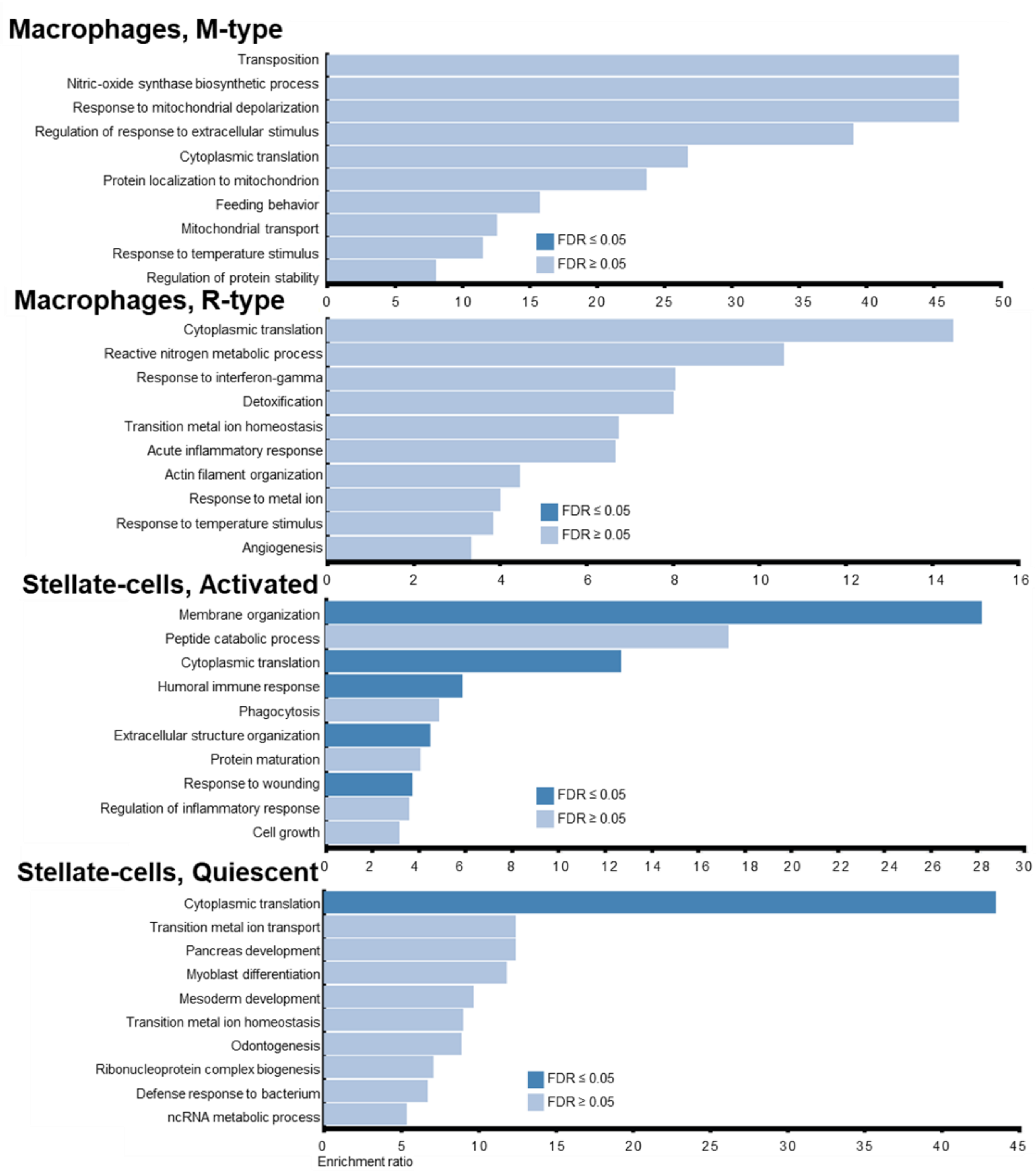

Supplemental Figure 8. GO-term analysis of genes differentially expressed between WT and GKO islet cells. GO-term analysis of genes differentially expressed between genotypes, for each cell type at false discovery rate ≤ 0.05 (dark blue) or ≥ 0.05 (light blue), related to Figure 7.

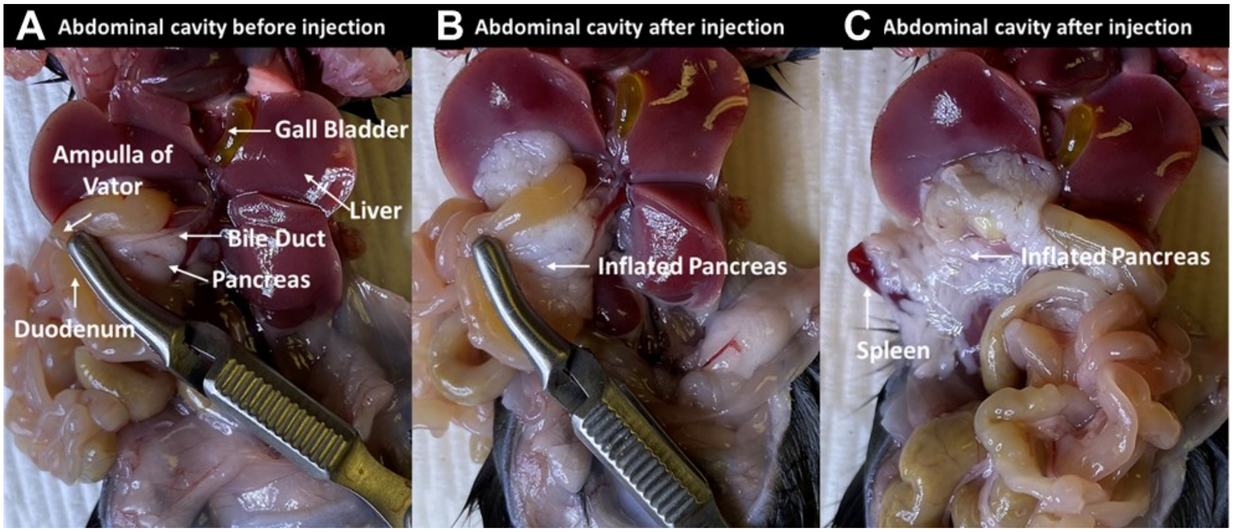

Supplemental Figure 9. Representative photos of in situ collagenase digestion of pancreata. (**A**), View of abdominal cavity before collagenase injection. (**B-C**), Views highlighting the inflated pancreas within the abdominal cavity following injection of up to 5 mL of collagenase solution (0.65 mg/mL) into the bile duct with (**B**) or without (**C**) the metal clamp in place.

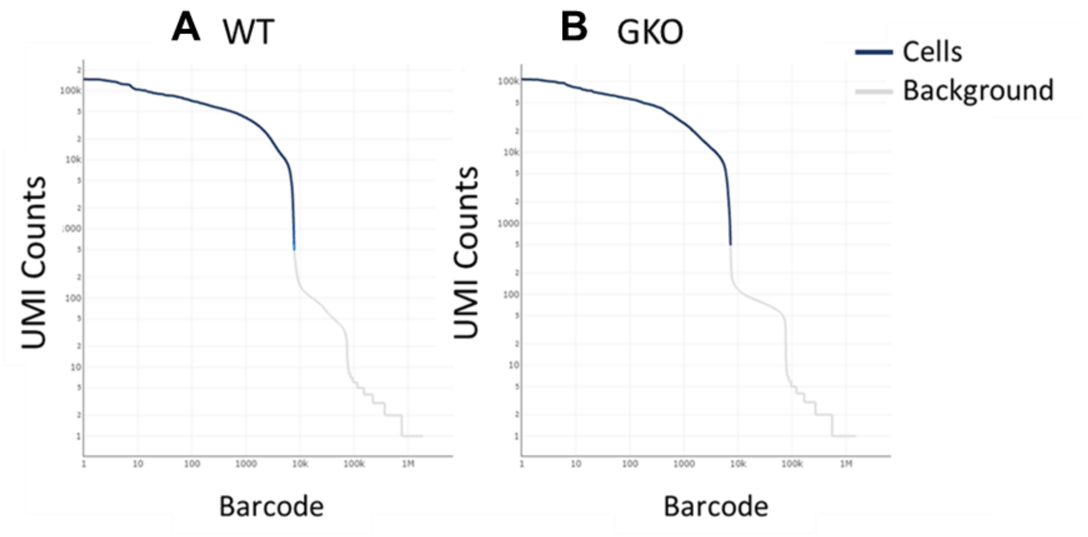

Supplemental Figure 10. Barcode Rank Plots. Barcode rank plots for (**A**), WT and (**B**), GKO single islet cell samples subjected to scRNA-Seq analysis.

Supplemental Table 1. Methodological details of published studies examining effects of ghrelin/GHSR on islet size

Study	Total # of islets analyzed per animal	Total # islets/ group	# of pancreatic sections/animal	# of animals /group	Blinded analysis
Hill et al (11)	All islets in every 5th or 10th section	N.A.	Every 5th or 10th section	3-4	N.A.
Dezaki et al (12)	N.A.	50-51	2-3, random	3	N.A.
Kurashina et al (13)	N.A.	79-107	N.A.	N.A.	N.A.
Pradhan et al (14)	12-14	36-42	N.A.	3	N.A.
Ma et al (15)	N.A.	≥120	Randomly selected	3	N.A.
Bando et al (16)	All islets in 5 sections	N.A.	5, >40μm apart	7	N.A.
Granata et al (17)	≤10	≤40	N.A.	4	N.A.
Shankar et al (18)	3-4	9-12	N.A.	3	N.A.
Mosa et al (19)	N.A.	22-30	3 or ≥5 non-consecutive sections[a]	3	N.A.
Baena-Nieto et al (20)	All islets in 2 sections	N.A.	2	5	Yes[b]
The current study	All islets in 4 sections (33-708)	484-3355	4, ≥50μm apart	4-8	Yes

[a]The paper describes in one place that 3 sections from each mouse and in another place that at least 5 pancreatic sections from each mouse were analyzed. [b]With the exception of the study by Baena-Nieto et al, none of the studies mentioned whether or not a blinded analysis was performed. Baena-Nieto et al performed a double-blinded analysis for insulitis, however it is not clear whether such also was used for the morphometric studies included in this table.

Supplemental Table 2. Differentially-expressed genes in α-cell cluster

Genes Upregulated in GKO α-Cells				
Gene ID[a]	Adj. P Value	Avg. Log2 FC GKO/WT[b]	% WT[c]	% GKO[d]
Txnip	5.9E-16	0.43104	84%	92%
Arg1	7.4E-15	0.32135	39%	56%
Gnas	1.7E-11	0.25790	100%	100%
Pde10a	1.2E-10	0.32728	54%	66%
Bambi	2.7E-09	0.29675	71%	79%
Ubc	1.1E-08	0.32357	99%	100%
Zcchc18	3.6E-08	0.30840	82%	87%
Btg2	9.2E-07	0.32529	79%	86%
Npas4	9.9E-07	0.35614	19%	31%
Gadd45b	1.8E-05	0.26852	84%	87%
Igfbp7	1.2E-04	0.25891	90%	93%
Swt1	1.0E-03	0.32222	61%	67%
Dnajb1	2.0E-03	0.31391	85%	90%

[a]Genes are listed in rank order of statistical significance, as based on the adjusted *P*-value.
[b]Avg. Log2 FC GKO/WT is a measure of the fold increase (left hand-columns; upregulation) or decrease (right hand columns; downregulation) in expression of the gene-of-interest within GKO α-cells, expressed in the Log_2 scale.
[c]% of WT α-cells expressing the gene-of-interest.
[d]% of GKO α-cells expressing the gene-of-interest.

Genes Downregulated in GKO α-Cells				
Gene ID[a]	Adj. P Value	Avg. Log2 FC GKO/WT[b]	% WT[c]	% GKO[d]
Rpl41	8.4E-234	-0.60196	100%	100%
Rps21	1.4E-228	-0.66473	100%	100%
Rpl37a	4.6E-211	-0.67267	100%	100%
Rps29	1.2E-194	-0.59211	100%	100%
Rpl37	2.8E-187	-0.65209	100%	100%
Rpl38	2.3E-162	-0.56401	100%	100%
Rpl36	5.9E-152	-0.64070	100%	100%
Rps28	4.4E-148	-0.65026	100%	99%
Rps27	2.3E-121	-0.58623	100%	100%
Rpl34	4.4E-117	-0.44837	100%	100%
Gm10076	3.3E-114	-0.58945	100%	100%
Rpl39	1.0E-112	-0.53241	100%	99%
Rplp2	1.6E-80	-0.38472	100%	100%
Rpl35a	2.3E-73	-0.33656	100%	100%
Rps8	8.8E-66	-0.32833	100%	100%
Rps26	1.9E-64	-0.41717	100%	100%
Sec61g	1.2E-61	-0.44584	99%	97%
Rpl35	3.5E-59	-0.39473	100%	99%
Atp5e	3.5E-58	-0.37455	100%	100%
Tomm7	3.2E-55	-0.44483	98%	92%
Uba52	2.0E-54	-0.46137	92%	79%
Rpl30	2.7E-53	-0.26522	100%	100%
Rps15a	3.6E-53	-0.29955	100%	100%
mt-Nd3	1.0E-51	-0.48450	99%	96%
Rpl36a	1.9E-44	-0.34982	100%	99%
Dpm3	3.7E-41	-0.42113	90%	75%
Uqcr11	7.5E-39	-0.37286	97%	94%
Rpl28	7.6E-37	-0.26016	100%	100%
Fau	1.9E-33	-0.25800	100%	100%
Por	2.2E-33	-0.43593	72%	51%
Ins2	4.7E-31	-1.68579	99%	90%
Rpl27	7.4E-31	-0.35508	94%	86%
Cox7c	1.4E-30	-0.30598	100%	98%
Rpl6	4.0E-29	-0.25609	100%	100%
Rps15	5.0E-28	-0.25621	100%	100%
Atp5j2	3.8E-27	-0.28975	99%	97%

Basp1	9.2E-27	-0.50239	77%	57%
Usmg5	8.0E-24	-0.31155	94%	88%
Fkbp5	8.9E-24	-0.35820	67%	46%
Romo1	1.2E-23	-0.31566	91%	85%
Tmsb15b2	1.6E-23	-0.47722	97%	93%
2010107E04Rik	3.4E-23	-0.31828	85%	73%
Smim22	2.6E-22	-0.33098	81%	65%
Lpin1	1.9E-21	-0.27684	39%	19%
Ndufa1	2.2E-20	-0.30982	92%	84%
Ins1	7.0E-20	-1.24222	88%	70%
Ndufa2	5.2E-19	-0.27167	97%	94%
Fam183b	1.8E-17	-0.27774	97%	95%
Myeov2	3.1E-17	-0.27057	90%	81%
Cox17	8.8E-17	-0.29053	85%	74%
Kcnk3	1.6E-16	-0.36531	75%	61%
Atp5k	2.3E-16	-0.27875	90%	79%
mt-Nd4l	4.1E-16	-0.30101	96%	91%
Snrpg	6.1E-16	-0.25415	65%	48%
Etv1	1.0E-15	-0.30972	96%	93%
Gpx3	4.3E-14	-0.36886	100%	99%
Sorbs2	2.1E-13	-0.33053	67%	55%
Wnt4	4.1E-11	-0.31059	98%	93%
Rhob	2.1E-08	-0.29699	66%	54%
Gm42418	7.1E-04	-0.26135	100%	100%
Iapp	3.7E-03	-0.29209	94%	88%

Supplemental Table 3. Differentially-expressed genes in β-cell cluster

Genes Upregulated in GKO β-Cells				
Gene ID[a]	Adj. P Value	Avg. Log2 FC GKO/WT[b]	% WT[c]	% GKO[d]
Gcg	3.68E-49	0.48813	100%	100%
Calm1	1.68E-35	0.28422	100%	100%
mt-Cytb	4.14E-33	0.28056	100%	100%
Dnajc3	1.97E-32	0.28433	99%	99%
Ssr2	3.33E-30	0.25604	98%	99%
Manf	2.63E-26	0.27496	99%	100%
Rsrp1	1.11E-24	0.25061	99%	100%
Sdf2l1	3.11E-24	0.32130	94%	95%
Pde10a	2.04E-19	0.25757	47%	62%
mt-Nd2	3.65E-16	0.26232	100%	100%
Gnas	1.11E-13	0.37348	100%	100%
Fkbp11	7.50E-13	0.25187	90%	92%
Ckb	5.28E-10	0.30016	55%	65%
Txnip	9.95E-10	0.33879	80%	85%
Actg1	5.39E-08	0.25240	99%	100%

Genes Downregulated in GKO β-Cells				
Gene ID[a]	Adj. P Value	Avg. Log2 FC GKO/WT[b]	% WT[c]	% GKO[d]
Rpl41	3.96E-131	-0.45381	100%	100%
Rpl37a	1.63E-111	-0.48004	100%	100%
Rps21	2.48E-100	-0.45284	100%	100%
Gm10076	1.12E-88	-0.42052	100%	100%
Rps29	5.62E-85	-0.41310	100%	100%
Rpl37	8.86E-81	-0.45587	100%	100%
Rpl38	6.03E-75	-0.37047	100%	100%
Rps28	1.51E-70	-0.40235	100%	100%
Rpl36	1.25E-62	-0.36055	100%	99%
Rpl39	4.49E-55	-0.32415	100%	100%
Dpm3	1.01E-54	-0.30973	98%	94%
Rps27	6.61E-54	-0.35767	100%	100%
Atp5e	1.82E-46	-0.26862	100%	100%
mt-Nd3	1.94E-40	-0.38255	100%	99%
Sec61g	3.03E-40	-0.26582	100%	100%
Sh3pxd2a	1.78E-39	-0.30048	83%	63%
G6pc2	8.87E-38	-0.43350	96%	94%
Rgs2	3.06E-34	-0.54683	98%	89%
Pde5a	3.79E-28	-0.30021	89%	76%
Mt1	6.55E-18	-0.76485	80%	65%
Mt2	8.46E-09	-0.30953	44%	30%

[a]Genes are listed in rank order of statistical significance, as based on the adjusted *P*-value.
[b]Avg. Log2 FC GKO/WT is a measure of the fold increase (left hand-columns; upregulation) or decrease (right hand columns; downregulation) in expression of the gene-of-interest within GKO β-cells, expressed in the Log_2 scale.
[c]% of WT β-cells expressing the gene-of-interest.
[d]% of GKO β-cells expressing the gene-of-interest.

Supplemental Table 4. Differentially-expressed genes in γ-cell cluster

Genes Upregulated in GKO γ-Cells				
Gene ID[a]	Adj. P Value	Avg. Log2 FC GKO/WT[b]	% WT[c]	% GKO[d]
mt-Cytb	1.11E-17	0.27129	100%	100%
Txnip	1.04E-11	0.60695	94%	95%
Cited2	1.20E-11	0.55954	72%	85%
Bambi	8.11E-08	0.32577	91%	94%
Gnas	2.60E-07	0.25508	100%	100%
Arl4d	4.49E-04	0.29376	52%	65%
Jun	7.52E-04	0.30792	99%	99%
Neat1	7.53E-02	0.33023	52%	64%

[a]Genes are listed in rank order of statistical significance, as based on the adjusted *P*-value.
[b]Avg. Log2 FC GKO/WT is a measure of the fold increase (left hand-columns; upregulation) or decrease (right hand columns; downregulation) in expression of the gene-of-interest within GKO γ-cells, expressed in the Log_2 scale.
[c]% of WT γ-cells expressing the gene-of-interest.
[d]% of GKO γ-cells expressing the gene-of-interest.

Genes Downregulated in GKO γ-Cells				
Gene ID[a]	Adj. P Value	Avg. Log2 FC GKO/WT[b]	% WT[c]	% GKO[d]
Rps21	1.41E-93	-0.66573	100%	100%
Rpl37a	9.86E-82	-0.62257	100%	100%
Rps29	3.79E-80	-0.58827	100%	100%
Rpl38	5.89E-78	-0.56213	100%	100%
Rpl41	1.96E-76	-0.53646	100%	100%
Rps28	7.48E-75	-0.59777	100%	100%
Rpl37	5.82E-70	-0.60042	100%	100%
Ins2	9.31E-55	-0.33417	100%	97%
Rpl39	1.24E-49	-0.49098	100%	100%
Rps27	2.57E-48	-0.52867	100%	100%
Gm10076	2.31E-47	-0.50948	100%	99%
Rpl34	1.02E-37	-0.39244	100%	100%
Rpl36	3.76E-37	-0.43477	100%	100%
Rplp2	5.36E-33	-0.41066	100%	100%
Rpl35	1.57E-32	-0.41682	99%	99%
Sec61g	3.22E-32	-0.38395	100%	100%
Uba52	1.14E-31	-0.43847	93%	76%
Dpm3	1.43E-30	-0.37991	92%	84%
Ins1	1.68E-30	-0.85618	98%	83%
Atp5e	2.46E-29	-0.35807	100%	100%
Rps26	3.19E-27	-0.35055	100%	100%
Tomm7	2.18E-23	-0.36608	98%	91%
Rpl35a	3.05E-22	-0.33097	100%	100%
Rps15a	3.76E-20	-0.30878	100%	100%
Tmem258	3.87E-20	-0.35349	97%	92%
Uqcr11	9.14E-20	-0.32061	97%	95%
Romo1	2.40E-19	-0.31284	95%	88%
Rpl36a	1.21E-18	-0.31251	100%	100%
Cox7c	5.20E-18	-0.27516	100%	99%
Rps8	4.00E-16	-0.27396	100%	100%
Myeov2	2.91E-15	-0.28898	94%	85%
Basp1	2.92E-15	-0.41298	80%	57%
mt-Nd3	2.58E-14	-0.32857	98%	95%
Usmg5	4.92E-14	-0.27063	97%	93%
Fau	1.79E-13	-0.25147	100%	100%
Rpl30	2.73E-13	-0.25675	100%	100%

Por	1.11E-11	-0.33243	81%	66%
Hspa5	3.91E-11	-0.34888	100%	100%
Rpl27	9.24E-11	-0.27198	95%	87%
Wnt4	3.69E-09	-0.34742	94%	90%
Iapp	4.18E-08	-0.49691	96%	90%
Tmsb15b2	0.02897	-0.27110	68%	56%

Supplemental Table 5. Differentially-expressed genes in δ-cell cluster

Genes Upregulated in GKO δ-Cells

Gene ID[a]	Adj. P Value	Avg. Log2 FC GKO/WT[b]	% WT[c]	% GKO[d]
Arg1	2.00E-58	0.59560	93%	98%
mt-Cytb	1.44E-40	0.27517	100%	100%
Cela1	1.91E-17	0.62526	36%	54%
Marcks	8.36E-17	0.29885	80%	88%
Txnip	1.01E-16	0.46704	85%	89%
Ptn	1.77E-12	0.38986	26%	40%
Errfi1	1.06E-11	0.29148	44%	57%
Resp18	1.76E-10	0.27764	97%	98%
Bambi	3.71E-06	0.25096	92%	94%
Pde10a	5.93E-05	0.25949	58%	66%

[a]Genes are listed in rank order of statistical significance, as based on the adjusted *P*-value.
[b]Avg. Log2 FC GKO/WT is a measure of the fold increase (left hand-columns; upregulation) or decrease (right hand columns; downregulation) in expression of the gene-of-interest within GKO δ-cells, expressed in the Log_2 scale.
[c]% of WT δ-cells expressing the gene-of-interest.
[d]% of GKO δ-cells expressing the gene-of-interest.

Genes Downregulated in GKO δ-Cells

Gene ID[a]	Adj. P Value	Avg. Log2 FC GKO/WT[b]	% WT[c]	% GKO[d]
Rpl41	1.34E-198	-0.53647	100%	100%
Rps29	1.56E-184	-0.56792	100%	100%
Rpl37a	2.91E-181	-0.55143	100%	100%
Rps21	5.74E-180	-0.57680	100%	100%
Rpl37	1.14E-147	-0.54341	100%	100%
Rpl38	1.12E-125	-0.49132	100%	100%
Rps28	1.18E-113	-0.53818	100%	99%
Rpl36	9.39E-103	-0.51201	100%	99%
Gm10076	1.35E-96	-0.54115	99%	97%
Rpl39	2.16E-92	-0.46548	100%	99%
Rps27	6.09E-89	-0.45707	100%	99%
Rpl34	1.51E-78	-0.36634	100%	100%
Rplp2	3.74E-51	-0.30750	100%	100%
Sec61g	9.08E-50	-0.39771	99%	98%
Atp5e	1.67E-48	-0.35122	100%	98%
Basp1	1.43E-47	-0.55533	88%	73%
Rpl35a	2.57E-46	-0.25259	100%	100%
mt-Nd3	7.21E-41	-0.48147	86%	69%
Cox7c	4.71E-39	-0.32413	99%	97%
Nudt4	3.37E-37	-0.53407	81%	61%
Tomm7	1.58E-36	-0.37112	94%	83%
Rps26	4.70E-36	-0.30322	99%	98%
Rpl35	9.52E-35	-0.32366	99%	97%
Romo1	1.20E-33	-0.35303	94%	86%
Smim22	2.52E-30	-0.36911	92%	83%
Dpm3	9.27E-30	-0.36379	85%	71%
Rpl36a	9.39E-29	-0.28676	99%	99%
Uba52	6.35E-28	-0.37195	81%	66%
Tmem258	2.26E-26	-0.35894	90%	79%
Uqcr11	6.70E-26	-0.29164	96%	90%
Usmg5	1.81E-24	-0.30361	92%	83%
B4galt6	2.94E-24	-0.38702	85%	73%
Tiam1	2.54E-23	-0.36063	37%	17%
Atp5k	3.38E-22	-0.29885	87%	74%
Cox17	3.74E-20	-0.29202	85%	71%
Ndufa1	1.52E-19	-0.29462	92%	85%

Atp5j2	3.13E-19	-0.25356	97%	94%
Myeov2	2.58E-17	-0.28056	86%	75%
Sh3pxd2a	3.74E-17	-0.26998	38%	20%
Rpl27	5.50E-17	-0.27204	87%	77%
Crip1	6.86E-17	-0.32113	65%	48%
Hspa8	3.36E-15	-0.25153	100%	100%
Clu	6.99E-15	-0.25940	98%	98%
Mrpl52	1.00E-13	-0.27073	83%	72%
Vcan	7.72E-13	-0.28875	29%	15%
Dnaja4	1.86E-12	-0.25184	62%	44%
Gatsl2	1.36E-11	-0.28946	83%	75%
Ramp1	2.34E-11	-0.29940	70%	56%
Fxyd3	2.46E-11	-0.36693	70%	58%
Rgs2	1.03E-10	-0.25042	98%	96%
mt-Nd4l	1.78E-10	-0.25049	86%	77%
Jund	1.22E-09	-0.33113	98%	96%
Robo2	2.50E-09	-0.27385	51%	37%
Ubr4	6.90E-09	-0.27753	69%	58%
Hspb1	1.39E-08	-0.54418	73%	61%
Dnajb1	6.05E-07	-0.38997	77%	65%
Ins2	7.39E-07	-1.14039	97%	90%
Ins1	9.87E-05	-0.86519	84%	72%
Ttr	0.00318	-0.32521	48%	59%

Supplemental Table 6. Differentially-expressed genes in Endothelial-cell cluster

Genes Upregulated in GKO Endothelial-Cells					Genes Downregulated in GKO Endothelial-Cells				
Gene ID[a]	Adj. P Value	Avg. Log2 FC GKO/WT[b]	% WT[c]	% GKO[d]	Gene ID[a]	Adj. P Value	Avg. Log2 FC GKO/WT[b]	% WT[c]	% GKO[d]
H2-D1	6.58E-20	0.42815	100%	100%	Sst	6.21E-96	-1.26280	81%	10%
H2-K1	1.47E-15	0.47416	100%	100%	Iapp	1.61E-87	-1.46076	69%	2%
B2m	6.34E-15	0.40034	100%	100%	Rps21	3.81E-83	-0.82553	100%	100%
H2-Ab1	2.27E-14	1.07379	3%	21%	Rps29	6.61E-81	-0.75279	100%	100%
Arl6ip5	1.03E-10	0.52822	33%	54%	Rpl37a	8.57E-62	-0.69081	100%	100%
H2-T23	2.89E-10	0.49321	89%	94%	Rpl37	1.25E-61	-0.69895	100%	99%
H2-Aa	5.10E-10	0.87511	1%	12%	Rpl41	5.68E-54	-0.61257	100%	100%
AW112010	2.20E-09	0.40659	95%	98%	Rps27	3.90E-48	-0.63014	99%	97%
Ppy	2.86E-09	0.28278	92%	97%	Rpl34	4.81E-45	-0.57705	100%	99%
Gbp3	1.06E-08	0.47698	21%	42%	Rpl38	4.09E-34	-0.63338	99%	94%
Ehd4	1.47E-08	0.34602	99%	99%	Rpl39	1.02E-33	-0.58018	100%	98%
Psmb8	2.83E-08	0.41768	91%	95%	Pyy	1.08E-33	-0.76263	74%	36%
Tnfsf10	2.88E-08	0.48158	69%	81%	Rps28	1.34E-32	-0.63370	98%	93%
Gbp4	7.46E-08	0.49877	31%	52%	Zbtb16	1.91E-32	-0.89261	50%	12%
H2-Q7	6.87E-07	0.62254	56%	71%	Rpl36	1.49E-31	-0.58498	98%	95%
Gbp2	9.45E-07	0.47914	35%	52%	Rpl35a	1.15E-28	-0.43046	100%	100%
Ntf3	2.68E-06	0.35893	17%	35%	Rpl35	6.02E-27	-0.56568	98%	94%
Ets2	8.54E-06	0.36088	71%	81%	Fkbp5	8.48E-27	-0.70831	79%	46%
Apoe	1.82E-05	0.80211	41%	59%	Rplp2	6.21E-26	-0.45030	100%	98%
Psmb9	2.19E-05	0.39809	70%	80%	Rps8	1.36E-25	-0.35691	100%	100%
Plscr2	3.57E-05	0.36117	95%	97%	Id3	5.50E-22	-0.65296	98%	93%
Iigp1	4.36E-05	0.75426	47%	63%	Gm10076	2.04E-21	-0.60795	92%	82%
Clec14a	4.36E-05	0.31108	92%	96%	Uba52	2.45E-19	-0.60372	84%	67%
H2-T22	4.69E-05	0.37743	52%	66%	Rps26	6.05E-19	-0.46974	98%	94%
Cd74	4.79E-05	1.69104	2%	12%	Rps15a	8.01E-17	-0.36060	100%	99%
Sdpr	6.92E-05	0.43774	88%	94%	Rplp1	2.71E-15	-0.27625	100%	100%
Gbp7	7.19E-05	0.48539	61%	74%	Rps12	4.30E-15	-0.32223	100%	99%
Casp12	8.15E-05	0.27507	11%	26%	Rpl36a	1.02E-14	-0.37393	99%	95%
Rsad2	8.75E-05	0.58696	66%	76%	Rgcc	7.75E-14	-0.46492	98%	99%
Ccrl2	1.05E-04	0.39581	22%	38%	Rps20	2.18E-13	-0.30486	100%	100%
Tap1	1.48E-04	0.44211	33%	48%	Id1	5.73E-13	-0.65822	90%	77%
H2-Q6	3.42E-04	0.50532	45%	59%	Rpl30	2.45E-12	-0.31487	100%	100%
Fmo2	9.21E-04	0.50984	34%	49%	Hes1	5.38E-12	-0.69143	87%	73%
Aplnr	1.19E-03	0.48389	13%	26%	Rpl6	2.06E-11	-0.31966	100%	98%
Psme2	1.26E-03	0.30503	75%	83%	Fau	7.26E-11	-0.27333	100%	100%
Bst2	1.28E-03	0.35122	89%	93%	Rps19	7.53E-11	-0.28348	100%	100%

Gas6	1.50E-03	0.30058	86%	91%
Plpp1	1.93E-03	0.29467	98%	99%
Igtp	2.33E-03	0.50869	20%	36%
Tcf15	2.80E-03	0.40877	13%	25%
Podxl	3.20E-03	0.28085	96%	98%
Tap2	5.06E-03	0.30900	62%	73%
H2-Q4	7.58E-03	0.38634	53%	64%
Rbms1	7.71E-03	0.27631	88%	93%
Unc45b	1.37E-02	0.30381	69%	79%
Degs1	1.54E-02	0.27927	76%	86%
Ly6a	1.87E-02	0.26165	99%	100%
Csf1	2.28E-02	0.26952	11%	23%
Il10rb	2.63E-02	0.30339	57%	71%
Rassf9	2.67E-02	0.27057	21%	34%
Jam3	4.60E-02	0.30790	77%	85%

[a]Genes are listed in rank order of statistical significance, as based on the adjusted *P*-value.
[b]Avg. Log2 FC GKO/WT is a measure of the fold increase (left hand-columns; upregulation) or decrease (right hand columns; downregulation) in expression of the gene-of-interest within GKO Endothelial-cells, expressed in the Log_2 scale.
[c]% of WT Endothelial-cells expressing the gene-of-interest.
[d]% of GKO Endothelial-cells expressing the gene-of-interest.

Hspa8	8.17E-11	-0.30558	100%	100%
Atp5e	1.20E-09	-0.39551	91%	76%
Snrpg	5.97E-09	-0.43828	59%	37%
Rpl28	7.75E-09	-0.26733	100%	100%
Cox7c	1.65E-07	-0.38548	89%	82%
Smad7	2.10E-07	-0.45960	78%	66%
Timp3	1.13E-06	-0.36458	99%	100%
Uqcr11	2.43E-06	-0.33966	84%	71%
Tmem204	7.66E-06	-0.34058	94%	86%
Cdkn1a	1.15E-05	-0.34253	94%	87%
Gcg	2.85E-05	-0.26105	91%	96%
Nr1d1	2.95E-05	-0.34573	31%	15%
Ace	7.61E-05	-0.48739	76%	63%
Ubl5	7.88E-05	-0.32155	86%	73%
Rpl23a	8.13E-05	-0.32744	87%	78%
Ucp2	8.18E-05	-0.41412	81%	73%
Gadd45b	8.99E-05	-0.54127	68%	52%
Sort1	1.72E-04	-0.35854	49%	31%
Atox1	1.90E-04	-0.26745	99%	96%
Rpl22	2.78E-04	-0.25336	99%	98%
Snrk	4.33E-04	-0.29590	97%	92%
Dpm3	5.38E-04	-0.37643	62%	46%
Tceb2	6.56E-04	-0.29459	91%	86%
Sgms1	8.00E-04	-0.35117	63%	46%
Hspg2	1.41E-03	-0.29017	97%	96%
Sec61g	1.74E-03	-0.30885	77%	66%
Crip1	1.75E-03	-0.42503	90%	85%
Cox6c	1.84E-03	-0.28665	93%	85%
Rpl15	2.10E-03	-0.28530	87%	83%
Mcf2l	2.94E-03	-0.30790	84%	74%
St13	3.09E-03	-0.33987	70%	58%
Sertad1	4.08E-03	-0.37439	75%	64%
Tnfaip2	8.28E-03	-0.33465	77%	66%
Sec14l1	1.14E-02	-0.27891	81%	73%
mt-Nd3	1.56E-02	-0.28083	83%	76%
2010107E04Rik	1.81E-02	-0.26324	53%	37%
Atp5k	1.83E-02	-0.27622	62%	49%
mt-Nd4l	1.87E-02	-0.29832	84%	79%
Chrm2	3.66E-02	-0.34294	47%	33%

Cdc42ep4	3.77E-02	-0.29228	51%	37%
Rpl27	3.88E-02	-0.28778	74%	65%
Bcl2l1	4.89E-02	-0.31360	55%	43%
Rin3	4.91E-02	-0.28630	36%	23%

Supplemental Table 7. Differentially-expressed genes in Activated Stellate-cell cluster

Genes Upregulated in GKO Activated Stellate-Cells					Genes Downregulated in GKO Activated Stellate-Cells				
Gene ID[a]	Adj. P Value	Avg. Log2 FC GKO/WT[b]	% WT[c]	% GKO[d]	Gene ID[a]	Adj. P Value	Avg. Log2 FC GKO/WT[b]	% WT[c]	% GKO[d]
Dcn	5.70E-21	0.54383	99%	100%	Iapp	1.97E-55	-1.15554	64%	10%
Lbp	1.45E-19	1.02680	36%	67%	Rpl41	5.45E-51	-0.71872	100%	100%
Itm2b	1.83E-17	0.39999	100%	100%	Rpl37a	8.43E-47	-0.72452	100%	99%
Fth1	6.73E-16	0.59247	100%	100%	Rps21	3.11E-46	-0.75079	100%	100%
Entpd2	1.49E-15	0.65139	68%	88%	Rps29	6.43E-41	-0.61400	100%	99%
Ly6a	1.29E-14	0.79814	54%	82%	Rpl36	1.11E-36	-0.67886	99%	96%
Rnase4	4.51E-13	0.57519	93%	93%	Gm10076	9.96E-33	-0.65289	98%	93%
Htra3	4.46E-09	0.65704	70%	85%	Rps27	7.71E-30	-0.57546	98%	96%
Ctsh	7.17E-09	0.55771	59%	78%	Rpl37	6.04E-28	-0.57636	100%	100%
Gsn	8.28E-09	0.78416	100%	100%	Rps28	2.50E-26	-0.60919	98%	98%
Anxa1	1.59E-08	0.65789	64%	77%	Rpl38	1.35E-22	-0.47686	100%	99%
Cd302	1.73E-08	0.53950	93%	96%	Rpl39	2.54E-22	-0.49631	100%	99%
Arpc1b	2.73E-08	0.54556	82%	89%	Rplp2	1.13E-19	-0.53310	99%	98%
Sepp1	2.88E-08	0.48709	94%	95%	Zbtb16	5.76E-18	-0.58408	48%	18%
Serping1	2.44E-07	0.44873	99%	100%	Uba52	4.04E-17	-0.57411	88%	68%
mt-Cytb	2.50E-07	0.25113	100%	100%	Postn	3.26E-16	-1.20750	50%	22%
C1s1	2.61E-07	0.42239	88%	92%	Rpl34	7.43E-16	-0.36363	100%	99%
Pmp22	2.91E-07	0.43267	79%	87%	Ttr	1.95E-15	-0.53501	34%	9%
Aldoa	4.07E-07	0.41773	89%	90%	Rps26	2.95E-15	-0.52040	99%	97%
Igfbp7	4.13E-07	0.41847	100%	100%	Pamr1	5.58E-13	-0.43101	33%	10%
Htra1	8.57E-07	0.46434	83%	90%	Rps8	1.07E-12	-0.42771	100%	100%
Myl12b	9.25E-07	0.48787	86%	93%	Rpl36a	1.19E-12	-0.48295	98%	97%
Ecm1	2.26E-06	0.38715	87%	92%	AY036118	1.05E-09	-0.60244	71%	46%
Cxcl14	2.78E-06	0.59229	19%	41%	Rpl35	1.34E-09	-0.38271	98%	98%
Ifitm3	5.24E-06	0.36251	100%	100%	Rps15	2.65E-09	-0.51507	96%	91%
C3	6.35E-06	0.54506	46%	67%	Rpl27	1.59E-08	-0.43371	87%	71%
Timp2	1.42E-05	0.33334	99%	98%	Fam213a	2.20E-08	-0.38885	31%	12%
Cyb5a	1.83E-05	0.33362	99%	98%	Atp5e	8.45E-08	-0.29760	98%	95%
Emp3	2.25E-05	0.44082	92%	93%	Tomm7	1.22E-07	-0.40197	88%	73%
Pfn1	2.60E-05	0.37437	94%	93%	Sec61g	1.64E-07	-0.30786	98%	94%
S100a10	3.01E-05	0.44291	93%	97%	Col12a1	1.85E-07	-0.45637	27%	10%
C7	3.61E-05	0.41496	28%	52%	mt-Nd3	3.49E-07	-0.44634	82%	69%
Gpx3	3.81E-05	0.51044	84%	91%	Cox7c	8.08E-07	-0.43512	95%	90%
Tspo	1.36E-04	0.47740	85%	89%	Uqcr11	9.71E-07	-0.32645	84%	68%
Pcolce	1.56E-04	0.36924	100%	99%	Tmem258	1.07E-06	-0.36643	80%	68%
Penk	2.15E-04	0.55790	73%	84%	Rplp1	1.29E-06	-0.39658	98%	98%

Cst3	2.78E-04	0.34264	100%	100%
Abca8a	3.94E-04	0.47306	69%	81%
Cd9	4.66E-04	0.54304	80%	83%
Fbln2	6.25E-04	0.51503	37%	54%
Igfbp4	7.01E-04	0.38685	99%	97%
Slit3	7.10E-04	0.43004	36%	54%
Spon2	7.96E-04	0.66355	20%	36%
G0s2	9.75E-04	0.64313	64%	73%
Hsd11b1	9.88E-04	0.61257	75%	78%
Prss23	1.22E-03	0.44477	67%	77%
Cygb	1.38E-03	0.41459	93%	91%
Ergic3	1.83E-03	0.38327	88%	89%
Ahnak	1.95E-03	0.37449	88%	89%
Ace	2.33E-03	0.48033	32%	49%
Slc25a4	2.67E-03	0.25492	97%	97%
Psap	3.51E-03	0.27467	94%	95%
Anxa2	3.74E-03	0.31891	93%	96%
Ifi27	3.75E-03	0.39545	85%	88%
Cpq	7.40E-03	0.31000	88%	90%
Lgi2	7.50E-03	0.49559	28%	44%
Clec3b	8.04E-03	0.39165	81%	87%
Adamts2	8.20E-03	0.37551	67%	76%
Ly6e	1.06E-02	0.30123	90%	95%
Gapdh	1.15E-02	0.34324	93%	95%
Ramp2	1.31E-02	0.38938	77%	83%
H2-D1	1.78E-02	0.32749	92%	94%
Mgst1	2.29E-02	0.45434	64%	72%
S100a16	2.53E-02	0.30394	87%	89%
Cnbp	2.73E-02	0.25277	90%	91%
Ndufb10	2.95E-02	0.34006	80%	80%
Vwa1	3.55E-02	0.41784	36%	51%
Sparcl1	4.85E-02	0.41565	82%	87%

[a]Genes are listed in rank order of statistical significance, as based on the adjusted *P*-value.
[b]Avg. Log2 FC GKO/WT is a measure of the fold increase (left hand-columns; upregulation) or decrease (right hand columns; downregulation) in expression of the gene-of-interest within GKO Activated Stellate-cells, expressed in the Log_2 scale.
[c]% of WT Activated Stellate-cells expressing the gene-of-interest.
[d]% of GKO Activated Stellate-cells expressing the gene-of-interest.

Usmg5	2.02E-06	-0.34418	74%	54%
2010107E04Rik	2.13E-06	-0.33440	80%	61%
Ins1	2.20E-06	-1.82495	43%	23%
Eln	2.79E-06	-1.00859	76%	64%
Dpm3	3.05E-06	-0.34134	85%	69%
Snrpg	4.37E-06	-0.30450	73%	51%
Arid5b	6.64E-06	-0.35342	61%	38%
Ctla2a	1.24E-05	-0.95072	36%	19%
Rasl11b	2.76E-05	-0.41072	36%	19%
Tagln	3.57E-05	-0.66671	32%	15%
Zfos1	3.61E-05	-0.28827	54%	33%
Gm42418	4.48E-05	-0.39948	90%	79%
Eif3j1	4.87E-05	-0.26054	50%	30%
Pcsk2	5.25E-05	-0.56996	23%	8%
Gadd45b	5.30E-05	-0.41433	81%	60%
Slc30a8	6.44E-05	-0.25676	15%	4%
Rps15a	9.80E-05	-0.26324	99%	99%
Rgs2	1.14E-04	-0.53679	37%	20%
Rps19	1.48E-04	-0.29920	99%	96%
Rps12	2.66E-04	-0.32395	98%	97%
Phex	2.76E-04	-0.26899	17%	6%
Rps17	7.25E-04	-0.28123	96%	94%
Slc25a25	8.10E-04	-0.30234	28%	13%
Snhg18	8.23E-04	-0.43555	91%	79%
Rpl23a	8.83E-04	-0.36267	88%	80%
Igfbp2	1.08E-03	-0.77231	17%	6%
Palld	1.71E-03	-0.48932	44%	28%
Ins2	2.11E-03	-2.01535	54%	36%
Cox17	2.41E-03	-0.25104	38%	21%
Hsph1	4.08E-03	-0.31030	80%	59%
Mef2c	4.32E-03	-0.27204	47%	28%
Romo1	4.39E-03	-0.25584	83%	68%
Inhba	4.96E-03	-0.30788	27%	14%
Mfap4	6.74E-03	-0.72611	63%	48%
Ero1lb	9.23E-03	-0.30514	11%	3%
Chga	1.31E-02	-0.26712	20%	8%
Gm10073	1.52E-02	-0.27786	82%	67%
Rps14	1.72E-02	-0.25849	100%	98%
Sertad1	1.78E-02	-0.33922	54%	36%

Atp5k	2.02E-02	-0.29875	71%	55%
Uqcr10	2.24E-02	-0.26429	84%	71%
Cited2	3.43E-02	-0.33219	54%	38%
Mfap2	4.18E-02	-0.41735	74%	64%

Supplemental Table 8. Differentially-expressed genes in Quiescent Stellate-cell cluster

Genes Upregulated in GKO Quiescent Stellate-Cells				
Gene ID[a]	Adj. P Value	Avg. Log2 FC GKO/WT[b]	% WT[c]	% GKO[d]
Slc11a1	3.84E-06	0.89368	26%	50%
Laptm4a	8.59E-05	0.39859	90%	97%
Fth1	1.29E-04	0.38511	100%	100%
Col14a1	1.48E-04	1.00969	9%	30%
Prss23	8.79E-04	0.83016	50%	67%
Rarres2	5.00E-03	0.31704	91%	98%
Cyb5a	5.36E-03	0.49351	65%	78%
Sdc1	8.02E-03	0.78889	15%	33%
Bmp4	8.52E-03	0.43897	9%	26%
Ilk	2.03E-02	0.43920	45%	64%
Mfap5	2.06E-02	0.72112	7%	23%
Arl6ip5	2.21E-02	0.38713	9%	25%
Dcn	2.66E-02	1.39874	13%	29%
Gstm1	3.16E-02	0.81160	71%	84%
Cxcl12	3.79E-02	0.65355	32%	51%
Cfh	4.39E-02	0.59653	45%	61%

Genes Downregulated in GKO Quiescent Stellate-Cells				
Gene ID[a]	Adj. P Value	Avg. Log2 FC GKO/WT[b]	% WT[c]	% GKO[d]
Iapp	4.33E-42	-1.03765	52%	4%
Rpl41	3.16E-29	-0.63805	100%	99%
Rps21	1.74E-28	-0.73091	100%	98%
Rps29	5.14E-28	-0.69132	100%	98%
Rpl37a	7.89E-27	-0.65889	100%	98%
Rpl37	8.16E-21	-0.62879	100%	99%
Rpl39	2.62E-18	-0.57922	98%	98%
Rps28	3.65E-18	-0.65601	99%	89%
Rpl36	1.46E-15	-0.56579	96%	93%
Rps27	4.83E-15	-0.60105	96%	93%
Gm10076	2.25E-14	-0.73365	91%	77%
Rpl35a	1.81E-13	-0.48475	100%	99%
Rpl38	1.20E-10	-0.54753	97%	93%
Rpl34	1.45E-10	-0.42425	99%	98%
Rps26	5.68E-09	-0.47854	96%	93%
Rps8	1.52E-08	-0.35436	100%	100%
Rps15a	1.72E-05	-0.36440	100%	100%
Rpl35	9.96E-05	-0.46320	91%	88%
Uba52	1.51E-04	-0.57136	64%	45%
Rpl27	1.57E-04	-0.49034	69%	53%
Zbtb16	4.04E-04	-0.59596	26%	9%
Cox6c	4.90E-04	-0.38549	96%	97%
Crip1	5.37E-04	-0.36881	100%	100%
Rplp2	5.48E-04	-0.39401	99%	96%
mt-Nd4l	1.43E-03	-0.44834	85%	75%
Rpl36a	1.50E-03	-0.38643	94%	91%
Uqcr11	3.46E-03	-0.38798	84%	74%
Rps12	1.00E-02	-0.35434	99%	98%
Rpl30	1.33E-02	-0.26438	100%	100%
Atp5l	2.70E-02	-0.33710	93%	86%

[a]Genes are listed in rank order of statistical significance, as based on the adjusted *P*-value.
[b]Avg. Log2 FC GKO/WT is a measure of the fold increase (left hand-columns; upregulation) or decrease (right hand columns; downregulation) in expression of the gene-of-interest within GKO Quiescent Stellate-cells, expressed in the Log_2 scale.
[c]% of WT Quiescent Stellate-cells expressing the gene-of-interest.
[d]% of GKO Quiescent Stellate-cells expressing the gene-of-interest.

Supplemental Table 9. Differentially-expressed genes in Gpr37l1^{+} Stellate-cell cluster

Genes Upregulated in GKO Gpr37l1+ Stellate-Cells						Genes Downregulated in GKO Gpr37l1+ Stellate-Cells				
**Gene ID**	**Adj. P Value**	**Avg. Log2 FC GKO/WT**	**% WT**	**% GKO**		**Gene ID**	**Adj. P Value**	**Avg. Log2 FC GKO/WT**	**% WT**	**% GKO**
No significantly differentially expressed genes detected						*No significantly differentially expressed genes detected*				

Supplemental Table 10. Differentially-expressed genes in (R)-Macrophage-cell cluster

Genes Upregulated in GKO R-Macrophage Cells					Genes Downregulated in GKO R-Macrophage Cells				
Gene ID[a]	Adj. P Value	Avg. Log2 FC GKO/WT[b]	% WT[c]	% GKO[d]	Gene ID[a]	Adj. P Value	Avg. Log2 FC GKO/WT[b]	% WT[c]	% GKO[d]
Tsc22d3	5.01E-11	0.73757	79%	93%	Iapp	1.09E-60	-1.50447	82%	15%
Itm2b	1.68E-09	0.48501	99%	100%	Sst	8.41E-30	-0.33408	95%	45%
Plekho1	7.17E-09	0.49502	55%	76%	Rps21	1.26E-28	-0.52352	99%	100%
Cyth4	4.98E-08	0.47458	56%	75%	Rpl41	7.70E-28	-0.55214	100%	100%
Clps	6.64E-08	1.10701	5%	24%	Rpl37a	1.11E-22	-0.43770	99%	100%
Klf2	8.33E-08	0.98170	74%	86%	Rps29	4.93E-16	-0.37167	100%	100%
mt-Atp6	4.75E-07	0.25706	100%	100%	Rpl37	5.92E-15	-0.43263	99%	100%
Ptp4a2	1.61E-06	0.33713	86%	93%	Rpl38	3.74E-14	-0.38695	99%	100%
Ppy	2.53E-06	0.30691	97%	99%	Uba52	6.39E-14	-0.56403	92%	81%
Rps6	2.58E-06	0.32037	99%	99%	Rps28	1.45E-13	-0.42704	99%	100%
Rpl5	3.70E-06	0.29614	98%	100%	Rps27	1.02E-12	-0.43620	100%	99%
Sepp1	4.92E-06	0.85928	84%	92%	Fkbp5	5.68E-12	-0.48128	63%	35%
Zfp36l2	5.42E-06	0.56692	79%	89%	Mt1	5.50E-11	-1.16619	96%	91%
Lyz2	1.65E-05	0.65769	89%	97%	Gm10076	1.79E-09	-0.45702	96%	93%
Emp3	4.44E-05	0.53011	58%	76%	Rpl36	4.23E-09	-0.37537	98%	99%
Rhob	5.48E-05	0.65067	83%	89%	Rpl39	1.56E-07	-0.38049	99%	99%
Ppia	1.31E-04	0.26391	99%	100%	Mt2	7.14E-07	-0.84965	50%	28%
Hpgd	2.31E-04	0.64850	53%	66%	Rplp2	1.24E-06	-0.28512	100%	100%
Serinc3	2.31E-04	0.43728	94%	97%	Gm17056	1.79E-06	-0.45844	52%	28%
Ifitm3	4.32E-04	0.65521	68%	79%	Tnfaip2	4.31E-06	-0.83067	48%	28%
Arpc1b	5.22E-04	0.33383	96%	97%	Mir155hg	1.10E-05	-0.32580	27%	10%
Cdc42	7.35E-04	0.27563	97%	99%	Ccrl2	2.61E-05	-0.66764	76%	59%
Aif1	1.37E-03	0.42147	93%	95%	Rpl35	6.88E-05	-0.30900	98%	99%
Hint1	1.57E-03	0.27903	91%	95%	Rrbp1	1.06E-04	-0.38876	94%	87%
Mrc1	1.94E-03	0.77815	33%	52%	Nlrp3	6.67E-04	-0.46380	36%	18%
Clec12a	2.21E-03	0.38367	41%	57%	Rpl34	7.51E-04	-0.25282	99%	100%
Trf	3.26E-03	0.56098	78%	84%	Sec61g	9.86E-04	-0.38488	92%	88%
Gpr34	3.45E-03	0.34276	17%	35%	Il1a	2.33E-03	-0.73792	35%	18%
Txnip	3.88E-03	0.35841	80%	87%	Ifrd1	3.80E-03	-0.48222	91%	81%
Marcks	4.26E-03	0.35807	88%	93%	Nfkbiz	5.65E-03	-0.66967	79%	68%
Ctsc	4.44E-03	0.37465	94%	96%	Zc3h12c	8.31E-03	-0.35873	34%	18%
Tmem176b	4.52E-03	0.38921	93%	93%	Tbc1d15	1.67E-02	-0.26681	46%	28%
Hprt	7.25E-03	0.38280	57%	66%	Arhgap22	1.76E-02	-0.26547	48%	29%
P2ry6	1.22E-02	0.34815	49%	65%	Glipr1	1.91E-02	-0.59868	70%	51%
Got1	1.63E-02	0.37297	48%	62%	E230013L22Rik	2.25E-02	-0.39916	31%	16%
Adgre1	1.65E-02	0.43419	70%	81%	Rel	2.30E-02	-0.54142	77%	64%

Stk17b	2.23E-02	0.49050	67%	77%
Fcer1g	2.79E-02	0.25340	95%	99%
Atp5h	2.99E-02	0.25768	92%	96%
Clec4b1	3.06E-02	0.33088	13%	28%
Igfbp4	3.67E-02	0.67754	24%	39%

Tnf	2.30E-02	-1.07272	65%	52%
Odc1	2.64E-02	-0.42051	69%	50%

[a]Genes are listed in rank order of statistical significance, as based on the adjusted *P*-value.
[b]Avg. Log2 FC GKO/WT is a measure of the fold increase (left hand-columns; upregulation) or decrease (right hand columns; downregulation) in expression of the gene-of-interest within GKO (R)-Macrophage-cells, expressed in the Log_2 scale.
[c]% of WT (R)-Macrophage-cells expressing the gene-of-interest.
[d]% of GKO (R)-Macrophage-cells expressing the gene-of-interest.

Supplemental Table 11. Differentially-expressed genes in (M)-macrophage-cell cluster

Genes Upregulated in GKO M-Macrophage Cells				
Gene ID[a]	Adj. P Value	Avg. Log2 FC GKO/WT[b]	% WT[c]	% GKO[d]
Hspa1a	2.32E-06	1.35442	79%	96%
Hspa1b	5.42E-06	1.29344	89%	98%
Klf6	3.93E-05	1.05282	87%	96%
Gcg	2.92E-03	1.47565	84%	98%
Ppy	6.55E-03	1.14834	97%	100%
S100a6	8.18E-03	1.94437	31%	62%
Swt1	1.82E-02	1.09480	68%	80%
Pmaip1	2.01E-02	0.96353	42%	67%
Fcer2a	3.95E-02	0.80102	2%	27%

[a]Genes are listed in rank order of statistical significance, as based on the adjusted *P*-value.
[b]Avg. Log2 FC GKO/WT is a measure of the fold increase (left hand-columns; upregulation) or decrease (right hand columns; downregulation) in expression of the gene-of-interest within GKO (M)-Macrophage-cells, expressed in the Log_2 scale.
[c]% of WT (M)-Macrophage-cells expressing the gene-of-interest.
[d]% of GKO (M)-Macrophage-cells expressing the gene-of-interest.

Genes Downregulated in GKO M-Macrophage Cells				
Gene ID[a]	Adj. P Value	Avg. Log2 FC GKO/WT[b]	% WT[c]	% GKO[d]
Sst	2.04E-24	-1.12371	96%	16%
Iapp	3.29E-22	-0.66840	88%	11%
Rpl41	7.66E-08	-0.59222	100%	99%
Rps29	4.30E-07	-0.44711	100%	99%
Rpl37	9.77E-07	-0.56087	100%	98%
Tomm7	3.18E-05	-0.55630	97%	81%
Gm10076	3.36E-05	-0.62732	100%	96%
Rps21	3.86E-05	-0.55049	100%	99%
Nuggc	8.54E-04	-0.41017	28%	2%
Rps28	1.33E-03	-0.44262	100%	99%
Rgs13	1.71E-03	-0.81651	22%	0%
Hypk	4.44E-03	-0.41007	67%	32%
Pyy	8.33E-03	-0.25437	96%	53%
Aicda	8.87E-03	-0.72819	20%	0%
Dynll1	1.27E-02	-0.56973	98%	93%
Rplp2	1.59E-02	-0.40427	100%	99%
Hist1h4d	1.67E-02	-0.51426	55%	24%
Myl4	2.02E-02	-0.42910	33%	7%
Cox6c	2.04E-02	-0.47657	93%	93%
Psmb3	2.11E-02	-0.52066	93%	79%
Rpl27	2.15E-02	-0.42779	98%	94%
Atp5k	4.50E-02	-0.52618	86%	68%
Cep55	4.64E-02	-0.31610	46%	16%

Supplemental Table 12. Differentially-expressed genes in S100a9^{+}-cell cluster

Genes Upregulated in GKO S100a9+ Cells				
**Gene ID**	**Adj. P Value**	**Avg. Log2 FC GKO/WT**	**% WT**	**% GKO**
No significantly differentially expressed genes detected				

Genes Downregulated in GKO S100a9+ Cells				
**Gene ID[a]**	**Adj. P Value**	**Avg. Log2 FC GKO/WT[b]**	**% WT[c]**	**% GKO[d]**
Iapp	7.68E-07	-3.91441	100%	0%
Cd24a	3.51E-04	-3.91823	75%	0%

[a]Genes are listed in rank order of statistical significance, as based on the adjusted *P*-value.
[b]Avg. Log2 FC GKO/WT is a measure of the fold increase (left hand-columns; upregulation) or decrease (right hand columns; downregulation) in expression of the gene-of-interest within GKO S100a9+-cells, expressed in the Log_2 scale.
[c]% of WT S100a9+-cells expressing the gene-of-interest.
[d]% of GKO S100a9+-cells expressing the gene-of-interest.

Supplemental Table 13. Program code to re-scale pixel brightness and remove background “noise” from islet images

```
import cv2 as cv
import numpy as np
from pathlib import Path
from scipy import stats
from tqdm import tqdm

def apply_sig(arr, med, std):
    ex = lambda x: x/(1+np.exp(-1*(4.5/std)*(x-2*med)))
    alt = []
    for row in arr:
        r = []
        for val in row:
            r.append(ex(val))
        alt.append(r)
    return alt

def clean(img_):
    green = img_.T[1]
    a = np.where(green.T < 180, green.T, np.nan)
    mode = stats.mode(a, axis=None, nan_policy='omit')[0][0]
    std = np.std(green.T)
    med = np.median(green.T)
    avg = np.average(green.T)
    if mode+std < med or mode-std > med:
        # print("CHANGED")
        mode = med-(avg-med)
    alt = apply_sig(green.T, med, std)
    new = np.array(alt).T
    img_.T[1] = new
    return img_

if __name__ == '__main__':
    dir_ = r"path\to\folder"
    images = []
    indexes = []
    pathlist = [x for x in Path(dir_).glob('*.PNG')]
    for i in tqdm(pathlist):
        file_name = str(i).split('\\')[-1]
        img = cv.imread(dir_ + '\\' + str(file_name))
        try:
            img = cv.cvtColor(img, cv.COLOR_BGR2RGB)
        except:
            continue
        out = clean(img.copy())
        images.append(out)
        indexes.append(file_name)

    out_ = dir_ + " Cleaned\\"
    try:
```

```
        Path(out_).mkdir()
    except FileExistsError:
        print('OS exists already!')
    for img, i in zip(images, indexes):
        img = cv.cvtColor(img, cv.COLOR_RGB2BGR)
        cv.imwrite(out_ + i, img)
```

Supplemental Table 14. Program codes to analyze islet morphology

Program 1: Computer code to measure islet cross-sectional area

```
from ij import IJ, ImagePlus
from loci.plugins import BF

IJ.run("Set Scale...", "distance=118 known=100 unit=um global")
for i in range(120):
    insulin = []
    glucagon = []
    DAPI = []
    try:
        a = IJ.getImage()
        f1 = a.title
    except:
        break
    IJ.run("Split Channels")
    blue_ = IJ.getImage()
    blue_.close()
    IJ.run("Merge Channels...", "c1=[" + f1 + " (red)] c2=[" + f1 + " (green)] create")
    clutter = IJ.getImage()
    IJ.run("RGB Color")
    IJ.run("8-bit")
    clutter.close()

    IJ.setMinAndMax(20, 255)
    IJ.setThreshold(20, 255)
    IJ.run("Convert to Mask")
    IJ.run("Analyze Particles...", "size=51-550000 show=Outlines display include
summarize record add")
    clutter_2 = IJ.getImage()
    clutter_2.close()
    clutter_3 = IJ.getImage()
    clutter_3.close()
```

Program 2: Computer code to measure β-cell cross-sectional area

```
from ij import IJ, ImagePlus
from loci.plugins import BF

IJ.run("Set Scale...", "distance=118 known=100 unit=um global")
for i in range(120):
    insulin = []
    glucagon = []
    DAPI = []
    try:
        a = IJ.getImage()
```

```
        f1 = a.title
    except:
        break
    IJ.run("Split Channels")
    blue_ = IJ.getImage()
    blue_.close()
    green_ = IJ.getImage()
    green_.close()
    IJ.run("8-bit")

    IJ.setMinAndMax(50, 255)
    IJ.setThreshold(50, 255)
    IJ.run("Convert to Mask")
    IJ.run("Analyze Particles...", "size=51-550000 show=Outlines display include 
summarize record add")
    clutter = IJ.getImage()
    clutter.close()
    clutter_2 = IJ.getImage()
    clutter_2.close()
```

Program 3: Computer code to measure α-cell cross-sectional area

```
from ij import IJ, ImagePlus
from loci.plugins import BF

IJ.run("Set Scale...", "distance=118 known=100 unit=um global")
for i in range(120):
    insulin = []
    glucagon = []
    DAPI = []
    try:
        a = IJ.getImage()
        f1 = a.title
    except:
        break
    IJ.run("Split Channels")
    blue_ = IJ.getImage()
    blue_.close()
    IJ.run("8-bit")

    IJ.setMinAndMax(50, 255)
    IJ.setThreshold(50, 255)
    IJ.run("Convert to Mask")
    IJ.run("Analyze Particles...", "size=51-550000 show=Outlines display summarize 
record add")
    clutter = IJ.getImage()
```

```
    clutter.close()
    clutter_2 = IJ.getImage()
    clutter_2.close()
    green_ = IJ.getImage()
    green_.close()
```

Program 4: Computer code to measure δ-cell cross-sectional area

```
from ij import IJ, ImagePlus
from loci.plugins import BF

IJ.run("Set Scale...", "distance=118 known=100 unit=um global")
for i in range(120):
   insulin = []
   glucagon = []
   DAPI = []
   try:
      a = IJ.getImage()
      f1 = a.title
   except:
      break
   IJ.run("Split Channels")
   blue_ = IJ.getImage()
   blue_.close()
   green_ = IJ.getImage()
   green_.close()
   IJ.run("8-bit")

   IJ.setMinAndMax(50, 255)
   IJ.setThreshold(50, 255)
   IJ.run("Convert to Mask")
   IJ.run("Analyze Particles...", "size=21-550000 show=Outlines display include
summarize record add")
   clutter = IJ.getImage()
   clutter.close()
   clutter_2 = IJ.getImage()
   clutter_2.close()
```

Program 5: Computer code to measure β-cell size and β-cell count

```
from ij import IJ, ImagePlus
from loci.plugins import BF
from ij.measure import ResultsTable
from ij.plugin.frame import RoiManager
import ij
from ij.gui import ShapeRoi
import csv
```

```
array = {"File Name": [], "Mean Cell Size": [], "Cell Count": [], "Area": []}
continuing = True
while True:
    a = IJ.getImage()
    f1 = a.title
    directory = a.getOriginalFileInfo().directory
    IJ.run("Split Channels")
    IJ.selectWindow(f1 + ' (blue)')
    IJ.run("Close")
    IJ.selectWindow(f1 + ' (green)')
    IJ.run("Close")
    IJ.selectWindow(f1 + ' (red)')
    IJ.run("8-bit")

    IJ.setMinAndMax(50, 255)
    IJ.setThreshold(50, 255)
    IJ.run("Convert to Mask")
    IJ.run("Analyze Particles...", "size=51-550000 show=Outlines display include
summarize record add")
    joinkies = IJ.getImage()
    joinkies.close()
    gg = IJ.getImage()
    gg.close()
    IJ.selectWindow("Summary")
    summary = ResultsTable.getResultsTable()
    IJ.renameResults("Results")
    summary = ResultsTable.getResultsTable()
    array['Area'].append(float(summary.getColumn(summary.getColumnIndex("Total
Area"))[-1]))
    IJ.open(directory + f1)
    IJ.selectWindow(f1)
    IJ.run("Split Channels")
    IJ.selectWindow(f1 + ' (green)')
    IJ.run("Close")
    IJ.run("Clear Results")

    IJ.selectWindow(f1 + ' (blue)')
    blue_channel = IJ.getImage()
    rm = RoiManager().getInstance()
    rois = rm.getRoisAsArray()
    shape_united = None
    roi1 = None
    if len(rois) > 1:
        for roi in rois:
            if roi1:
                if shape_united:
                    roi2 = ShapeRoi(roi)
```

```
                shape_united = shape_united. or (roi2)
            else:
                roi2 = ShapeRoi(roi)
                shape_united = roi1. or (roi2)
        else:
            roi1 = ShapeRoi(roi)
    else:
        blue_channel.setRoi(rois[0])

    IJ.selectWindow(f1 + ' (blue)')
    IJ.run("Clear Outside")
    IJ.run("Gaussian Blur...", "sigma=1 scaled")
    IJ.run("Find Maxima...", "prominence=10 strict output=[Count]")
    res = ResultsTable.getResultsTable()

    array['Cell Count'].append(float(res.getColumn(res.getColumnIndex("Count"))[-1]))
    array['File Name'].append(str(f1))

    array["Mean Cell Size"].append(array["Area"][-1] / array["Cell Count"][-1])

    IJ.selectWindow("ROI Manager")
    IJ.run("Close")
    IJ.selectWindow("Results")
    IJ.run("Close")
    IJ.selectWindow(f1 + ' (blue)')
    IJ.run("Close")
    IJ.selectWindow(f1 + ' (red)')
    IJ.run("Close")
    try:
        a = IJ.getImage()
    except:
        break
with open(directory + 'Cell Sizes.csv', 'wb') as output:
    writer = csv.writer(output)
    writer.writerow(['File Name', "Mean Cell Size", "Area", "Cell Count"])
    for i in range(len(array['File Name'])):
        writer.writerow([array['File Name'][i], array["Mean Cell Size"][i], array["Area"][i],
array["Cell Count"][i]])
```

Supplemental Table 15. Validation of the computer programs used to analyze islet morphology

Analysis from 50 random islets	Program results	Manual results	*P*-value
Mean islet cross-sectional area (mm^2)	11.84 ± 2.18	11.73 ± 2.19	ns
Mean β-cell cross sectional area (mm^2)	10.72 ± 1.97	10.76 ± 1.98	ns
Mean α-cell cross-sectional area (mm^2)	2.85 ± 0.32	2.64 ± 0.29	ns
Circularity	0.59 ± 0.00	0.61 ± 0.00	ns
Ferret's diameter (μm)	136.66 ± 0.01	135.7 ± 0.01	ns

Program results = data obtained using the computer programs. Manual results = data obtained using the computer mousepad together with ImageJ software to trace the perimeter of each islet, after which the above measurements were made with the assistance of the ImageJ 'Analyze' tool. For this, 50 random islets of various sizes were analyzed by both methodologies, and the data obtained were compared by Student 't' test. *P*-values are indicated. ns = not significant.

Reference used in Supplemental Methods:

1. Buch T, Heppner FL, Tertilt C, Heinen TJ, Kremer M, Wunderlich FT, et al. A Cre-inducible diphtheria toxin receptor mediates cell lineage ablation after toxin administration. *Nat Methods.* 2005;2(6):419-26.
2. Engelstoft MS, Park WM, Sakata I, Kristensen LV, Husted AS, Osborne-Lawrence S, et al. Seven transmembrane G protein-coupled receptor repertoire of gastric ghrelin cells. *Mol Metab.* 2013;2(4):376-92.
3. Sigmundsson K, Ojala JRM, Ohman MK, Osterholm AM, Moreno-Moral A, Domogatskaya A, et al. Culturing functional pancreatic islets on alpha5-laminins and curative transplantation to diabetic mice. *Matrix Biol.* 2018;70:5-19.
4. Hahn M, van Krieken PP, Nord C, Alanentalo T, Morini F, Xiong Y, et al. Topologically selective islet vulnerability and self-sustained downregulation of markers for beta-cell maturity in streptozotocin-induced diabetes. *Commun Biol.* 2020;3(1):541.
5. Shankar K, Takemi S, Gupta D, Varshney S, Mani BK, Osborne-Lawrence S, et al. Ghrelin cell-expressed insulin receptors mediate meal- and obesity-induced declines in plasma ghrelin. *JCI Insight.* 2021;6(18).
6. Gupta D, Dowsett GKC, Mani BK, Shankar K, Osborne-Lawrence S, Metzger NP, et al. High Coexpression of the Ghrelin and LEAP2 Receptor GHSR With Pancreatic Polypeptide in Mouse and Human Islets. *Endocrinology.* 2021;162(10).
7. Hao Y, Hao S, Andersen-Nissen E, Mauck WM, 3rd, Zheng S, Butler A, et al. Integrated analysis of multimodal single-cell data. *Cell.* 2021;184(13):3573-87 e29.
8. McInnes L, and Healy J. UMAP: Uniform manifold approximation and projection for dimension reduction. *ArXiv e-prints.* 2018;1802.03426.
9. Hu H, Zakharov PN, Peterson OJ, and Unanue ER. Cytocidal macrophages in symbiosis with CD4 and CD8 T cells cause acute diabetes following checkpoint blockade of PD-1 in NOD mice. *Proc Natl Acad Sci U S A.* 2020;117(49):31319-30.
10. Finak G, McDavid A, Yajima M, Deng J, Gersuk V, Shalek AK, et al. MAST: a flexible statistical framework for assessing transcriptional changes and characterizing heterogeneity in single-cell RNA sequencing data. *Genome Biol.* 2015;16:278.
11. Hill JT, Mastracci TL, Vinton C, Doyle ML, Anderson KR, Loomis ZL, et al. Ghrelin is dispensable for embryonic pancreatic islet development and differentiation. *Regul Pept.* 2009;157(1-3):51-6.
12. Dezaki K, Sone H, Koizumi M, Nakata M, Kakei M, Nagai H, et al. Blockade of pancreatic islet-derived ghrelin enhances insulin secretion to prevent high-fat diet-induced glucose intolerance. *Diabetes.* 2006;55(12):3486-93.

13. Kurashina T, Dezaki K, Yoshida M, Sukma Rita R, Ito K, Taguchi M, et al. The beta-cell GHSR and downstream cAMP/TRPM2 signaling account for insulinostatic and glycemic effects of ghrelin. *Sci Rep.* 2015;5:14041.
14. Pradhan G, Wu CS, Villarreal D, Lee JH, Han HW, Gaharwar A, et al. beta Cell GHS-R Regulates Insulin Secretion and Sensitivity. *Int J Mol Sci.* 2021;22(8).
15. Ma X, Lin Y, Lin L, Qin G, Pereira FA, Haymond MW, et al. Ablation of ghrelin receptor in leptin-deficient ob/ob mice has paradoxical effects on glucose homeostasis when compared with ablation of ghrelin in ob/ob mice. *Am J Physiol Endocrinol Metab.* 2012;303(3):E422-31.
16. Bando M, Iwakura H, Ariyasu H, Hosoda H, Yamada G, Hosoda K, et al. Transgenic overexpression of intra-islet ghrelin does not affect insulin secretion or glucose metabolism in vivo. *Am J Physiol Endocrinol Metab.* 2011.
17. Granata R, Volante M, Settanni F, Gauna C, Ghe C, Annunziata M, et al. Unacylated ghrelin and obestatin increase islet cell mass and prevent diabetes in streptozotocin-treated newborn rats. *J Mol Endocrinol.* 2010;45(1):9-17.
18. Shankar K, Gupta D, Mani BK, Findley BG, Osborne-Lawrence S, Metzger NP, et al. Ghrelin Protects Against Insulin-Induced Hypoglycemia in a Mouse Model of Type 1 Diabetes Mellitus. *Frontiers in endocrinology.* 2020;11:606.
19. Mosa R, Huang L, Li H, Grist M, LeRoith D, and Chen C. Long-term treatment with the ghrelin receptor antagonist [d-Lys3]-GHRP-6 does not improve glucose homeostasis in nonobese diabetic MKR mice. *Am J Physiol Regul Integr Comp Physiol.* 2018;314(1):R71-R83.
20. Baena-Nieto G, Lomas-Romero IM, Mateos RM, Leal-Cosme N, Perez-Arana G, Aguilar-Diosdado M, et al. Ghrelin mitigates beta-cell mass loss during insulitis in an animal model of autoimmune diabetes mellitus, the BioBreeding/Worcester rat. *Diabetes Metab Res Rev.* 2017;33(1).
